# Prognostic Implications of Novel Ten-Gene Signature in Uveal Melanoma

**DOI:** 10.3389/fonc.2020.567512

**Published:** 2020-10-30

**Authors:** Huan Luo, Chao Ma, Jinping Shao, Jing Cao

**Affiliations:** ^1^Department of Anatomy, College of Basic Medicine, Zhengzhou University, Zhengzhou, China; ^2^Charité—Universitätsmedizin Berlin, Corporate Member of Freie Universität Berlin, Humboldt-Universität zu Berlin, and Berlin Institute of Health, Berlin, Germany; ^3^Klinik für Augenheilkunde, Charité—Universitätsmedizin Berlin, Corporate Member of Freie Universität Berlin, Humboldt-Universität zu Berlin, and Berlin Institute of Health, Berlin, Germany; ^4^Charité—Universitätsmedizin Berlin, BCRT—Berlin Institute of Health Center for Regenerative Therapies, Berlin, Germany

**Keywords:** uveal melanoma, gene signature, risk score, prognosis, biomarkers

## Abstract

**Background:** Uveal melanoma (UM) is the most common primary intraocular cancer in adults. Genomic studies have provided insights into molecular subgroups and oncogenic drivers of UM that may lead to novel therapeutic strategies.

**Methods:** Dataset TCGA-UVM, download from TCGA portal, were taken as the training cohort, and dataset GSE22138, obtained from GEO database, was set as the validation cohort. In training cohort, Kaplan–Meier analysis and univariate Cox regression model were applied to preliminary screen prognostic genes. Besides, the Cox regression model with LASSO was implemented to build a multi-gene signature, which was then validated in the validation cohorts through Kaplan–Meier, Cox, and ROC analyses. In addition, the correlation between copy number aberrations and risk score was evaluated by Spearman test. GSEA and immune infiltrating analyses were conducted for understanding function annotation and the role of the signature in the tumor microenvironment.

**Results:** A ten-gene signature was built, and it was examined by Kaplan–Meier analysis revealing that significantly overall survival, progression-free survival, and metastasis-free survival difference was seen. The ten-gene signature was further proven to be an independent risk factor compared to other clinic-pathological parameters via the Cox regression analysis. Moreover, the receiver operating characteristic curve (ROC) analysis results demonstrated a better predictive power of the UM prognosis that our signature owned. The ten-gene signature was significantly correlated with copy numbers of chromosome 3, 8q, 6q, and 6p. Furthermore, GSEA and immune infiltrating analyses showed that the signature had close interactions with immune-related pathways and the tumor environment.

**Conclusions:** Identifying the ten-gene signature (SIRT3, HMCES, SLC44A3, TCTN1, STPG1, POMGNT2, RNF208, ANXA2P2, ULBP1, and CA12) could accurately identify patients' prognosis and had close interactions with the immunodominant tumor environment, which may provide UM patients with personalized prognosis prediction and new treatment insights.

## Introduction

Uveal melanoma (UM) is the most common primary intraocular cancer in adults, and the second most common melanoma subtype after cutaneous melanoma, accounting for 5% of all melanomas (1–3). Treatment approaches for primary UM include surgery and radiotherapy, which can often achieve excellent local tumor control (4). Nevertheless, nearly half of UM patients still develop tumor metastasis, mainly in the liver (3). Metastases have a predilection for the liver and once they have developed, median survival is about 1 year (5). Existing treatments for UM are not effective against tumor metastases (6), therefore, most research shifted their efforts on the development of targeted therapies or immunotherapy methods, such as immune checkpoint inhibitors, vaccination, or adoptive T cell therapy (7–11). Identifying potential biomarkers of UM may provide critical information for early detection of relapse or treatment (12). At present, although some studies have clarified some important genes and pathways of UM, the prognosis of it remains poor (12–14). Therefore, there is an urgent need to reveal new markers to assess UM prognosis.

During the past few decades, genetic or epigenetic alterations have been confirmed to be associated with the tumorigenesis and progression of UM (14). Gene mutations and chromosomal copy number variations are closely related to UM prognosis. According to reports, GNAQ and GNA11 mutations can promote cell proliferation and metastasis (15). The loss of one copy of chromosome 3 (monosomy 3) in UM is associated with an increased risk of metastasis and poor prognosis (16). In addition, other chromosomal abnormalities have been shown to correlate with poor prognosis and these include 6q loss, lack of 6p gain, 1p loss, and 16q loss (16–20). Therefore, further exploration of gene mutation and copy number variation in UM can provide incisive information for prognosis.

Here, we conduct comprehensive mining of the TCGA and GEO database to determine the minimum number of potentially robust genes that can be used to predict the prognosis of UM patients. Importantly, we used the LASSO algorithm, which can effectively analyze high-dimensional sequencing data (21). Besides, we assessed the accuracy of this ten-gene signature and validated it by compared to variants of chromosomes 3 and 8q, and testing in a validation cohort. Moreover, GSEA and immune infiltrating analyses were conducted to explore the role of the signature in the tumor microenvironment.

## Materials and Methods

### Data Mining From the Cancer Genome Atlas (TCGA) and Gene Expression Omnibus (GEO) Databases

The gene expression profiles of UM from 80 patients, along with their clinical and curated survival data were downloaded from TCGA Xena Hub (https://tcga.xenahubs.net) with cohort name: TCGA-UVM. Besides, we researched the GEO database by setting a filter: (1) more than 60 cases; (2) with expression profiling data; (3) with survival data. Finally, GSE22138 with 63 cases was chosen for this study. In our research, TCGA-UVM was used as the training cohort, while GSE22138 was taken as the validation cohort. The research was conducted in accordance with the Declaration of Helsinki, and was approved by the Ethics Committee of Zhengzhou University.

### Identification and Validation of Prognostic Gene Signature

To begin with, in the training cohort, Kaplan-Meier analysis was applied to screen the potential prognostic genes based on overall survival, disease-specific survival, and progression-free survival, respectively. Only genes that showed significant in all overall, disease-specific, and progression-free survival analyses were considered to pass Kaplan–Meier analysis screening. *P* < 0.0001 in the log-rank test was considered as significant. Also, univariate Cox regression analysis was performed on the training cohort to find potential prognostic genes (*p* < 0.0001). Same as before, only genes that showed significant in all overall, disease-specific, and progression-free survival analyses were considered to pass univariate Cox regression analysis screening. The intersected genes of identified in Kaplan–Meier and univariate Cox analyses were then entered into the LASSO Cox regression model analysis, which was implemented in the training cohort utilizing R software and the “glmnet” package. 10-fold cross-validation was applied to detect the best penalty parameter lambda (21–24). Based on the detected optimal lambda, we could obtain a list of prognostic genes with correlation coefficients from gene expression and patient survival data.

The risk score of each patient was calculated by a linear combination of the expression level of each gene weighted by its multivariate LASSO regression coefficient. Using the median risk score as the cut-off point, the patients in the training cohort were distributed to high-risk or low-risk groups, and Kaplan–Meier analysis was applied to evaluate the survival difference between the two groups. Besides, Cox and ROC analyses were conducted to further assess the prognostic value of the gene signature in training cohort. Subsequently, we validated the prognostic value of the gene signature in the validation cohort. The same formula was conducted to compute risk scores like that in the training cohort. Kaplan–Meier, Cox, and ROC analyses were implemented as described earlier.

In UM, chromosomal aberrations and gene mutations have been shown to be closely related to treatment options and prognosis. In Robertson's research, the status of chromosome 3, 8q, 6q, 6p, and 1p of each patient in the TCGA-UVM cohort has been studied and specifically described (16). The Spearman rank correlation coefficient was applied to assess the correlation between copy number aberrations and risk score, further evaluating the prognostic value of the gene signature identified in this study. *P* < 0.05 was considered statistically significant.

### Gene Set Enrichment Analysis

The Hallmark (v7.1) and KEGG (v7.1) gene set collections were obtained from the Molecular Signatures Database v7.1 download page (https://www.gsea-msigdb.org/gsea/downloads.jsp). GSEA was performed based on the downloaded gene set collections using GSEA software (v4.0.3, https://www.gsea-msigdb.org/). The training cohort was taken for GSEA to reveal the functions and pathways in the differentially expressed genes between high-risk and low-risk groups. According to the GSEA User Guide, gene sets with | NES |> 1, NOM *p* < 0.05, and FDR *q* < 0.25 were considered significant.

### Correlation of Risk Score With the Proportion of 20 Kinds of Tumor-Infiltrating Immune Cells (TICs)

The CIBERSORT calculation method was used to estimate the 20 kinds of TICs abundance distribution of all tumor samples in the training cohort. After quality filtering (*p* < 0.05) was performed on all the samples of TCGA-UVM, 36 samples were selected for the next analyses.

### Statistical Analysis

All statistical calculations in this study were performed in R software. Kaplan–Meier analysis was performed to examine the prognostic differences between the groups, and the *p*-value was checked in the log-rank test. Univariate and multivariate Cox analyses were conducted to illustrate the relationship between the gene signature risk score and UM prognosis. The ROC curves were plotted with the “pROC” R package, to assess the sensitivity and specificity of the risk score for prognosis prediction. The area under the ROC curve (AUC) was used as an indicator of prognostic accuracy. The correlation between 20 kinds of TICs were examined by Pearson coefficient test. Spearman coefficient test was used for the correlation test between the TICs proportion and risk score. The Wilcoxon rank-sum test verified the differentiation of 20 kinds of immune cells between low and high-risk groups. In addition to noted before, all analyses *p* < 0.05 was a statistically significant threshold.

## Results

### Clinical Characteristics

**Figure 1:**
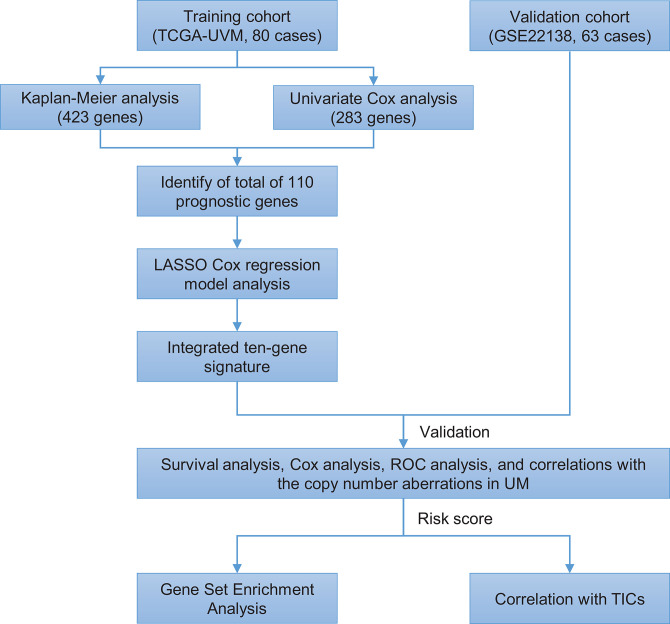
Brief flow chart of this study. The study was performed using TCGA-UVM and GSE22138 cohorts. The training cohort was applied to detect prognostic genes. LASSO regression model was for establishing a prognostic signature based on the prognostic genes. Then we validated the prognostic signature we established in the validation cohort. Finally, GSEA and TIC analysis were implemented to explore potential mechanisms further on the prognosis signature we found. LASSO, the least absolute shrinkage and selection operator Cox regression model; ROC, receiver operating characteristic; TICs, tumor-infiltrating immune cells; UM, uveal melanoma; GSEA, Gene Set Enrichment Analysis.

The flowchart of the present research is shown in Figure 1. Eighty UM cases that came from TCGA-UVM were taken as the training cohort. The dataset GSE22138 with 63 UM patients was used as the validation cohort. The detailed clinical characteristics of both cohorts were summarized in Table 1.

**Table 1:** Clinical characteristics of patients involved in the study.

**Characteristics**	**Training cohort (TCGA-UVM, *n* = 80)**	**Validation cohort (GSE22138, *n* = 63)**
**Age at diagnosis, years**		
<60	36 (45.00%)	28 (44.44%)
≥60	44 (55.00%)	35 (55.56%)
Unknown	0 (0.00%)	0 (0.00%)
**Gender**		
Female	35 (43.75%)	24 (38.10%)
Male	45 (56.25%)	39 (61.90%)
Unknown	0 (0.00%)	0 (0.00%)
**Stage**		
I	0 (0.00%)	NA
II	36 (45.00%)	NA
III	40 (50.00%)	NA
IV	4 (5.00%)	NA
Unknown	0 (0.00%)	NA
**T classification**		
T1	0 (0.00%)	NA
T2	4 (5.00%)	NA
T3	36 (45.00%)	NA
T4	38 (47.50%)	NA
Unknown	2 (2.50%)	NA
**N classification**		
N0	76 (95.00%)	NA
N1	0 (0.00%)	NA
Unknown	4 (5.00%)	NA
**M classification**		
M0	73 (91.25%)	28 (44.44%)
M1	3 (3.75%)	35 (55.56%)
Unknown	4 (5.00%)	0 (0.00%)
**Extrascleral extension**		
No	68 (85.00%)	48 (76.19%)
Yes	7 (8.75%)	5 (7.94%)
Unknown	5 (6.25%)	10 (15.87%)
**Tumor basal diameter, mm**		
<12	6 (7.50%)	11 (17.46%)
≥12	73 (91.25%)	42 (66.67%)
Unknown	1 (1.25%)	10 (15.87%)
**Tumor thickness**		
<8	15 (18.75%)	3 (4.76%)
≥8	65 (81.25%)	60 (95.24%)
Unknown	0 (0.00%)	0 (0.00%)
**Tumor side**		
Right	NA	30 (47.62%)
Left	NA	33 (52.38%)
Unknown	NA	0 (0.00%)
**Tumor location**		
On equator	NA	42 (66.67%)
Anterior to equator	NA	3 (4.76%)
Posterior to equator	NA	9 (14.29%)
Other	NA	4 (6.35%)
Unknown	NA	5 (7.94%)
**Retinal detachment**		
No	NA	22 (34.92%)
Yes	NA	36 (57.14%)
Unknown	NA	5 (7.94%)

### Construction of Prognostic Signature From Training Cohort

**Figure 2:**
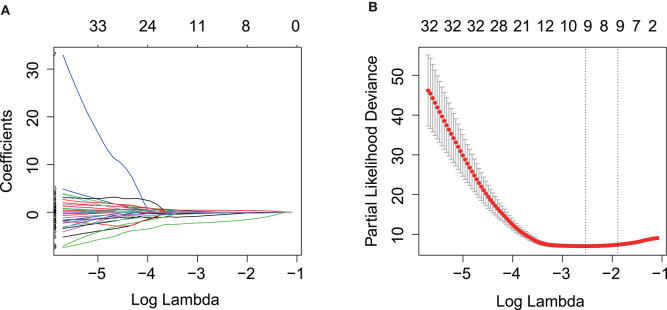
Prognostic gene signature was established by LASSO regression analysis. **(A)** LASSO coefficient profiles of the 110 genes in training cohort. **(B)** A coefficient profile plot was generated against the log (lambda) sequence. Selection of the optimal parameter (lambda) in the LASSO model for training cohort. LASSO, the least absolute shrinkage and selection operator Cox regression model.

Kaplan–Meier and univariate Cox regression analysis were performed on 80 patients in the training cohort to assess the prognostic relationship between gene expression profiles and overall survival, disease-specific survival, and progression-free survival. Four hundred and twenty-three genes were extracted from the Kaplan–Meier analysis (Supplementary Table 1), while, 283 genes were identified significant in the Cox regression analysis (Supplementary Table 2). Taking together, 110 genes in the intersection of the two results are defined as potential prognostic genes for next analyses (Supplementary Table 3). These genes were then subjected to LASSO Cox regression analysis, and regression coefficients were calculated. The coefficient of each gene was plotted in Figure 2A. The model achieved the best performance when it included 10 genes (Figure 2B). These genes, their corresponding coefficients, and genomic location were shown in Table 2.

**Table 2:** Genes in the prognostic gene signatures.

**Gene symbol**	**Full name**	**Risk coefficient**	**Genomic location (GRCh38/hg38)**
STPG1	Sperm Tail PG-Rich Repeat Containing 1	−0.150605911	chr1:24,356,999–24,416,934
HMCES	5-Hydroxymethylcytosine Binding, ES Cell Specific	−0.526265796	chr3:129,278,816–129,306,186
ANXA2P2	Annexin A2 Pseudogene 2	0.017480411	chr9:33,624,225–33,625,534
CA12	Carbonic Anhydrase 12	0.414736428	chr15:63,321,378–63,382,110
RNF208	Ring Finger Protein 208	−0.098017226	chr9:137,220,247–137,221,581
SLC44A3	Solute Carrier Family 44 Member 3	−0.175213008	chr1:94,820,342–94,895,247
TCTN1	Tectonic Family Member 1	−0.171507956	chr12:110,614,027–110,649,430
POMGNT2	Protein O-Linked Mannose N-Acetylglucosaminyltransferase 2 (Beta 1,4–)	−0.106148114	chr3:43,079,229–43,106,083
ULBP1	UL16 Binding Protein 1	0.037591702	chr6:149,963,943–149,973,715
SIRT3	Sirtuin 3	−2.002826257	chr11:215,030–236,931

### Prognostic Value of the Ten-Gene Signature in the Training and Validation Cohorts

According to the gene expression level, and the risk coefficient of each gene, the risk score of each patient was calculated. The median risk score was the cut-off value for assigning patients to high-risk or low-risk groups. The prognostic value of the risk score was evaluated by comparing the survival differences between the high-risk group and the low-risk group.

**Figure 3:**
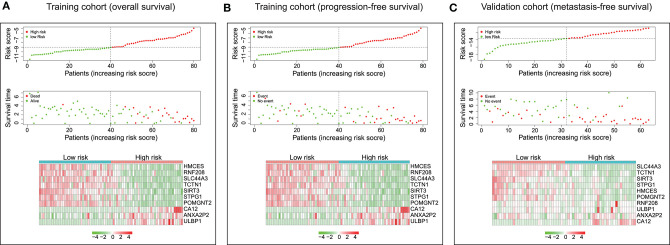
Characteristics of the ten-gene signature. (Upper and middle) The distribution of ten-gene risk score and patients' survival time, and events for training cohort based on overall survival **(A)**, training cohort based on progression-free survival **(B)**, and validation cohort based on metastasis-free survival **(C)**. According to the median risk score, patients were divided into low-risk and high-risk groups. The left side of the black dotted line is the low-risk group, and the right side is the high-risk group. (Bottom) Heatmaps were plotted to illustrate the ten-gene expression profiles in the training cohort based on overall survival **(A)**, training cohort based on progression-free survival **(B)**, and validation cohort based on metastasis-free survival **(C)**.

The distribution of risk scores and overall survival status and the expression profiles of the ten-gene signature of the patients in the training cohort were plotted in Figure 3A. As shown in the figure, there are more deceased in high-risk patients, and the survival time is shorter than that of low-risk patients. The heat map shows that SIRT3, HMCES, SLC44A3, TCTN1, STPG1, POMGNT2, and RNF208 were under expressed in high-risk patients, while, ANXA2P2, ULBP1, and CA12 were highly expressed in high-risk patients. In addition, we examined the performance of these ten-gene signature in predicting progression-free survival in the training cohort. As shown in Figure 3B, in the high-risk group, more events happened, and shorter survival time gained. The pattern did consistent with that in predicting overall survival. Furthermore, we checked the predictive power of this ten-gene signature for metastasis-free survival in the validation cohort. It could be seen that there were more metastasis events occurred in the high-risk group than in the low-risk group, and the survival time of the high-risk group was also shorter (Figure 3C).

**Figure 4:**
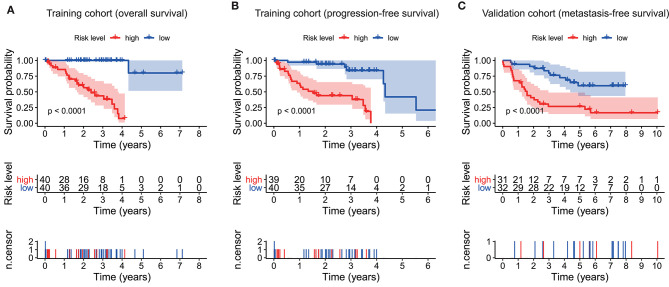
Kaplan–Meier survival analyses based on the ten-gene signature. **(A)** Training cohort based on overall survival. **(B)** Training cohort based on progression-free survival. **(C)** Validation cohort based on metastasis-free survival. Differences between curves were detected by two-side log-rank test.

As plotted in Figure 4A, Kaplan–Meier survival analysis in the training cohort showed that the overall survival of patients in the high-risk group was poorer than that in the low-risk group (*p* < 0.0001, Figure 4A). Also, an unfavorable progression-free survival was seen in the training cohort (*p* < 0.0001, Figure 4B). To further explore the efficacy of the ten-gene signature in predicting prognosis (metastasis-free survival) in UM patients, we tested the ten-gene signature in the validation cohort. Adopting the same classification method, patients were divided into high-risk and low-risk groups based on the median risk score. Consistent with previous results, patients in the high-risk group showed significantly worse metastasis-free survival than patients in the low-risk group (*p* < 0.0001, Figure 4C).

**Figure 5:**
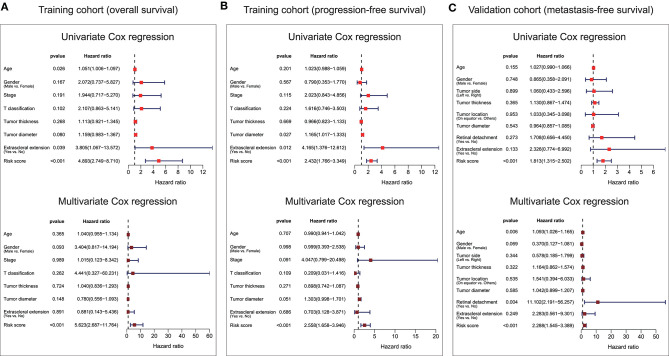
Forest plot summary of univariate and multivariate Cox analyses of prognosis. Univariate (upper) and multivariate (bottom) analyses were carried out using the ten-gene signature and clinical covariates in the training cohort based on overall survival **(A)**, training cohort based on progression-free survival **(B)**, and validation cohort based on metastasis-free survival **(C)**. Colored solid squares represent HR, and the horizontal line across the HR represents the 95% CI. All *p*-values were calculated using the Cox regression hazards analysis. HR, hazard ratio; 95% CI, 95% confidence interval.

Univariate and multivariate Cox analyses were conducted in the training cohort based overall survival and progression-free survival, and validation cohort based on metastasis-free survival, using the available co-variables including risk score, age, gender, T classification, tumor stage, tumor thickness, tumor diameter, tumor side, tumor location, extrascleral extension, or retinal detachment to detect whether our ten-gene signature had the prognostic capacity that was independent from the clinic-pathologic characteristics. In the training cohort, both univariate and multivariate Cox regression analyses indicated that the ten-gene signature was a powerful variable associated with overall survival (HR = 4.893, 95% CI = 2.749–8.710, *p* < 0.001, and HR = 5.623, 95% CI = 2.687–11.764, *p* < 0.001, respectively; Figure 5A), and progression-free survival (HR = 2.432, 95% CI = 1.766–3.349, *p* < 0.001, and HR = 2.558, 95% CI = 1.658–3.946, *p* < 0.001, respectively; Figure 5B). Consistent with that in the training cohort, the ten-gene signature displayed pronounced capability in the validation cohort in predicting metastasis-free survival (Figure 5C). These results proved that the ten-gene signature was to be a strong and independent variable.

**Figure 6:**
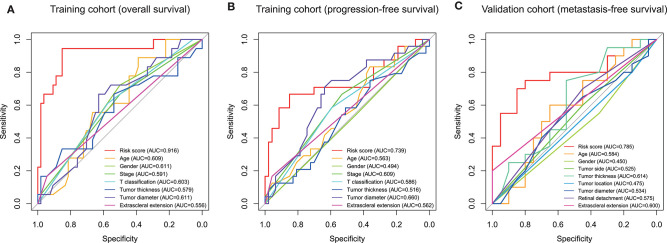
Receiver operating characteristic (ROC) analysis of the ten-gene signature risk score. ROC analysis of the sensitivity and specificity of the prognosis prediction by the ten-gene risk score, age, gender, T classification, tumor stage, tumor thickness, tumor diameter, tumor side, tumor location, extrascleral extension, or retinal detachment in training cohort based on overall survival **(A)**, training cohort based on progression-free survival **(B)**, and validation cohort based on metastasis-free survival **(C)**. AUC, area under the ROC curve.

Subsequently, we conducted ROC analyses to assess how the ten-gene signature could behave in predicting prognosis. As shown in Figure 6A, the area under the ROC curve (AUC) of the ten-gene risk score model performed on overall survival in the training cohort was 0.916, which was superior to those of age, gender, stage, T classification, tumor thickness, tumor diameter, and extrascleral extension (0.609, 0.611, 0.591, 0.603, 0.579, 0.611, and 0.556, respectively). Consistently, in the prediction model of progression-free survival predicted in the training cohort, the ten-gene signature risk score also showed a powerful ability with AUC = 0.739, which was far better than other variates (Figure 6B). This finding was also confirmed in validation cohort for metastasis-free survival predication (AUC = 0.785, Figure 6C).

**Figure 7:**
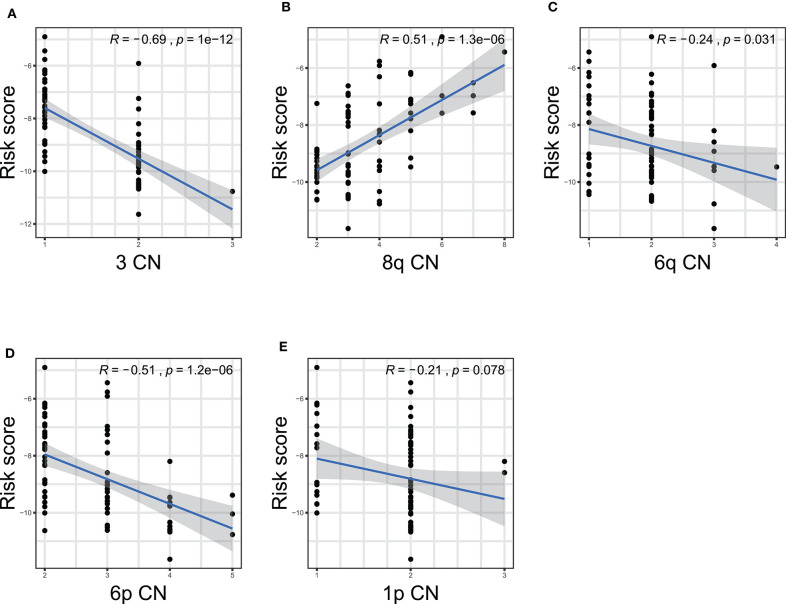
The correlations between the ten-gene signature and the copy number aberrations in TCGA-UVM. The correlations between risk score and chromosome 3 **(A)**, 8q **(B)**, 6q **(C)**, 6p **(D)**, and 1p **(E)** mutations were plotted. The blue line in each plot was fitted linear model indicating the proportion tropism of the copy number along with risk score. The shade around the blue line represents the 95% confidence interval. The Spearman test was applied for the correlation examination. CN, copy number.

Furthermore, we performed correlation analyses to assess the relationship between the ten-gene signature and status of chromosome copy number aberrations. The status of chromosome copy number aberrations of each patient in the TCGA-UVM cohort was downloaded from Robertson's publication (Supplementary Table 4) (16). Spearman test was used to assess the correlation between copy chromosome numbers and the risk score. The results showed that the ten-gene signature was significantly correlated with copy numbers of chromosome 3, 8q, 6q, and 6p (Figure 7). Specifically, the gene signature displayed negative correlations with the copy number of chromosome 3 (*R* = −0.69, *p* = 1e−12), 6q (*R* = −0.24, *p* = 0.031), and 6p (*R* = −0.51, *p* = 1.2e−06) (Figures 7A,C,D), while, showed positive correlation with chromosome 8q copy number (*R* = 0.51, *p* = 1.3e−06) (Figure 7B).

### Gene Set Enrichment Analysis With the Ten-Gene Signature

**Figure 8:**
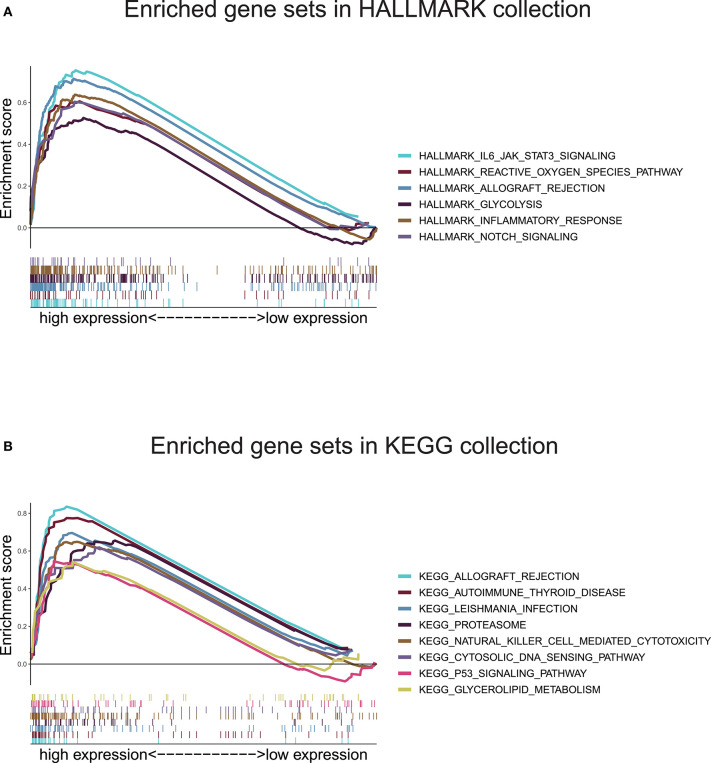
Gene set enrichment analysis based on the ten-gene signature. **(A)** Enriched gene sets annotated by the HALLMARK collection between the high and low-risk groups in the training cohort. **(B)** Enriched gene sets annotated by the KEGG collection between the high and low-risk groups in the training cohort. Gene sets with |NES |> 1, NOM *p* < 0.05, and FDR *q* < 0.25 were considered significant.

In view of the negative correlation between the level of the ten-gene signature risk score and the prognosis of UM patients, the GSEA was conducted between the high and the low-risk groups. As displayed in Figure 8A and Supplementary Table 5, all significantly enriched gene sets of HALLMARK collection were seen in the high-risk group in pathways relate to immune response, inflammatory response, reactive oxygen species, notch signaling, glycolysis, IL-6/JAK/STAT3 signaling, and allograft rejection. For HALLMARK collection defined by the Molecular Signatures Database, all gene sets were also enriched in the high-risk score group. These pathways were mostly associated with p53 signaling, autoimmune disease, proteasome, natural killer cell, cytosolic DNA-sensing, allograft rejection, leishmania infection, and glycolipid metabolism (Figure 8B and Supplementary Table 6). These findings indicated that the risk score was potentially closely related to the status of tumor microenvironment.

### Correlation of Risk Score With the Proportion of Tumor-Infiltrating Immune Cells (TICs)

**Figure 9:**
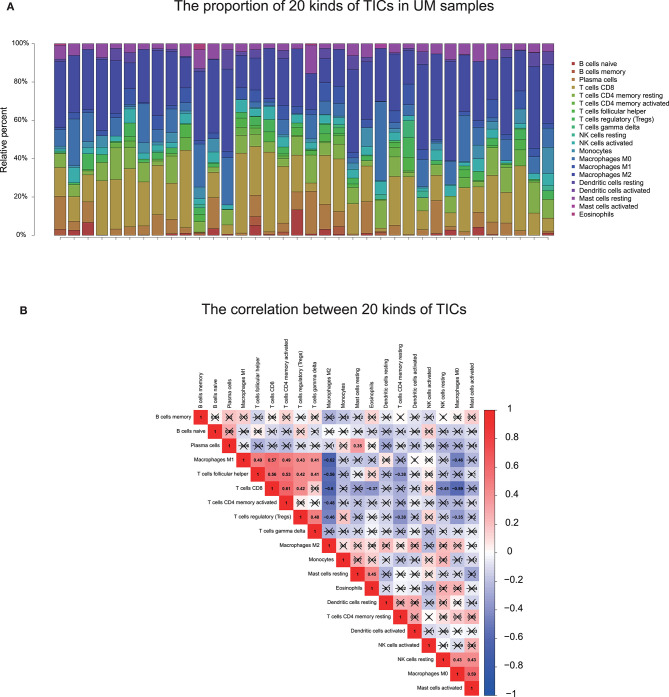
TIC profile and correlation analysis. **(A)** The bar graph showed the proportion of 20 TICs in UM tumor samples in the training cohort. Each column indicates one sample. **(B)** Heatmap showing the correlation between 20 kinds of TICs. The numeric and shade of each small color box indicate the coefficient between two kinds of cells. X shape covered coefficient is no statistically significant. The Pearson coefficient was used for the significance tests. *P* < 0.05 is the cutoff. TIC, tumor-infiltrating immune cell; UM, uveal melanoma.

**Figure 10:**
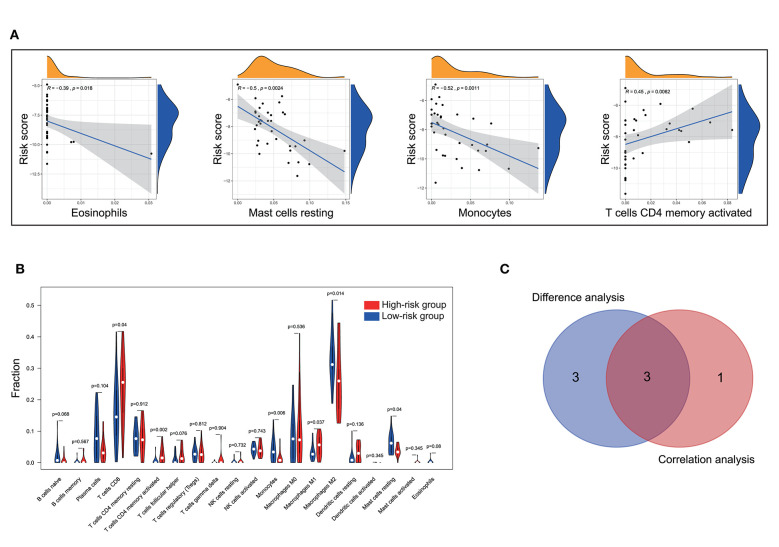
Correlation of TICs proportion with ten-gene signature risk score in the training cohort. **(A)** Only significantly correlated TICs was plotted. The blue line in each plot was fitted linear model indicating the proportion tropism of the immune cell along with risk score. The shade around the blue line represents the 95% confidence interval. The Spearman coefficient was used for the correlation test. **(B)** The violin plot showed the ratio differentiation of 20 kinds of immune cells between UM tumor samples with low and high-risk groups and was tested by Wilcoxon rank-sum. **(C)** The Venn plot displayed three kinds of TICs correlated with risk score co-determined by difference and correlation tests shown in violin and scatter plots, respectively. *P* < 0.05 is the cutoff. TIC, tumor-infiltrating immune cell; UM, uveal melanoma.

To further check the correlation between the risk score and the immune microenvironment, as shown in Figure 9, we used the CIBERSORT algorithm to analyze the proportion of tumor-infiltrating immune subpopulations and constructed 20 immune cell profiles in UM samples. Combining the results of correlation analysis (Figure 10A, Supplementary Table 7) and difference analysis (Figure 10B), a total of three TICs were associated with ten-gene signature risk score (Figure 10C). Among them, T cells CD4 memory activated was positively correlated with risk score, while, Monocytes and Mast cells resting were negatively correlated with risk score.

## Discussion

In the present study, we built an UM prognostic signature by comprehensively analyzing the TCGA and GEO. By investigating the relationship using Kaplan–Meier, univariate Cox analyses, and LASSO Cox regression model between the patients' prognosis and gene expression in the training cohort, we obtained a ten-gene signature that was pronounced related to outcome. By applying this signature in the training cohort, statistical significance was observed in univariate and multivariate Cox analysis, ROC analysis, and Kaplan–Meier curve between high-risk and low-risk groups. The prognostic ability of the ten-gene signature was also validated in the validation cohort, showing the broadness and effectiveness of the ten-gene signature in predicting UM prognosis. In addition, we found that the risk score was correlated with the copy number of chromosome 3 negatively, and chromosome 8q positively, which further indicates the significance of the signature we found. Then the GSEA and immune infiltration analyses showed that the ten-gene signature risk score might be immune-related and involved in the tumor microenvironment in UM patients. For research in gene-signature of UM, we are the first to apply chromosomal variation to perform validation of gene-signature reliability. Such work we have done aimed to guide future research in UM.

After we constructed the ten-gene signature, we firstly confirmed its capacity to distinguish the prognosis of patients effectively. As shown in Figure 3A, the high-risk zone not only counted more deaths, but also the patients in it presented a shorter survival time than that in the low-risk zone. Moreover, the heatmap indicated that each of these ten genes had a differential expression pattern between the low-risk and high-risk groups. Importantly, this ten-gene signature also owned pronounced performance in the training cohort for predicting progression-free survival (Figure 3B), and in the validation cohort for metastasis-free survival (Figure 3C).

In addition, we examined the prognostic value of the ten-gene signature by Kaplan–Meier analysis in the training cohort based on overall survival and progression-free survival, and in the validation cohort based on metastasis-free survival, finding its significantly predicting ability in UM patients (Figure 4). Furthermore, univariate and multivariate analyses were performed in the three cohorts to confirm that whether our ten-gene signature can be an independent from other variables in predicting UM outcome. As plotted in Figure 5, no matter in training cohort or validation cohort, no matter based on overall survival, progression-free survival, or metastasis-free survival, whether it is univariate or multivariate Cox regression analysis, the variable of risk score was always statistically significant. The results, here, verified the predictive ability of the risk score, and its independence.

To further assess the predictive power of this ten-gene signature, we performed ROC analysis. AUC can be used to check the accuracy and predictive ability of biomarkers in diagnostic tests (25). ROC analysis indicated that the AUC of the ten-gene signature stayed above 0.7 in these two cohorts, and superior to other variates (Figure 6). These ROC results again suggested that our signature might strengthen the predictive accuracy of prognosis in UM.

Our signature was composed of ten genes, which were SIRT3, HMCES, SLC44A3, TCTN1, STPG1, POMGNT2, RNF208, ANXA2P2, ULBP1, and CA12, respectively. In the signature model, ANXA2P2, ULBP1, CA12 were unfavorably genes for the outcome, whereas other genes presented protective function on the prognosis of UM patients. Pseudogenes are nonfunctional segments of DNA that resemble functional genes (26, 27). Previous studies have suggested that pseudogenes will only participate in regulatory roles (28). Recent studies have shown that most pseudogene breaks follow a certain pattern, and it is likely that the pseudogenes of this pattern can be repaired under certain conditions to restore function (27). ANXA2P2 is one of three pseudogenes of annexin A2 that have recently been shown to be aberrantly transcribed in hepatocellular carcinoma (HCC) cells (29). A recent report revealed that the expression of ANXA2P2 was up-regulated in HCC and promoted HCC to be an aggressive phenotype (29). ULBP1 is related to MHC class I molecules, but its gene maps outside the MHC locus (30, 31). It functions as a stress-induced ligand for NKG2D receptor (31). In UM, NKG2D expression was detected in primary tumor lesions, in which a large amount of NKG2D lymphocyte infiltration was also observed (32). Metastatic UM lesions lost MIC expression and are absent of NKG2D+ lymphocytes (33). A recent study demonstrated that soluble NKG2D ligand is a biomarker related to the clinical outcome of immune checkpoint blockade therapy in patients with metastatic melanoma (34). CA12 is a membrane-associated enzyme. CA12 is highly expressed in many human cancers and often indicates a poor prognosis, so it is a promising target for cancer treatment (35). Among the genes that we found to have prognostic protection, SIRT3, the major deacetylase in mitochondria, plays a crucial role in modulating oxygen reactive species (ROS) and limiting the oxidative damage in cellular components (36). In some types of cancer, SIRT3 functions as a tumoral promoter, since it keeps ROS levels under a certain threshold compatible with cell viability and proliferation. On the contrary, other studies describe SIRT3 as a tumoral suppressor, as SIRT3 could trigger cell death under stress conditions (36). HMCES is a critical component of the replication stress response, mainly upon base misincorporation (37). Deregulated APOBEC activity is the source of a variety of cancer mutagenesis (38). HMCES can respond to APOBEC-induced abasic sites, maintain genome stability, and promote replication extension; otherwise, replication will be slowed down by the participation of TLS polymerase (38). Therefore, HMCES plays a vital role in this tumorigenesis process (38). A lately study showed that SLC44A3 is different expressed between normal and UM (39), in addition, Li et al. (40) found it was found SLC44A3 were associated with better survival in UM and indicated their protective roles. Recent studies revealed that TCTN1 is widely up-regulated in various types of human cancer (41–44), and acts as an oncogene via promoting proliferation, migration, or inhibiting apoptosis. However, in a study conducted by Xue et al. (12), TCTN1 was found to be low expressed in high-risk patients with UM and has a protective effect on the prognosis of UM, which has been consistent with our study. STPG1 is found with few traces from existing studies, but shows to be a prognostic marker in endometrial cancer (favorable) and renal cancer (favorable) from The Human Protein Atlas portal (45). The high expression levels of human POMGNT2 in the brain, muscle, heart, and kidney in fetal as well as adult tissues suggest the importance of this gene during development (46). However, whether POMGNT2 plays a vital role in tumor progress remained unclear and needs more efforts in further research. RNF208 decreases the stability of soluble Vimentin protein through a polyubiquitin-mediated proteasomal degradation pathway, thereby suppressing metastasis of triple-negative breast cancer (TNBC) cells (47). In a comprehensive bioinformatics study, RNF208 was found to have decreased expression in UM and was associated with a better prognosis (12). There are relatively fewer studies related to these genes and UM. However, the ten-genes signature has a significant role in predicting and diagnosing UM in our research. The ten-gene signature or each of them may be the potential specific directions for future research on UM.

Studies showed that chromosome aberrations and gene mutations in UM are closely related to clinical results. The loss of a chromosome 3 in UM is associated with an increased risk of metastasis and poor prognosis (16). Recently, researchers also found that Monosomy 3 is associated with poor survival after UM treatment (19). Previous studies have shown that besides chromosome 3, the increase in chromosome 8q is also related to poor survival prognosis (48–51). In addition, other chromosomal abnormalities have been shown to correlate with poor prognosis and these include 6q loss, lack of 6p gain, 1p loss, and 16q loss (16–20). Among the ten gene signatures found in this study, five were located in the above-mentioned chromosomes (Table 2). Further on, we performed Spearman test to assess the correlation between the copy numbers of chromosome 3, 8q, 6q, 6p, and 1p and risk score, finding that the ten-gene signature risk score was significantly correlated with copy numbers of chromosome 3, 6q, and 6p negatively, and 8q positively (Figure 7), which further confirmed the crucial of the ten-gene signature in predicting prognosis of UM.

The GSEA found that gene sets enriched in pathways concerned with immune response, inflammatory response, p53 signaling, reactive oxygen species, Notch signaling, proteasome, natural killer cell, cytosolic DNA-sensing, and glycolipid metabolism. These findings demonstrated that ten-gene signature might potentially participate in the immune-dominant tumor microenvironment. The proportion of TICs analysis based on CIBERSORT algorithm found that activated T cells CD4 memory were positively correlated with risk score, while, Monocytes and Mast cells resting were negatively correlated with risk score, further supporting that the signature interacted closely with the tumor environment. Strategies targeting the tumor microenvironment of UM have the potential to improve the efficacy of standard and genome-based molecular therapeutics, and, as well, to help resolve many of the challenges associated with developing new drugs and running clinical trials (52). In our GSEA, KEGG collection indicated that NK cells were associated with the ten-gene risk score. This finding is consistent with previous research (53). Durante et al. (53) recent work identified LAG3 as a potential candidate for immune checkpoint blockade in patients with high risk UM, and demonstrated that LAG3 was expressed on NK cells, CD8+ T cells, and regulatory T cells, highlighting the vital of NK cells in UM. However, through immune cell and V(D)J immune repertoire analysis, Durante et al. (53) group found NK cells were few present, and they were distributed equally across tumor samples. This finding explains why NK cells stood out in GSEA but were not prominent in our CIBERSORT result. We thought the main reason was that the small amount of NK cells was “ignored” by the CIBERSORT algorithm, which led to the discrepancy of data analysis results. In Durante et al.'s (53) research, T cells were found present in all tumor samples and collaborated with LAG3 operating UM development. This conclusion was similar to our finding that the infiltration of CD4 T cells was correlated with the ten-gene risk score. Moreover, NK cells can recognize and directly kill early activated T cells, which can determine the quality and intensity of T cell responses, thereby affecting the immune process (54). As described above, although NK cells were “ignored” by the CIBERSORT algorithm, their ability in UM progress were not hidden, but be potentially “stolen” by T cells that are strictly related to it, further explained why NK cells appeared in our GSEA results but disappeared in the CIBERSORT conclusions.

The immune system uses multiple antigens to distinguish tumor cells from healthy cells (55). In many cancers, immune infiltration within the tumor is usually associated with a better prognosis and a favorable immunotherapy response (56). However, in primary UM, market-specific immunohistochemistry has demonstrated that dense infiltrate of leukocytes or macrophages is associated with monosomy 3 and a poor prognosis (57–59). UM cells express tumor-specific antigens, including the Melanoma Antigen Gene (MAGE) family proteins, premelanosome protein gp100, and tyrosinase (60, 61). But, both the innate and adaptive effector immune responses can be circumvented by UM cells (55), and previous studies have shown that UM cells have established a specific immune escape mechanism, leading to its progressive process and poor prognosis (55, 60–63). Contrary to other cancers, the increase in HLA class I expression is related to the poor prognosis of UM and is considered to be a mechanism by which natural killer cell-mediated cytotoxicity in the blood escapes tumors (64, 65). A recent study demonstrated that immune infiltration in UM is highly correlated with the upregulation of stimuli and targets (such as HLA and IFNG) that are fundamental for T cell-mediated immunotherapy (16). More recent reports suggest that disseminated conjunctival melanoma may be responsive to targeted molecular therapies, such as BRAF and MEK inhibitors in BRAF-mutant tumors (66), and checkpoint inhibitor immunotherapeutic agents, such as pembrolizumab (67). A better understanding of UM immunology can help select patients who may benefit from immunotherapy. However, the current knowledge of UM immunology is still in its infancy, and further research is needed to clarify the mechanism of UM inhibition and identify new targets to enhance anti-tumor immune reactivity.

DecisionDx-UM is a prognostic test that determines the metastatic risk associated with UM (68). Specifically, the assay determines the activity or “expression” of 15 genes which indicate a patient's individual risk, or class. The test classifies tumors as: Class 1 (low metastatic risk); Class 2 (high metastatic risk) (68). According to the report of the Collaborative Eye Oncology Group (COOG), the DecisionDx-UM GEP test is an accurate prospectively validated molecular classifier whose results are highly correlated with metastatic potential (69, 70). In a prospective multicenter study, Plasseraud et al. (71) demonstrated that the DecisionDecxD-UM could accurately predict the risk of metastasis in patients with UM. Compared with the seminal work of DecisionDx-UM, the present study obtained robust ten-gene signature by applying various statistical methods and validation in an independent cohort. Fewer gene numbers can save costs and improve efficiency in clinical practice. However, the results of the predecessors have been applied in commerce and have been widely reported and verified. In this regard, our research has great potential while still a long way to go.

Our research also has some limitations. Although TCGA-UVM is a cohort that is currently recognized by most scholars, the data in it are from large uveal melanoma treated with enucleation. Similarly, the GSE22138 cohort, which was published online on the GEO database platform, and its academic recognition is also undoubted. Still, most of the data in it came from large eye tumors. Such sample distribution in these two cohorts may not be consistent with the clinical population. Therefore, our research may have a selection bias for database selection. Our ten-gene signature came from retrospective data, and more prospective data were needed for proving the clinical utility of it. In addition, due to the limited clinical characteristics of patients included in TCGA cohort, we could not perform certain clinical subgroup analyses. Besides, there is currently no wet experimental data explaining the relationship between these ten-genes and their mechanism in UM samples. Therefore, between the ten-gene signature and the prognosis of UM, more effort is needed to clarify the potential relationship.

## Conclusion

In conclusion, our research defined a robust ten-gene signature in UM. It is a comprehensive analysis of the TCGA and the GEO database. This signature was related to the prognosis of UM and can accurately identify the prognostic risk of patients. Notably, we evaluated the reliability and accuracy of the signature by compared to variants of chromosomes 3 and 8q, and examining in a validation cohort. What is more, the functions and immune infiltrating analyses revealed that the signature had close interactions with the immunodominant tumor environment, which may advance the development of new therapies for UM treatment.

## Data Availability Statement

Publicly available datasets were analyzed in this study. These data can be found here: TCGA: https://portal.gdc.cancer.gov/; GEO: https://www.ncbi.nlm.nih.gov/geo/.

## Author Contributions

HL organized and wrote the manuscript. CM and JC contributed to the literature search for the manuscript. CM designed and produced the figures. JS made contributed to the statistical analysis of this manuscript. JC revised the manuscript. All authors reviewed the manuscript and approved the manuscript for publication.

## Conflict of Interest

The authors declare that the research was conducted in the absence of any commercial or financial relationships that could be construed as a potential conflict of interest.
