# Supplementary material for: Prognostic Implications of Novel Ten-Gene Signature in Uveal Melanoma
Source: Front Oncol. 2020 Oct 30;10:567512. doi: 10.3389/fonc.2020.567512 (PMC7661968; doi:10.3389/fonc.2020.567512)
Supplement: Supplementary file 1 [file Data_Sheet_1.PDF]

Table S1. Four hundred and twenty-three genes were significantly predicting prognosis of UM patients by Kaplan-Meier analysis (p-value < 0.0001).

Gene	Kaplan-Meier analysis (p-value)		
	Overall survival	Disease-specific survival	Progression-free survival
AC002401.4	3.79141E-05	3.92256E-05	1.71221E-05
AC002542.5	0	0	2.89206E-09
AC003092.1	8.28764E-06	2.68111E-06	9.84327E-09
AC004522.4	1.51971E-05	1.65297E-05	8.83127E-05
AC004847.1	4.1573E-05	4.65056E-05	5.28158E-05
AC006435.2	3.87307E-05	1.09866E-05	3.41481E-05
AC006458.1	0	0	2.89206E-09
AC008555.1	1.26274E-06	2.46964E-06	4.17792E-05
AC008555.2	2.29811E-07	4.48587E-07	4.28367E-05
AC008555.4	7.79302E-08	4.94966E-09	1.99685E-06
AC008892.1	2.69384E-07	2.12622E-07	5.24655E-05
AC010273.3	1.31844E-05	1.574E-06	5.85145E-06
AC010501.1	0	0	2.89206E-09
AC010619.2	1.14331E-06	1.14331E-06	2.0417E-13
AC011979.2	1.52666E-09	1.52666E-09	3.96482E-05
AC016747.1	2.92813E-05	2.99366E-05	1.78204E-05
AC016876.1	3.81782E-09	3.81782E-09	6.76648E-05
AC016924.1	2.49543E-05	6.72744E-06	6.78138E-06
AC017015.1	0	0	2.89206E-09
AC018529.1	7.99098E-07	7.10228E-07	1.03762E-06
AC020928.1	3.93935E-07	8.08424E-07	1.02133E-05
AC021613.2	3.81782E-09	3.81782E-09	6.76648E-05
AC022007.1	3.06343E-05	3.28808E-05	3.75516E-05
AC022784.1	6.54425E-06	5.79397E-06	5.17629E-06
AC022832.1	2.41312E-06	2.11773E-06	8.11737E-05
AC023511.2	0	0	2.89206E-09
AC027117.2	5.165E-05	9.47826E-05	1.1824E-07
AC040970.1	3.03026E-05	3.24277E-05	7.21754E-06
AC060834.2	0	0	2.89206E-09
AC078785.1	2.59968E-05	4.62963E-05	7.39019E-05
AC083805.1	7.84037E-05	2.41118E-05	1.78464E-05
AC087386.1	3.81782E-09	3.81782E-09	6.76648E-05
AC087883.1	1.84883E-09	1.84883E-09	1.11516E-06
AC090193.1	1.84883E-09	1.84883E-09	1.11516E-06
AC090821.4	0	0	2.89206E-09
AC092818.1	7.37759E-06	8.08482E-07	2.56141E-06
AC092821.2	1.12362E-08	8.4835E-09	3.78169E-06
AC092910.2	0	0	2.89206E-09
AC096536.1	3.81782E-09	3.81782E-09	6.76648E-05
AC098934.1	3.15552E-05	6.84356E-05	6.98908E-05
AC104117.3	5.12137E-06	1.0456E-05	5.36004E-05
AC104129.1	2.51321E-06	2.31727E-06	2.97215E-05
AC107391.1	0	0	2.89206E-09

AC112250.1	3.42631E-06	2.07092E-06	6.26726E-07
AC114752.1	0	0	2.89206E-09
AC116348.2	3.81782E-09	3.81782E-09	6.76648E-05
AC117422.1	2.30007E-06	2.15316E-06	4.65873E-05
AC117834.2	1.52666E-09	1.52666E-09	3.96482E-05
AC141557.1	1.12362E-08	8.4835E-09	4.61251E-06
AC239800.3	3.81782E-09	3.81782E-09	6.76648E-05
AC241377.1	1.11022E-16	1.11022E-16	2.89206E-09
ACAD10	2.43505E-05	2.40242E-05	2.22849E-05
ACADVL	5.79189E-06	5.57066E-06	6.37501E-05
ACKR2	8.24868E-07	7.3416E-07	4.2159E-07
ACSF2	1.2972E-06	1.28132E-06	1.93433E-05
ACVR2B-AS1	1.54837E-06	1.42157E-06	1.96482E-06
ADAM11	3.92458E-06	4.22335E-06	2.39906E-06
ADCK5	1.38931E-05	1.50443E-05	7.23578E-06
ADGRG1	6.25929E-05	6.19752E-05	5.33099E-06
AHNAK2	5.48988E-05	6.02711E-05	6.05973E-05
AIFM2	6.03304E-06	3.36675E-06	3.52585E-05
AK4	6.98268E-05	7.73374E-05	4.69104E-05
AL121672.2	6.65311E-05	4.42439E-05	2.19908E-05
AL137071.1	1.46216E-13	1.46216E-13	6.76648E-05
AL139390.1	0	0	2.89206E-09
AL356432.3	3.81782E-09	3.81782E-09	6.76648E-05
AL356961.1	5.44251E-05	1.58128E-05	2.08923E-06
AL358972.1	0	0	2.89206E-09
AL391422.4	1.00365E-05	1.08563E-06	9.55165E-05
AL391987.3	0	0	2.89206E-09
AL451074.2	1.10073E-05	2.21916E-05	9.27768E-05
AL512286.1	3.06252E-05	3.06252E-05	2.03992E-05
AL513043.1	0	0	2.89206E-09
AL606469.1	3.96522E-05	7.57024E-05	5.75531E-05
ALG1L2	1.75836E-06	1.62479E-06	4.0546E-06
AMN	9.20972E-05	6.28326E-05	2.94334E-05
AMZ1	9.37784E-07	8.40167E-07	9.23788E-07
ANG	5.73374E-05	8.44382E-06	1.64814E-06
ANKRD20A10P	9.76175E-06	1.06933E-06	8.08108E-06
ANKRD33B	1.58595E-07	1.35533E-07	1.61951E-05
ANKRD34A	2.29812E-06	2.33521E-06	1.91315E-06
ANO4	2.02756E-05	5.94323E-05	4.02461E-05
ANPEP	3.46581E-05	2.08483E-05	4.48143E-05
ANXA2P2	5.37849E-06	6.04906E-06	2.02506E-05
AP000533.1	7.67556E-05	4.71039E-05	8.85598E-05
AP000705.1	0	0	2.89206E-09
AP001007.1	7.03086E-05	7.24971E-05	8.85143E-06
AP001042.2	0	0	2.89206E-09
AP001172.2	0	0	2.89206E-09
AP001525.1	3.81782E-09	3.81782E-09	6.76648E-05
AP003497.1	2.98767E-08	2.98767E-08	4.78073E-06

ARMC9	1.87936E-06	1.7964E-06	1.35557E-05
ASB9	1.54448E-06	6.65344E-06	7.08427E-06
ATP5MC2P3	2.03633E-05	2.70197E-06	4.18851E-07
ATP8B3	5.99075E-05	9.14712E-06	5.03723E-05
AZGP1	1.4636E-05	1.41176E-05	1.9977E-05
BAP1	1.40315E-06	1.28324E-06	2.32414E-05
BATF3	8.16694E-06	8.363E-06	1.48206E-06
BAX	4.27425E-06	3.86682E-06	4.08936E-05
BCAT1	1.16217E-05	1.25075E-05	4.73164E-05
BICDL1	1.29042E-06	1.20955E-06	9.29154E-06
BRK1	5.84841E-05	6.18345E-05	1.85607E-05
BRPF1	5.47142E-09	1.18049E-08	1.92301E-05
BRSK1	6.96732E-05	4.63953E-05	1.01157E-06
BTBD6	6.88407E-09	1.69363E-08	1.50016E-05
BX323845.1	0	0	2.89206E-09
C14orf119	8.26101E-05	8.52934E-05	4.76021E-05
C17orf100	1.44903E-06	2.95775E-06	1.03456E-05
C4orf17	0	0	2.03943E-06
CA12	1.55048E-06	8.03226E-07	2.287E-06
CADM1	2.01628E-05	1.18959E-05	2.72294E-06
CALHM2	3.5304E-07	3.34105E-07	2.96011E-07
CAMK1	2.4783E-05	1.4885E-05	6.46984E-05
CAMSAP3	2.06594E-06	4.02545E-06	2.78571E-06
CARD11	5.30339E-08	4.53672E-08	6.24998E-07
CCDC188	6.72807E-05	2.97003E-05	2.18454E-05
CDCA7L	2.91938E-07	2.44845E-07	9.5946E-05
CDH24	1.27083E-06	1.18966E-06	6.97671E-06
CDK4P1	3.81782E-09	3.81782E-09	6.76648E-05
CDKN2AIPNL	1.81259E-05	7.43364E-05	4.89716E-05
CEBPA-DT	3.04684E-06	2.98741E-06	7.20345E-07
CELF2	7.25127E-06	4.30853E-06	7.48709E-07
CELF5	1.63469E-06	3.49371E-06	4.42972E-06
CENPX	1.51908E-06	3.2662E-06	1.25695E-05
CFAP58-DT	2.76906E-06	5.38713E-06	7.26257E-06
CHAC1	5.74193E-07	4.99772E-07	7.76341E-07
CHPT1	4.33774E-05	9.34199E-05	2.41994E-05
CICP6	0	0	2.89206E-09
CIDEC	1.10809E-06	2.20845E-07	1.27968E-05
CIDECP1	9.31251E-06	2.5253E-06	9.7651E-07
CITED1	5.77436E-05	6.1011E-05	8.05882E-05
CLCNKB	1.58491E-05	1.90014E-06	8.57527E-05
CLEC11A	6.5831E-06	1.39326E-05	1.32234E-05
CLYBL	3.22684E-06	3.01559E-06	6.38363E-06
CNTNAP1	4.36949E-05	4.54931E-05	4.61646E-05
COL9A3	3.00195E-05	3.11492E-05	2.6158E-05
COQ6	6.33066E-06	3.46345E-06	9.65489E-06
COX14	8.52343E-06	8.6215E-06	1.1311E-05
COX6A2	1.67136E-05	1.62221E-05	9.79923E-06

COX6C	4.1074E-07	8.87026E-07	1.2856E-05
COX6CP5	0	0	2.89206E-09
CPVL	9.19013E-06	8.8367E-06	4.36106E-05
CRELD2	2.27398E-05	1.44557E-05	3.10059E-06
CRHR1	8.26114E-05	8.84922E-05	8.7379E-06
CRY2	7.25506E-06	1.40856E-05	6.06978E-05
CTF1	3.47162E-05	3.74352E-05	4.66598E-05
CTNNBIP1	5.62886E-05	5.83725E-05	8.67676E-07
CYTOR	8.57349E-06	3.45601E-05	6.23247E-05
DERL1	2.92187E-06	2.84996E-06	1.21718E-05
DHRS7B	1.59664E-06	1.46777E-06	2.72043E-05
DLL4	5.00509E-07	4.57287E-07	5.13596E-07
DPY19L4P2	0	0	2.89206E-09
DTYMK	6.08075E-06	2.79537E-05	1.42714E-05
EFCAB12	1.41388E-05	2.7306E-05	2.36275E-05
EFS	9.45965E-08	2.05164E-07	1.24804E-05
EI24P3	0	0	2.89206E-09
EIF1B	3.84694E-06	7.47182E-06	6.08743E-06
ENPP2	1.3071E-06	1.18991E-06	2.51444E-06
ENTPD3-AS1	1.11967E-06	2.28905E-06	8.50913E-05
FABP3	3.28261E-06	3.12323E-06	2.93812E-06
FABP5	2.45994E-06	2.38009E-06	1.03265E-05
FAM86DP	4.78599E-05	3.02929E-05	3.02794E-06
FAM86HP	6.07145E-07	5.31412E-07	2.32423E-05
FAR1P1	0	0	2.89206E-09
FBXO17	5.00509E-07	4.57287E-07	3.40933E-06
FBXO36-IT1	0	0	2.30532E-11
FEZ1	2.77871E-05	1.79668E-05	2.92681E-06
FKBP10	2.78285E-06	2.57396E-06	3.55938E-05
FKBP11	3.15723E-08	1.56312E-07	1.98036E-07
FOXRED1	1.96482E-05	2.12554E-05	1.15951E-05
G6PC3	5.32394E-06	1.08278E-05	2.70988E-05
GABARAPL1	2.93974E-05	7.65803E-06	6.59995E-05
GATA4	2.74212E-06	7.75913E-06	2.54539E-05
GDF11	9.33452E-06	9.63086E-06	6.30594E-07
GEM	5.64978E-06	3.2929E-06	6.38637E-07
GLA	8.34545E-07	7.63463E-07	2.93763E-05
GLI2	5.86327E-06	5.92453E-06	4.86049E-06
GLIS2	1.98471E-05	2.18421E-05	1.58489E-05
GLRX2	8.53896E-05	9.5255E-05	2.46214E-05
GLUD1P2	4.30602E-05	8.9875E-05	1.32305E-05
GOLGA2P11	4.46561E-05	2.21775E-05	1.70664E-05
GPAA1P1	1.34255E-07	1.34255E-07	5.53804E-05
GPR156	3.29213E-06	6.68179E-06	6.14181E-06
GPR162	4.59317E-06	4.58812E-06	3.52475E-06
GPR27	9.61764E-05	9.62686E-05	5.66163E-05
GPR62	8.31733E-06	1.75382E-05	7.49252E-06
GREB1	3.24559E-05	3.20467E-05	3.0917E-05

GSTA3	9.91347E-07	4.60567E-07	4.84843E-06
GUSB	1.54565E-05	1.65246E-05	8.18847E-06
HACD1	3.19438E-05	1.95751E-05	3.51651E-05
HDAC11	3.1133E-06	3.05715E-06	7.3848E-06
HM13	4.06989E-06	3.66898E-06	5.44794E-05
HMCES	1.16729E-08	2.68654E-08	6.59499E-05
HOTAIRM1	4.42837E-06	2.37843E-06	7.44948E-06
HPSE	2.3184E-05	1.34117E-05	5.31255E-05
HS3ST3B1	5.94844E-05	3.97392E-05	6.63217E-05
HSFY1P1	3.81782E-09	3.81782E-09	6.76648E-05
HTRA1	4.96952E-06	2.7305E-06	9.07709E-07
HTRA3	1.59045E-06	1.50497E-06	2.17282E-06
HYKK	3.95238E-05	2.36399E-05	7.14696E-05
IDH2	3.94135E-05	4.08558E-05	6.12997E-05
IFT122	1.93891E-07	3.97769E-07	1.22603E-05
IGLV11-55	0	0	2.89206E-09
IL12RB2	3.34704E-05	4.49019E-06	8.28353E-05
IQSEC1	1.01495E-06	1.98183E-06	4.0839E-05
ISG20	3.08101E-07	2.64687E-07	1.99013E-05
ISM1	4.52659E-06	2.46168E-06	5.9253E-07
JAG2	9.07968E-06	1.14491E-06	1.17166E-05
JPH1	7.47602E-05	1.19121E-05	7.69843E-06
JUP	6.44106E-07	1.31927E-06	6.16642E-05
KCNQ2	1.30348E-05	1.30509E-05	1.47641E-05
KCTD17	4.2681E-06	4.25012E-06	4.38985E-06
KDELR3	5.84681E-08	5.5396E-08	2.27676E-07
KDM7A-DT	8.73862E-06	8.23607E-06	9.96183E-06
KIF9	1.42417E-05	2.86247E-05	2.42999E-05
KIRREL1	5.93457E-05	6.08151E-05	4.88885E-06
LAMA1	1.96921E-05	1.17723E-05	2.89355E-05
LEKR1	3.58429E-05	7.44062E-05	6.35033E-05
LHB	6.44178E-06	1.37599E-05	5.17197E-05
LHFPL2	4.81872E-05	9.56646E-05	1.84726E-05
LHFPL6	6.44431E-06	3.66779E-06	5.97788E-06
LINC00518	9.5023E-06	1.0963E-06	4.16616E-06
LINC00963	1.13621E-06	1.14392E-06	3.58252E-05
LINC01209	0	0	2.89206E-09
LINC01270	2.28959E-06	2.25945E-06	1.82496E-05
LINC01271	1.03993E-05	3.06704E-05	4.9275E-06
LINC01520	0	0	2.89206E-09
LINC01762	3.30098E-05	2.02641E-05	1.06212E-06
LINC02021	1.81187E-05	2.20165E-06	4.93238E-06
LINC02367	2.81232E-08	2.14898E-08	4.61251E-06
LINC02699	0	0	2.89206E-09
LINC02737	0	0	2.89206E-09
LINC02761	4.70396E-05	4.60448E-05	3.33063E-05
LINGO1	3.7063E-05	3.67578E-05	5.92837E-05
LMCD1	1.96384E-08	3.80705E-08	3.36323E-06

LNP1	6.55043E-07	1.40976E-06	1.16007E-05
LTBR	7.87352E-08	1.80851E-07	6.79528E-06
MAGEB6	3.81782E-09	3.81782E-09	6.76648E-05
MAGI2-AS2	3.81782E-09	3.81782E-09	6.76648E-05
MALSU1	4.04806E-05	4.39392E-05	1.01792E-05
MAN1B1-DT	2.51185E-06	5.68026E-06	3.14154E-05
MANEAL	2.12535E-08	4.88443E-08	5.54137E-06
MAP1A	2.10899E-05	2.12542E-05	1.17273E-05
MAP4K3-DT	6.53445E-06	6.85397E-06	1.88346E-05
MAPK12	6.22319E-07	1.40976E-06	4.10909E-06
MCCC2	3.44042E-05	9.12674E-06	4.53025E-05
MEGF10	1.53711E-06	3.13747E-06	1.57317E-05
MIB2	4.03004E-06	3.81452E-06	1.35346E-09
MIR375	3.81782E-09	3.81782E-09	6.76648E-05
MIR4460	0	0	2.89206E-09
MIR613	1.84883E-09	1.84883E-09	1.11516E-06
MIR635	1.68804E-05	1.68674E-05	5.32122E-05
MIRLET7BHG	8.10319E-05	4.82393E-05	1.13047E-05
MLIP	1.69731E-06	1.61352E-06	7.62078E-05
MMP11	5.74419E-05	5.821E-05	4.7813E-05
MPZ	3.42386E-06	3.47786E-06	3.06451E-05
MRPS6	5.29071E-05	5.35763E-05	7.6113E-05
MTATP6P17	6.34528E-05	3.82312E-05	1.49477E-05
MTCL1	4.12125E-06	4.68353E-06	1.1189E-05
MTHFD2P1	3.89501E-08	1.65622E-08	2.5683E-08
MTMR14	3.99571E-06	3.97081E-06	1.21905E-05
MTND6P10	0	0	2.89206E-09
MTUS1	8.3039E-06	1.54171E-05	9.69316E-05
MYEOV	3.49545E-05	3.46366E-05	3.85719E-05
MYL6P4	0	0	2.89206E-09
NCKAP5	3.76645E-05	7.19523E-05	9.77702E-05
NCKIPSD	1.76929E-05	1.84915E-05	1.3228E-05
NCS1	4.86474E-05	5.31497E-05	4.33164E-06
NDN	6.43404E-06	1.43561E-05	7.23622E-06
NDUFA9	1.3081E-06	1.19204E-06	1.59514E-06
NDUFB1	1.78028E-05	3.93703E-05	5.08225E-05
NFIA	5.83447E-06	1.23691E-05	1.31032E-06
NICN1	3.24324E-07	2.89606E-07	5.42443E-06
NIPA2P1	4.42043E-11	4.42043E-11	4.44247E-05
NKX3-2	4.85557E-06	4.75471E-07	5.36416E-05
NKX6-1	1.0833E-06	1.00628E-06	1.26726E-06
NMS	0	0	2.89206E-09
NQO1	1.21525E-05	1.21306E-05	9.57862E-06
NR2F1-AS1	2.95301E-06	8.73812E-06	1.54017E-05
NR6A1	5.52478E-07	1.19042E-06	1.05589E-05
NTNG2	2.6504E-05	2.82475E-05	9.79711E-05
OLIG1	2.45455E-05	2.3301E-05	3.7627E-05
OR4F13P	4.30096E-05	7.39707E-06	3.84086E-06

OR51K1P	3.81782E-09	3.81782E-09	6.76648E-05
OR5BN2P	0	0	2.89206E-09
OR7A18P	0	0	2.89206E-09
ORAI2	2.29245E-06	1.30271E-06	4.99827E-05
ORMDL2	6.70856E-08	1.45531E-07	1.20085E-05
OSBP2	5.61537E-06	1.13425E-05	9.32927E-06
OSBPL9P1	1.84883E-09	1.84883E-09	1.11516E-06
P2RX4	6.44115E-08	6.24478E-07	1.3324E-07
P2RX6	3.51275E-06	3.19672E-06	4.01747E-05
P4HA2	1.08033E-05	1.05455E-05	8.62634E-05
PACSIN3	6.36936E-05	9.11656E-06	6.52444E-05
PALM3	3.73156E-07	3.08286E-07	2.45212E-05
PCK1	1.40216E-05	5.53628E-05	2.12875E-06
PDXK	4.57083E-06	4.56895E-06	3.12705E-05
PFKP	3.87376E-07	3.68488E-07	3.99618E-07
PLAAT1	3.12278E-05	6.51169E-05	7.75989E-05
PLCD1	4.94688E-06	4.9641E-06	1.10003E-05
PLEKHG4B	8.78296E-06	5.56309E-06	1.35151E-07
PLEKHG6	7.31834E-05	8.04264E-05	3.3827E-05
PLXNB1	1.22158E-06	1.10923E-06	3.40621E-06
PMM2	9.32405E-06	8.76737E-06	2.69259E-05
POMGNT2	9.84951E-07	2.11412E-06	1.29919E-05
POMP	1.37201E-06	1.31936E-06	8.70432E-05
PPM1K	9.01414E-05	5.90266E-05	2.58623E-05
PRDX2P2	4.46181E-11	4.46181E-11	4.82386E-06
PRKCD	1.2651E-06	1.15009E-06	2.37191E-05
PRR5L	3.48981E-05	2.20168E-05	6.47216E-06
PRRT1	1.48021E-05	3.87478E-06	2.58379E-05
PSMB3	6.89488E-06	7.01052E-06	9.4054E-05
RAB31	3.63771E-05	4.04724E-05	9.32682E-05
RAPGEF3	2.69418E-05	2.87217E-05	7.28645E-06
RBM15B	1.10103E-07	2.25949E-07	2.02338E-05
RBM48P1	0	0	2.89206E-09
RBP7	5.98991E-05	3.69388E-05	7.75629E-06
RHOF	1.35505E-05	8.07849E-06	2.45529E-07
RN7SL187P	5.3338E-05	3.16739E-05	2.68948E-05
RN7SL241P	0	0	2.89206E-09
RN7SL794P	1.52666E-09	1.52666E-09	3.96482E-05
RNA5SP326	0	0	2.89206E-09
RNA5SP354	1.52666E-09	1.52666E-09	3.96482E-05
RNF208	2.48609E-06	5.05941E-06	2.31401E-05
RNU1-101P	3.81782E-09	3.81782E-09	6.76648E-05
RNU1-154P	0	0	2.89206E-09
RNU6-1265P	0	0	2.89206E-09
RNU6-1318P	0	0	2.89206E-09
RNU6-342P	0	0	2.89206E-09
RNU6-425P	3.81782E-09	3.81782E-09	6.76648E-05
RNU6-727P	3.81782E-09	3.81782E-09	6.76648E-05

RPL14	4.31483E-05	4.505E-05	9.39231E-05
RPL32	4.31483E-05	4.505E-05	1.56127E-05
RPL35A	1.2995E-05	1.28201E-05	6.10389E-05
RPL36P19	0	0	2.89206E-09
RRM2	3.79462E-06	2.93847E-05	4.64713E-06
SAP30	2.72654E-05	2.91249E-05	2.84446E-05
SATB1	4.6636E-06	8.68213E-06	8.80274E-05
SCD5	3.6181E-05	1.58906E-05	5.22192E-05
SCN1B	3.94764E-05	4.10678E-05	2.88861E-05
SCN4B	7.12557E-06	3.83978E-06	1.16989E-05
SDHA	2.59406E-05	2.67555E-05	2.89241E-05
SEL1L3	3.70416E-05	3.84432E-05	2.03925E-05
SEM1	1.23254E-05	8.18001E-05	8.41712E-05
SFXN3	5.0624E-06	5.50825E-06	2.11683E-06
SFXN5	1.24217E-05	1.40531E-05	7.26051E-06
SGK1	6.9973E-05	7.45579E-05	4.98616E-05
SGSM2	2.74563E-06	5.83826E-06	2.23643E-05
SH3PXD2A	3.67241E-05	2.27185E-05	1.36848E-07
SIRT3	1.92613E-06	3.77071E-07	3.56333E-05
SLC1A1	8.0637E-06	2.60093E-05	1.47548E-05
SLC22A31	5.66222E-05	5.9649E-05	1.12389E-05
SLC25A26	4.86394E-06	4.8727E-06	1.18861E-05
SLC25A38	5.32199E-07	4.87755E-07	1.20319E-06
SLC38A8	1.80027E-06	1.85653E-06	6.85384E-05
SLC41A3	7.41212E-07	1.59408E-06	8.60188E-06
SLC44A3	6.342E-07	5.53252E-07	5.96738E-05
SLC44A3-AS1	1.50032E-06	7.61193E-07	4.93288E-07
SLC45A2	7.08406E-07	1.71591E-06	9.75193E-05
SLC46A1	2.92672E-07	2.82805E-07	6.64835E-06
SLCO3A1	6.75834E-07	6.46153E-07	3.16903E-06
SMARCD3	4.81531E-06	4.81766E-06	5.80594E-07
SMIM10L2A	5.37578E-07	1.10248E-06	3.26355E-06
SMIM10L2B	3.0715E-07	6.63854E-07	1.4506E-06
SNCAIP	4.96918E-06	2.53717E-06	9.11487E-07
SNRPG	4.50672E-06	4.50223E-06	3.42973E-06
SOBP	1.62672E-09	3.28154E-09	1.77893E-05
SOCS2	4.24166E-06	4.20633E-06	2.49275E-05
SPAG8	1.22875E-05	2.71824E-05	8.21472E-05
SPDYE17	9.61166E-06	5.13337E-06	9.83289E-05
SPEF1	2.30508E-06	2.23205E-06	5.37491E-07
SRGAP2	4.64863E-05	3.00128E-05	6.79059E-06
SRGAP3-AS1	0	0	2.89206E-09
SRRT	7.86588E-06	1.00779E-06	5.18622E-07
SSUH2	1.02139E-05	1.09359E-05	1.92657E-05
SSX5	2.8532E-07	2.381E-07	6.81431E-06
ST8SIA2	1.65763E-06	1.52739E-06	1.53926E-07
STPG1	1.79625E-05	4.45702E-06	7.91245E-06
SULF2	9.53762E-08	7.93705E-08	1.1886E-05

SUPT20HL2	3.06252E-05	3.06252E-05	2.03992E-05
TAPBPL	2.39177E-07	2.1418E-07	2.3418E-05
TAS2R62P	0	0	2.89206E-09
TATDN2	1.31794E-06	1.16928E-07	3.20894E-06
TCTN1	3.25875E-10	6.924E-10	2.06358E-06
TFAP2A	3.86901E-05	4.19081E-05	4.69462E-05
TFAP2A-AS1	5.87887E-05	9.09888E-06	1.75116E-06
TIMM23	3.43729E-05	3.70598E-05	9.23171E-05
TMEM8B	1.04763E-05	2.12511E-05	8.17907E-05
TNFRSF18	1.15819E-05	2.24268E-05	9.24486E-05
TNFRSF21	2.15306E-05	2.17459E-05	1.2745E-05
TPPP	1.45344E-05	1.46216E-05	5.6388E-07
TRABD2B	5.28315E-06	5.75015E-07	5.0965E-06
TRPM4	2.21431E-06	1.04606E-05	1.92902E-05
TSC22D1	2.20902E-05	1.33435E-05	2.0452E-05
TSGA10IP	3.84762E-05	3.99606E-05	2.03041E-05
TUSC1	7.88759E-08	1.7106E-07	9.24332E-05
TUSC2	1.33165E-05	2.67861E-05	3.55206E-05
UBXN11	3.96056E-05	1.13575E-05	5.19476E-05
UGT1A12P	0	0	2.89206E-09
UGT1A3	0	0	2.89206E-09
UGT8	3.99147E-06	2.37152E-06	2.38408E-09
ULBP1	6.33443E-06	6.61799E-06	2.89419E-05
VAV2	4.43759E-05	5.03367E-05	3.89347E-05
VDAC1	7.12141E-06	7.26469E-06	6.18205E-06
VOPP1	1.05258E-06	9.76031E-07	1.3102E-05
WIPI1	1.01787E-06	9.41166E-07	3.9347E-06
WNT10B	1.23538E-06	3.09961E-06	4.18786E-06
YBX1P9	0	0	2.89206E-09
Z82246.1	1.38003E-05	9.50341E-07	8.04898E-07
Z93929.2	0	0	2.89206E-09
ZBTB44-DT	4.27064E-05	4.45428E-05	4.19739E-05
ZHX3	1.27406E-07	1.07689E-07	1.73974E-05
ZIM2-AS1	1.53185E-07	3.52088E-07	3.19594E-06
ZNF204P	7.23987E-07	1.56443E-06	1.27235E-06
ZNF391	1.18077E-05	1.12882E-05	2.93714E-05
ZNF454	1.20978E-05	2.45022E-05	2.08941E-05
ZNF467	6.32546E-07	6.02678E-07	5.53489E-06
ZNF497	1.40279E-06	3.01652E-06	3.3527E-05
ZNF576	7.96863E-07	4.04688E-06	5.24145E-05
ZNF667-AS1	1.45113E-07	1.23606E-07	4.85031E-06
ZNF835	3.81007E-09	8.75435E-09	5.61808E-07
ZNF883	7.67648E-06	1.56307E-05	8.8377E-05
ZSCAN1	2.45203E-05	2.60755E-05	8.4774E-06

Table S2. Two hundred and eighty-three genes were significantly predicting prognosis of UM patients by univariate Cox regression analysis (p-value < 0.0001).

Gene	Overall survival				Disease-specific survival				Progression-free survival			
	HR	HR.95L	HR.95H	coxPvalue	HR	HR.95L	HR.95H	coxPvalue	HR	HR.95L	HR.95H	coxPvalue
ABCB4	8.877375054	3.393019404	23.22644773	8.60173E-06	8.787176807	3.300260452	23.39647957	1.36E-05	7.846398367	3.382840377	18.19948933	1.59E-06
ABCB8	8.091592972	2.937960138	22.28548849	5.23424E-05	9.307687329	3.217934923	26.92193767	3.84E-05	7.986883134	3.281632358	19.43858886	4.68E-06
ABCC4	6.38971859	2.819350047	14.48153048	8.87458E-06	7.280232238	3.063511528	17.30098972	6.96E-06	4.727874249	2.330495494	9.591434513	1.68E-05
AC005840.2	15.75719391	4.406130653	56.35083917	2.22607E-05	16.27424731	4.390826987	60.31918956	3.00E-05	11.41926965	3.624685455	35.97545801	3.19E-05
AC010307.2	125.5364689	11.87535369	1327.068266	5.9039E-05	129.3126585	11.40736254	1465.874657	8.67E-05	132.3962815	17.05468709	1027.798122	2.97E-06
AC015818.2	4.473811401	2.176162919	9.197375933	4.60866E-05	4.478213319	2.123685907	9.443201779	8.20E-05	3.499586928	1.870565172	6.54727718	8.88E-05
AC016747.1	9.544636894	3.554714474	25.62796369	7.58125E-06	11.96299747	4.206174969	34.0245733	3.26E-06	10.87188383	4.385472264	26.95213899	2.59E-07
AC018529.1	2.102398694	1.480720952	2.985086598	3.25802E-05	2.123047469	1.474190721	3.057494863	5.22E-05	1.893454562	1.403847671	2.553817093	2.89E-05
AC018904.1	3.525882719	2.051758728	6.059118346	5.07617E-06	3.296980651	1.901116826	5.717734576	2.17E-05	2.392163686	1.558365873	3.67208189	6.64E-05
AC023790.2	193988012.2	48969.00459	7.68473E+11	6.3364E-06	104215927.5	21595.49968	5.02927E+11	1.99E-05	325917627.8	145681.1391	7.29142E+11	6.32E-07
AC025165.1	67.39964332	8.240230794	551.2845493	8.60674E-05	71.40087317	8.422744493	605.2759518	9.08E-05	78.19990946	11.80237907	518.1350134	6.23E-06
AC026369.2	5.859706367	2.485970357	13.81197431	5.30887E-05	6.63357217	2.763264376	15.9247447	2.29E-05	7.943339192	3.409390915	18.50671838	1.57E-06
AC092535.5	2.618563491	1.710705358	4.008214929	9.34439E-06	2.765625926	1.761397251	4.342397353	9.90E-06	3.275416128	2.085029079	5.145420234	2.63E-07
AC093908.1	420872.0936	1080.703927	163905501.6	2.08762E-05	846296.2244	2082.561484	343911718.9	8.47E-06	375230.4129	2129.208561	66126853.59	1.15E-06
AC097359.2	0.076095913	0.021961349	0.263671776	4.85689E-05	0.054825335	0.013071416	0.229953466	7.21E-05	0.161287736	0.066580233	0.390712567	5.31E-05
AC100791.3	2.71596739	1.766256637	4.176334688	5.33539E-06	2.874529624	1.831919963	4.510524875	4.36E-06	2.31422045	1.592132835	3.363799913	1.10E-05
AC103706.1	4.065340714	2.097313709	7.880077764	3.27663E-05	4.15881383	2.099160138	8.239358284	4.39E-05	3.23056289	1.866761509	5.590717686	2.78E-05
AC104129.1	2.432702647	1.711007626	3.458805254	7.37731E-07	2.499191646	1.728450582	3.613617277	1.12E-06	2.117821317	1.566893585	2.862458033	1.05E-06
AC124798.1	3.815426433	2.042097227	7.128690383	2.68467E-05	3.789913759	1.993336754	7.205729926	4.82E-05	3.833913663	2.13018124	6.900302048	7.40E-06
AC136475.3	6.603904261	2.996363396	14.55482721	2.84542E-06	5.90139528	2.61743191	13.30558633	1.87E-05	4.29966381	2.128092587	8.687173197	4.81E-05

AC141557.1	0.597307929	0.481926737	0.740313276	2.53118E-06	0.569832404	0.449472075	0.722423009	3.39E-06	0.666518957	0.559359412	0.794207643	5.72E-06
ACAN	6.036369842	2.688672527	13.55232387	1.31945E-05	6.613462439	2.883971582	15.16585174	8.15E-06	5.382048658	2.478509875	11.68704149	2.10E-05
ADAM11	2.828831435	1.772500157	4.514689184	1.30171E-05	2.990540517	1.837011917	4.868412939	1.05E-05	2.938309282	1.909997285	4.520248017	9.37E-07
ADAM12	3.907577049	2.040907774	7.481552372	3.91119E-05	4.426693217	2.208387922	8.873265715	2.75E-05	3.086698341	1.819998191	5.235008856	2.89E-05
ADAMTS2	2.193153384	1.501469118	3.203476984	4.85651E-05	2.214967394	1.498275882	3.274484102	6.69E-05	2.233893069	1.59127037	3.136034165	3.42E-06
ADAMTS9	692.5705951	31.93841917	15018.08924	3.09174E-05	889.0083077	38.74860821	20396.49442	2.16E-05	1531.368697	68.51176693	34229.01189	3.72E-06
ADCK5	4.704347835	2.351146323	9.412807844	1.20993E-05	4.92389884	2.37741059	10.19797753	1.78E-05	4.286668181	2.299196349	7.992150867	4.66E-06
ADGRB1	2.896022013	1.870273521	4.48434061	1.8759E-06	3.163406065	2.014840529	4.966714631	5.63E-07	3.518446518	2.220957137	5.573932829	8.36E-08
ADM2	4.601463686	2.496751951	8.480405128	9.91964E-07	4.700947532	2.495050779	8.85709737	1.68E-06	4.117375605	2.345628193	7.227395169	8.23E-07
AIFM2	6.104896042	2.780109422	13.40585928	6.55369E-06	6.361446522	2.80693412	14.41715413	9.32E-06	4.279461512	2.15375306	8.503198984	3.32E-05
AKR1D1	2.00385E+45	9.71617E+23	4.13271E+66	3.10351E-05	3.53E+47	1.38E+26	9.07E+68	1.34E-05	4.27E+37	6.7483E+19	2.70E+55	3.42E-05
AL023803.2	339.1704448	27.32076833	4210.591343	5.79593E-06	387.1923199	30.89654267	4852.254641	3.85E-06	100.9083139	14.75476499	690.1152143	2.55E-06
AL139339.1	226.1071029	15.86464425	3222.538193	6.36131E-05	279.8950981	17.24518401	4542.790954	7.41E-05	200.2300904	15.83754219	2531.459025	4.24E-05
AL158801.6	19.95718584	5.466633645	72.85823276	5.87017E-06	23.83301011	6.400151598	88.74983068	2.28E-06	13.49843478	3.843978235	47.40082548	4.89E-05
AL391422.4	0.172904546	0.072501444	0.412350156	7.56753E-05	0.147206889	0.057140807	0.379236299	7.24E-05	0.137131483	0.052652228	0.357155701	4.74E-05
ALG5	4.708957832	2.344578768	9.457683469	1.33161E-05	4.987225385	2.396083339	10.38044739	1.74E-05	4.082920522	2.203147156	7.566557661	7.84E-06
AMN	2.886128018	1.827699871	4.557496047	5.43789E-06	2.945629612	1.834194313	4.730542316	7.83E-06	2.385714216	1.580052175	3.602179987	3.53E-05
ANPEP	4.620532952	2.47840601	8.614135322	1.466E-06	4.963064664	2.61522417	9.418699607	9.54E-07	7.383191057	3.979498296	13.69808607	2.30E-10
ANXA2P2	4.184992861	2.140278553	8.183124213	2.86354E-05	4.560062193	2.283751209	9.105268175	1.70E-05	3.455892696	1.909752246	6.253792528	4.17E-05
APOM	0.124895533	0.046875737	0.332771177	3.17448E-05	0.102916465	0.035928551	0.294801725	2.29E-05	0.134791978	0.054983587	0.330441835	1.19E-05
ARC	2.731319724	1.85902983	4.012903568	3.07547E-07	2.828661079	1.89739718	4.216999785	3.33E-07	2.215050274	1.585241259	3.095079495	3.17E-06
ARHGDIG	2.467406954	1.620639178	3.756602433	2.5399E-05	2.742603474	1.760015978	4.273753141	8.28E-06	2.789199219	1.871792866	4.156246358	4.64E-07

ARMC9	2.405804405	1.629651225	3.551615676	9.99386E-06	2.479827635	1.639939428	3.74986112	1.67E-05	2.235996564	1.584180493	3.156004418	4.73E-06
ARPC1B	11.9813553	4.081923086	35.16795193	6.17804E-06	10.44298396	3.523204421	30.9536152	2.32E-05	5.369969134	2.37761256	12.12837153	5.27E-05
ATP8B3	12.38446188	4.430219505	34.6201573	1.60395E-06	15.15665119	5.13672052	44.72193384	8.47E-07	12.65279839	4.969466613	32.2153904	1.02E-07
AZGP1	0.52178064	0.380903837	0.71476055	5.09153E-05	0.49038853	0.345084771	0.696874887	7.06E-05	0.569825832	0.433171409	0.749591205	5.81E-05
BAG2	2.574244434	1.661281587	3.988929064	2.32139E-05	2.666414001	1.695924652	4.192263857	2.16E-05	2.401840852	1.599918941	3.605707346	2.37E-05
BATF3	10.56358957	3.694222511	30.20647083	1.09389E-05	9.024083513	3.019877732	26.96601998	8.19E-05	7.172429408	2.676589426	19.21988599	8.94E-05
BHLHA15	22.87750574	6.830809984	76.62052819	3.8618E-07	21.9168472	6.331312272	75.86866208	1.10E-06	22.90361056	6.626602523	79.16204038	7.48E-07
BRD9P2	19.22890643	5.399624451	68.47714056	5.06046E-06	27.62559803	7.227563365	105.5921101	1.23E-06	17.51902623	6.064892006	50.60539902	1.22E-07
BST1	6.785930077	2.853217894	16.13926756	1.47933E-05	8.140587352	3.21271329	20.62716354	9.86E-06	4.905591945	2.34311563	10.27044164	2.46E-05
BUD23	204.9885868	31.31769821	1341.743586	2.80822E-08	146.336469	22.20024935	964.6000742	2.20E-07	24.32894517	6.347137189	93.25425866	3.23E-06
BX470102.1	37.8966119	7.524899332	190.8534759	1.04918E-05	34.41633877	6.633101581	178.5717224	2.53E-05	22.75176909	4.984843056	103.843389	5.49E-05
CA12	2.01276314	1.591150843	2.546091389	5.4496E-09	2.018250995	1.57970744	2.578538895	1.93E-08	1.698605727	1.393223824	2.070924546	1.61E-07
CALCOCO1	0.067936282	0.025045015	0.184281715	1.27906E-07	0.065430563	0.023149236	0.184937357	2.69E-07	0.16013318	0.07097861	0.361272719	1.02E-05
CALHM2	4.117429767	2.152813126	7.874918488	1.88955E-05	4.755004752	2.28914654	9.877074183	2.91E-05	3.981665112	2.242158306	7.070712635	2.41E-06
CAMK1	18.44935557	4.308533773	79.00105671	8.55709E-05	29.25362659	5.507929638	155.3713873	7.41E-05	10.98762701	3.490152424	34.59102433	4.20E-05
CAMSAP3	0.259407094	0.136053978	0.49459811	4.16571E-05	0.273317228	0.143625282	0.520119479	7.78E-05	0.329846659	0.200265266	0.543273532	1.32E-05
CARD11	2.36818971	1.611519222	3.480146204	1.13606E-05	2.409357064	1.614559764	3.595408229	1.67E-05	2.02434702	1.472210484	2.783556361	1.42E-05
CCDC188	5.510372264	2.431513571	12.48777833	4.34032E-05	5.663744617	2.427536651	13.21421989	6.03E-05	8.721569745	3.90164595	19.49581786	1.31E-07
CCDC74A	4.369676799	2.437042942	7.834935939	7.42041E-07	4.294399253	2.374422574	7.766884102	1.43E-06	2.77707063	1.736299656	4.441699485	2.02E-05
CDC25B	2.499569206	1.641031363	3.807268012	1.98049E-05	2.64093203	1.697445161	4.10883494	1.66E-05	2.656376945	1.823752649	3.869131309	3.55E-07
CDCA7L	0.226652284	0.118199484	0.43461491	7.87347E-06	0.203877436	0.101153488	0.410920173	8.71E-06	0.29642786	0.170653359	0.514900359	1.59E-05
CDH24	4.389650126	2.177993741	8.847145821	3.52142E-05	5.315825586	2.492460292	11.33739291	1.54E-05	5.850086669	2.919127422	11.72388495	6.35E-07

CGREF1	6.278660468	2.781788871	14.17130454	9.72497E-06	7.161961765	3.018168923	16.99497199	7.99E-06	5.389566066	2.609260025	11.13243682	5.33E-06
CHAC1	1.6736408	1.325776266	2.11277996	1.4772E-05	1.684644586	1.3199957	2.150027748	2.78E-05	1.540187285	1.2706961	1.866832576	1.08E-05
CHST9	5.807698541	2.602737786	12.95918572	1.74024E-05	6.550643757	2.849425161	15.05950541	9.63E-06	6.550939852	3.055011592	14.04734865	1.37E-06
CNIH3-AS2	8.63854484	3.515084525	21.22977596	2.60105E-06	8.24275272	3.227220199	21.05309468	1.04E-05	6.476106322	2.719644726	15.42111464	2.44E-05
COQ2	5.106600651	2.342085055	11.13425413	4.1345E-05	5.396486698	2.395498219	12.15699868	4.74E-05	8.345828711	3.613017447	19.27830626	6.79E-07
CORO6	3.327399358	1.897804203	5.833892912	2.71191E-05	3.453912539	1.940497358	6.147656827	2.51E-05	4.555756028	2.620126975	7.921338617	7.75E-08
COX6C	4.158652303	2.336451662	7.401988777	1.26721E-06	4.175167383	2.305926457	7.559661161	2.38E-06	3.486756799	2.105321603	5.774639349	1.22E-06
CRABP2	2.317316678	1.617840053	3.319213525	4.55802E-06	2.294177583	1.589725266	3.310792684	9.13E-06	2.021113519	1.455745335	2.806053887	2.63E-05
CTF1	0.293312332	0.171654034	0.50119489	7.22407E-06	0.271916874	0.151369173	0.48846661	1.32E-05	0.389531524	0.263447872	0.575957615	2.30E-06
CTNNBIP1	0.165208825	0.067071635	0.406937389	9.04748E-05	0.143256722	0.05440364	0.377226388	8.37E-05	0.157216826	0.069547099	0.355401315	8.75E-06
CYC1	4.03800087	2.139483732	7.62120823	1.65629E-05	4.447925894	2.257114405	8.765193611	1.62E-05	3.6030286	2.064457252	6.288246018	6.45E-06
CYTOR	2.86156855	1.791223364	4.571498302	1.08919E-05	2.785846964	1.71929275	4.514032474	3.17E-05	2.363132829	1.53266612	3.643583357	9.91E-05
DDRGK1	5.009522611	2.248623902	11.16029976	8.05781E-05	6.065528196	2.557867245	14.38332359	4.28E-05	3.923396527	1.997837247	7.704851996	7.19E-05
DDX39A	5.536389865	2.602546091	11.77754847	8.85163E-06	5.267080768	2.436631281	11.38544844	2.40E-05	5.296778845	2.762554869	10.15576793	5.18E-07
DECR1	3.425630575	1.935233571	6.063839017	2.37908E-05	3.471266735	1.927328215	6.252019066	3.39E-05	3.070639512	1.858285592	5.073938609	1.20E-05
DENND3	3.578080613	1.957627134	6.53988732	3.42797E-05	3.731600904	2.001748499	6.956341076	3.41E-05	4.466281913	2.390689124	8.343901317	2.69E-06
DLL4	2.724019653	1.751594322	4.236302308	8.67291E-06	2.756228609	1.740352311	4.365090963	1.55E-05	2.284501673	1.590429882	3.281469969	7.78E-06
DNAH17-AS1	1736.820377	46.86571954	64365.70381	5.18125E-05	2494.70888	66.6984085	93309.15886	2.31E-05	1563.070965	53.88877316	45337.65936	1.87E-05
DTYMK	14.59040461	4.090210293	52.04620094	3.61504E-05	12.98983537	3.579191102	47.14356348	9.67E-05	10.82104349	3.741709903	31.29451112	1.11E-05
ECM1	1.66147111	1.312467913	2.103279039	2.44169E-05	1.707896896	1.328782026	2.19517705	2.92E-05	1.568228921	1.27238124	1.932865615	2.46E-05
EDNRB	0.431669115	0.296680755	0.628076548	1.1294E-05	0.372538431	0.244804209	0.566921963	4.04E-06	0.49984743	0.36321824	0.687871438	2.08E-05
EFS	0.320836024	0.191568038	0.537332612	1.55526E-05	0.310339901	0.179178018	0.5375149	2.98E-05	0.467922649	0.320634193	0.682870419	8.22E-05

ELOC	2.925142494	1.810166267	4.726890985	1.16827E-05	2.946952164	1.79698474	4.832832946	1.85E-05	3.033072057	1.934544551	4.755396355	1.33E-06
EMC7	4.845693095	2.309246772	10.16813874	3.00433E-05	4.713128907	2.198204037	10.10533313	6.78E-05	4.429377492	2.269938626	8.643134552	1.28E-05
ENPP2	0.40633097	0.271636135	0.607816253	1.1697E-05	0.3666603	0.230754637	0.582609203	2.17E-05	0.523585694	0.38282532	0.716102004	5.12E-05
ENTPD6	0.153599743	0.064767262	0.364271707	2.11878E-05	0.151598787	0.062663738	0.366754251	2.85E-05	0.228202931	0.115593103	0.450516307	2.07E-05
ERFE	3.294383895	1.896036968	5.724026183	2.34012E-05	3.569368602	2.007541947	6.346264514	1.47E-05	3.820563985	2.354881737	6.198489264	5.67E-08
ERVMER34-1	4.332169736	2.199431994	8.532973362	2.24587E-05	4.597314853	2.310726714	9.146604716	1.38E-05	4.623005919	2.471310561	8.648117345	1.66E-06
FABP5	2.673537438	1.724188791	4.14560312	1.11256E-05	2.752291738	1.737723407	4.359214926	1.60E-05	2.616302715	1.757340986	3.895111964	2.17E-06
FABP5P7	6.950594092	2.747875517	17.58113056	4.22414E-05	7.069900067	2.78607884	17.9404424	3.85E-05	7.546833003	3.395303167	16.77455166	7.07E-07
FADS1	4.60112446	2.228983723	9.497757243	3.66578E-05	4.960782576	2.308296613	10.66126581	4.08E-05	4.25169387	2.300104103	7.859166349	3.89E-06
FAM53A	10.18661801	3.30921563	31.35703386	5.20908E-05	10.51874276	3.296492037	33.56414881	7.04E-05	10.26799859	3.582517996	29.42952282	1.46E-05
FAM72A	7625.531782	86.56754306	671715.2053	9.14176E-05	10645.72704	104.8678314	1080707.998	8.36E-05	18324.63192	290.8088872	1154683.195	3.43E-06
FKBP10	3.054512459	1.834061228	5.08709645	1.78262E-05	3.278884393	1.910350404	5.627806731	1.64E-05	2.85594634	1.828345801	4.461097835	3.99E-06
FKBP11	3.116112608	1.918888474	5.060303358	4.33488E-06	2.980144998	1.82212622	4.874121295	1.36E-05	2.451636617	1.654121914	3.633663307	7.94E-06
FKBP5	3.366839283	1.845582817	6.142020099	7.56439E-05	3.73772265	1.989479443	7.022224159	4.17E-05	4.378105019	2.545565017	7.529881749	9.44E-08
FOXD1	4.972036559	2.672148628	9.251411874	4.14124E-07	5.335724694	2.822347963	10.08733097	2.56E-07	4.625988976	2.649433578	8.077112855	7.19E-08
FOXF1	8.397663781	3.172590638	22.22812995	1.83017E-05	9.152945422	3.398735979	24.64928445	1.18E-05	7.150281619	2.870931967	17.80833814	2.39E-05
FRMPD3	35.40202286	5.904411806	212.2655506	9.49648E-05	42.81490821	7.062069086	259.5721372	4.39E-05	37.98623551	7.680099483	187.8822132	8.22E-06
GCNA	17.49338704	5.036861769	60.75580474	6.63313E-06	18.21251761	5.119192966	64.79454863	7.40E-06	9.07069739	3.312175503	24.84093946	1.79E-05
GEM	1.87586383	1.389308687	2.532817321	4.02145E-05	1.966458274	1.43273698	2.699000721	2.84E-05	1.977036566	1.496828369	2.611303784	1.58E-06
GGT1	2.16234957	1.495484495	3.126582506	4.14799E-05	2.166664269	1.484233931	3.162866687	6.18E-05	2.242129363	1.622919008	3.097593939	9.76E-07
GLA	7.514212998	3.242221928	17.41503149	2.56665E-06	7.668835468	3.194100361	18.41239497	5.15E-06	5.771688141	2.77746246	11.99381971	2.64E-06
GPR143	5.029740176	2.301255587	10.99325359	5.14099E-05	5.30759364	2.386797972	11.80265384	4.25E-05	4.103637536	2.148536871	7.837818032	1.90E-05

GPR63	1806.530224	107.1137357	30468.09478	1.96771E-07	2367.026105	135.514954	41344.60747	1.02E-07	255.5308489	17.31739789	3770.544233	5.43E-05
GPR89A	74.05943164	9.718460825	564.3691438	3.25818E-05	66.1821419	8.273630158	529.401946	7.76E-05	30.57591624	5.552797984	168.3631669	8.51E-05
GREB1	2.918774613	1.740891206	4.893611511	4.85186E-05	3.166774359	1.821499016	5.505608156	4.41E-05	3.331966745	2.018666148	5.499672347	2.51E-06
GRIN2A	3.50911007	2.054038666	5.994947263	4.34332E-06	3.58293825	2.082397688	6.164742967	4.04E-06	2.864497416	1.713452415	4.788779293	5.97E-05
GUCY2C	17830.65913	219.7068555	1447075.487	1.27753E-05	19778.10156	221.152437	1768794.894	1.60E-05	1813.716792	42.1572339	78030.94027	9.25E-05
GUSB	18.36767819	6.215019646	54.28327204	1.40628E-07	22.31865591	7.056475459	70.59082182	1.25E-07	13.2658257	5.314336087	33.11460333	3.04E-08
HDAC11	0.182527972	0.083645863	0.398303748	1.93596E-05	0.161368069	0.06879668	0.378501604	2.75E-05	0.222351275	0.114395459	0.432185769	9.25E-06
HIP1	6.107713381	2.508906935	14.86869131	6.71036E-05	7.344805883	2.773991403	19.44713073	5.98E-05	6.639434204	2.93432505	15.02290503	5.52E-06
HM13	3.964653458	2.131816468	7.37327874	1.3538E-05	4.330410544	2.24914546	8.337591238	1.16E-05	2.868018626	1.70295612	4.830148433	7.44E-05
HMCES	0.023986894	0.00473697	0.121463946	6.5693E-06	0.016410146	0.002743987	0.098139296	6.67E-06	0.088390242	0.027057148	0.288753086	5.91E-05
HPSE	3.540317173	1.899875444	6.597193373	6.86572E-05	3.674569149	1.940454028	6.958401608	6.47E-05	3.596314399	2.028708395	6.375227355	1.18E-05
HTR2B	1.453577227	1.22407848	1.726103995	1.98935E-05	1.478379148	1.231332461	1.774991705	2.78E-05	1.3885258	1.197474069	1.610058997	1.39E-05
HTRA3	2.872887211	1.808824945	4.562896455	7.79173E-06	3.03051408	1.864086005	4.926819665	7.76E-06	3.279124119	2.101553339	5.116527279	1.68E-07
IAH1	12.42594361	4.211285586	36.66435619	5.0109E-06	13.39816829	4.307637673	41.6727049	7.38E-06	10.15269423	3.995552673	25.79798305	1.11E-06
IDH2	5.348924811	2.73383751	10.46550738	9.74281E-07	5.448315346	2.711656319	10.94686665	1.92E-06	3.91018165	2.260035415	6.765168561	1.09E-06
IFT122	0.169308088	0.074004829	0.387342677	2.5976E-05	0.169920951	0.072340224	0.399129668	4.74E-05	0.212191222	0.103390365	0.435486562	2.38E-05
IGFBP2	2.174093054	1.470209125	3.214971616	9.98903E-05	2.433217201	1.582484214	3.741298581	5.10E-05	2.441030348	1.663734675	3.581478014	5.05E-06
IGHMBP2	0.053529613	0.015042404	0.190489458	6.17663E-06	0.044072991	0.011530918	0.168453936	5.03E-06	0.170478481	0.070604178	0.411631624	8.37E-05
IL10RB	11.01886655	3.567764597	34.03123067	3.03695E-05	10.46718817	3.244002158	33.77372234	8.53E-05	8.386372725	3.274382018	21.47924314	9.34E-06
ISG20	3.335132325	2.108978589	5.274168114	2.58941E-07	3.131877749	1.966071993	4.988961882	1.54E-06	2.351251904	1.600158654	3.454898364	1.34E-05
ISM1	1.931502551	1.426377397	2.615508427	2.08145E-05	1.989694074	1.455351423	2.72022444	1.62E-05	2.468388455	1.802290564	3.380665517	1.79E-08
ITGA5	4.115575276	2.266905864	7.471840856	3.32399E-06	4.631939945	2.426402019	8.842255935	3.37E-06	3.482480616	2.047212293	5.923992974	4.16E-06

ITGB3	136.4268051	14.68578802	1267.366322	1.54173E-05	187.1102658	19.04695165	1838.102611	7.19E-06	56.97765556	8.581871472	378.2919895	2.84E-05
JAG2	4.209973418	2.089705945	8.481516848	5.76287E-05	4.725814245	2.215813469	10.07906152	5.85E-05	4.284269159	2.221546821	8.262244151	1.41E-05
JPH1	3.853672657	2.105842995	7.052184317	1.21263E-05	4.449912244	2.353082894	8.415223718	4.38E-06	7.799475313	3.933259909	15.46600443	4.08E-09
JUP	0.184288417	0.08332523	0.407586281	2.96527E-05	0.164110885	0.068793057	0.391498556	4.62E-05	0.266426022	0.144127638	0.492499749	2.45E-05
KATNAL2	10.61803889	3.830195743	29.43524494	5.58987E-06	11.22614692	3.903682979	32.28396757	7.23E-06	7.229521134	3.053209714	17.11837074	6.86E-06
KCNN3	15603.26323	335.1341647	726460.7702	8.34169E-07	19811.24981	387.0096986	1014149.311	8.33E-07	1367.77534	38.56289305	48513.20097	7.31E-05
KCP	7259908.252	8002.008344	6586629951	5.45586E-06	15109515.54	13595.42113	16792231572	3.84E-06	4628396.468	8213.470983	2608160899	2.04E-06
KCTD17	3.595316565	1.953629828	6.616556022	3.92393E-05	4.12071317	2.06051139	8.240807169	6.22E-05	2.737888621	1.681379045	4.45826545	5.15E-05
KDELR3	4.876110214	2.568753255	9.256027518	1.26639E-06	5.050643403	2.567633086	9.93483022	2.71E-06	3.842710292	2.265965925	6.51661273	5.87E-07
KDM7A-DT	5.902927507	2.605105397	13.37548692	2.09815E-05	6.863425936	2.835130034	16.61532805	1.95E-05	4.474173249	2.231019941	8.972679216	2.44E-05
KHDRBS3	3.910590814	2.078175104	7.358725685	2.36033E-05	4.112834326	2.157357219	7.840799864	1.74E-05	3.757125255	2.138533043	6.600781891	4.15E-06
KIF20A	4.003178394	1.991911867	8.045254168	9.8223E-05	4.284455342	2.088560107	8.789097101	7.22E-05	4.528119341	2.536400737	8.083842771	3.26E-07
KRTCAP2	10.81733299	4.605354673	25.40839988	4.62174E-08	10.2420075	4.283846192	24.48704108	1.68E-07	3.978404747	2.012716151	7.863853192	7.13E-05
LAMA1	4.106893616	2.226186536	7.576442897	6.14436E-06	4.235369847	2.269581935	7.903815878	5.77E-06	3.800193372	2.175567695	6.638023584	2.71E-06
LAMA5	6.484378443	2.574118564	16.33458706	7.31533E-05	8.33387151	3.040636772	22.84173335	3.76E-05	4.76075562	2.303977743	9.837245233	2.51E-05
LDLRAD3	3.009267973	1.76111084	5.142035088	5.5686E-05	3.128519141	1.789415225	5.469737756	6.30E-05	3.026402023	1.846417572	4.960475539	1.12E-05
LFNG	2.706384861	1.663465108	4.403169613	6.09006E-05	2.967989062	1.802358349	4.887462627	1.91E-05	2.748055762	1.791631058	4.215047754	3.63E-06
LHFPL6	2.439071815	1.607967647	3.699745656	2.73722E-05	2.66660943	1.709587315	4.159369801	1.53E-05	2.476312574	1.682959853	3.643654334	4.19E-06
LIMS2	0.395037324	0.254192917	0.613921464	3.64545E-05	0.354147169	0.215943319	0.580801561	3.91E-05	0.421677661	0.28794161	0.617528151	9.14E-06
LINC00404	2.361980023	1.630609384	3.421389381	5.46153E-06	2.608908105	1.736743047	3.919060745	3.86E-06	2.230039936	1.62181774	3.066360661	7.98E-07
LINC00518	0.439960504	0.298074693	0.649385037	3.57569E-05	0.398240708	0.259966239	0.610062528	2.33E-05	0.358462171	0.237489782	0.541055395	1.04E-06
LINC01252	12.16754153	3.873512566	38.22088204	1.87944E-05	13.7311	4.308777444	43.75791267	9.42E-06	18.58195333	6.24753061	55.26807486	1.48E-07

LINC01278	0.14137188	0.05515255	0.36237668	4.63014E-05	0.137535569	0.051329382	0.368522512	7.98E-05	0.164428635	0.069681518	0.388004978	3.77E-05
LINC01842	9.006373742	3.092075303	26.23311532	5.59022E-05	9.54922904	3.216970579	28.34585304	4.81E-05	6.61200298	2.563967136	17.05114812	9.31E-05
LINC01971	43.06262945	10.20332531	181.7436962	3.03107E-07	41.67266068	9.42166525	184.3209881	8.80E-07	12.87004228	3.688003895	44.91263919	6.16E-05
LINC02568	901588.7583	3494.995416	232578928.6	1.29936E-06	790258.3873	2951.023001	211624348.1	1.92E-06	4156026.896	12790.46305	1350424883	2.41E-07
LMCD1	0.097639012	0.033671028	0.283132922	1.84419E-05	0.103977064	0.035712551	0.302729136	3.30E-05	0.251113894	0.130253908	0.484117435	3.69E-05
LPAR1	12.52926886	3.71585539	42.24668663	4.57018E-05	13.81619032	4.019152331	47.49437172	3.07E-05	9.762614654	3.235910994	29.45341978	5.25E-05
LTA4H	0.194630725	0.090294644	0.419527867	2.9583E-05	0.166270074	0.07164363	0.385878517	2.96E-05	0.296946441	0.161590606	0.545682643	9.19E-05
LTBR	6.738031046	2.873860262	15.79793665	1.14342E-05	7.429365184	3.041741857	18.14600635	1.08E-05	5.595765352	2.72973614	11.47092183	2.58E-06
LURAP1	0.299191809	0.172456567	0.51906251	1.76495E-05	0.255925161	0.14151609	0.462828558	6.53E-06	0.295878795	0.178691445	0.489918592	2.21E-06
LYN	2.993490564	1.766010144	5.074141724	4.65663E-05	3.047319327	1.761460525	5.271849667	6.77E-05	2.96663667	1.811409566	4.858610275	1.56E-05
MALSU1	14.04696723	3.921299863	50.31935718	4.93155E-05	16.23259858	4.227249725	62.33302358	4.91E-05	11.63003259	4.008633619	33.74158649	6.34E-06
MANEAL	0.123166064	0.044418193	0.341524012	5.70788E-05	0.129231787	0.046106416	0.362224098	9.98E-05	0.208660993	0.10159903	0.428541589	1.98E-05
MAP4K3-DT	3.782114111	1.992772274	7.178134368	4.71934E-05	3.879468168	2.003662476	7.511381505	5.78E-05	4.031488809	2.211284831	7.349981237	5.37E-06
MAPK12	3.70200997	2.0266826	6.762222078	2.06251E-05	4.255790757	2.232640104	8.112259084	1.08E-05	4.00923893	2.273528856	7.070065004	1.60E-06
MATK	2.153011431	1.540109886	3.009823042	7.24093E-06	2.166615976	1.528270654	3.071592571	1.41E-05	1.915464208	1.446852162	2.535852126	5.61E-06
MGLL	1.862811064	1.44325981	2.404324597	1.77026E-06	1.933104714	1.491435462	2.505568582	6.34E-07	1.786183196	1.402004249	2.275635337	2.67E-06
MIB2	0.223318848	0.107330036	0.464653792	6.06506E-05	0.208561224	0.097874907	0.444422227	4.89E-05	0.198825825	0.102990005	0.383840244	1.49E-06
MIRLET7BHG	76.63123502	8.852725565	663.3376509	8.13759E-05	102.9533688	10.9159986	970.9964744	5.17E-05	89.34941324	12.63680746	631.75115	6.74E-06
MMP16	46.83433053	7.663318209	286.2277745	3.11454E-05	62.38189174	9.927467832	391.9932537	1.05E-05	33.99536809	5.767141749	200.3913034	9.79E-05
MMP9	2.018989015	1.534609952	2.656255838	5.16961E-07	1.985697614	1.491845824	2.643031171	2.58E-06	1.721036798	1.32807358	2.230273762	4.04E-05
MRPS11	9.168197377	3.356151418	25.04530716	1.55043E-05	9.262083985	3.277111363	26.17738314	2.68E-05	7.509487967	3.091736232	18.23972205	8.47E-06
MTMR14	0.043425963	0.010267305	0.183671794	2.01593E-05	0.030535198	0.006040582	0.154355713	2.44E-05	0.071131929	0.02218511	0.228069696	8.73E-06

MYEOV	1.81571434	1.381862194	2.385779551	1.85556E-05	1.85945275	1.39704912	2.474905485	2.12E-05	1.892893082	1.458729598	2.456277177	1.58E-06
MYH14	0.389030419	0.276679743	0.547003063	5.64741E-08	0.38887734	0.272690324	0.554568946	1.83E-07	0.46621326	0.351562573	0.618253536	1.16E-07
NATD1	0.088104478	0.030380805	0.255503402	7.7555E-06	0.083228116	0.027523712	0.251670965	1.06E-05	0.13493371	0.053057785	0.343156168	2.60E-05
NCAPH	8.049292973	2.894318522	22.38562096	6.43093E-05	9.050324212	3.147516736	26.02317167	4.36E-05	11.617987	4.58723757	29.42459814	2.31E-07
NCR3LG1	4.561721537	2.137948451	9.73330455	8.66998E-05	5.12729884	2.359433313	11.1421642	3.66E-05	5.502823706	2.695557665	11.23369354	2.82E-06
NDUFA6	5.065635759	2.420359444	10.60200612	1.66491E-05	4.757541759	2.20711153	10.25512453	6.88E-05	4.373345055	2.270151222	8.425054145	1.03E-05
NDUFB9	3.836602158	2.056637549	7.157078371	2.37257E-05	3.977174126	2.085958029	7.583045204	2.75E-05	3.768005586	2.116984955	6.706644779	6.50E-06
NECAB2	2.19264738	1.541104078	3.11964818	1.27715E-05	2.255011323	1.557758342	3.264354892	1.64E-05	1.797399949	1.34023071	2.410515259	9.02E-05
NICN1	0.131079324	0.057276181	0.299981402	1.50685E-06	0.128120608	0.054403458	0.301725124	2.58E-06	0.177298632	0.083431345	0.376774518	6.86E-06
NKX3-2	397886.1702	1479.01427	107039808.7	6.27243E-06	604561.0189	2192.096572	166732629.5	3.44E-06	12781.22715	115.8501406	1410095.548	8.14E-05
NQO1	2.073706277	1.518667571	2.831599097	4.45493E-06	2.147999724	1.542820836	2.99056294	5.95E-06	1.76500575	1.373617181	2.267913755	8.93E-06
ORAI2	25.55602115	6.133467885	106.4830255	8.54948E-06	28.78232336	6.34911155	130.4784349	1.32E-05	9.217431625	3.041001184	27.93851124	8.65E-05
ORMDL2	8.259971084	3.361937726	20.29398754	4.14991E-06	7.796422908	3.101008425	19.60143341	1.27E-05	5.385156304	2.494630284	11.6249324	1.80E-05
OTULINL	5.500321891	2.436063397	12.41902856	4.08302E-05	5.53797812	2.386595824	12.8506056	6.74E-05	4.712988477	2.221116259	10.00049425	5.37E-05
P2RX4	7.271488636	3.102655592	17.04170682	4.98183E-06	6.769054923	2.816990258	16.26562408	1.91E-05	11.62411172	4.511753659	29.94843768	3.77E-07
PACSIN3	0.168248118	0.068743313	0.411784476	9.50719E-05	0.140772348	0.053987415	0.367064322	6.08E-05	0.192919893	0.091380118	0.407288654	1.59E-05
PADI1	5.932487533	2.892536473	12.16731704	1.18531E-06	5.370666177	2.580714525	11.17677097	6.94E-06	3.610693202	1.895404867	6.878269454	9.44E-05
PAPSS2	4.555196549	2.355735641	8.808210581	6.58231E-06	4.686579775	2.362026992	9.298805668	9.93E-06	4.030417878	2.267629415	7.163546285	2.03E-06
PARP8	4.746787471	2.48212658	9.07769631	2.49898E-06	4.647649315	2.390901794	9.034517525	5.89E-06	3.062236544	1.772483317	5.290482883	6.03E-05
PDCD4-AS1	0.299781883	0.163802383	0.548643897	9.35773E-05	0.235414247	0.119021747	0.46562808	3.23E-05	0.240212382	0.127481157	0.452631509	1.02E-05
PGGHG	3.871641728	2.096635109	7.149365002	1.52031E-05	3.574278677	1.914280949	6.673768584	6.38E-05	2.775863414	1.660811406	4.639550082	9.79E-05
PI15	2239.026864	67.24761681	74548.98088	1.61073E-05	2462.440896	73.47890096	82521.85442	1.31E-05	586.9847475	38.3443813	8985.699653	4.66E-06

PLCD1	0.146407392	0.063242618	0.338934807	7.24972E-06	0.135718292	0.055430173	0.332300154	1.23E-05	0.201649038	0.101571768	0.400331072	4.73E-06
PLEKHG2	7.078810456	2.914632696	17.19240903	1.54086E-05	7.992961845	3.140687054	20.34186723	1.29E-05	7.987683725	3.433948472	18.58009571	1.41E-06
PLEKHG4B	3.202669842	2.026722152	5.060927621	6.16776E-07	3.542060327	2.180462849	5.753912003	3.24E-07	2.614894279	1.788428177	3.82328582	7.08E-07
PLXNB1	0.256626343	0.136786166	0.48146009	2.26712E-05	0.216162562	0.105050798	0.444796747	3.18E-05	0.295011229	0.172657471	0.504071007	7.96E-06
POMGNT2	0.112116581	0.043802333	0.286973933	5.03565E-06	0.11811045	0.045838836	0.304328812	9.71E-06	0.225801197	0.114982291	0.443426378	1.55E-05
POMP	8.417334195	3.35777829	21.10071269	5.53966E-06	7.870803632	3.065611737	20.20789165	1.80E-05	5.513117272	2.371736346	12.8152786	7.29E-05
POP7	32.4540445	7.467142114	141.0532957	3.45301E-06	32.41731782	6.986907779	150.4073802	8.88E-06	16.09921097	4.885062888	53.05655215	4.95E-06
POR	4.309650029	2.129703017	8.720973405	4.86459E-05	4.839409387	2.303097909	10.16886131	3.15E-05	3.792570773	2.049590889	7.017787376	2.18E-05
PPIB	4.166245954	2.099185008	8.268735382	4.49802E-05	4.232207816	2.089298867	8.573011396	6.18E-05	3.402957485	1.839700545	6.294567711	9.52E-05
PPM1K	2.49755248	1.625994631	3.836278591	2.91639E-05	2.649150934	1.66841245	4.206394333	3.63E-05	2.061360998	1.43885339	2.953191197	8.03E-05
PRKCD	0.265343444	0.13933301	0.505315597	5.41831E-05	0.240898009	0.120468108	0.481719617	5.68E-05	0.309185127	0.1762561	0.542366719	4.25E-05
PSMG3	6.135080662	2.832156888	13.28994693	4.23303E-06	5.706205044	2.578249219	12.62902584	1.73E-05	4.386417069	2.163995198	8.89126497	4.11E-05
PTGS2	657.3488686	32.69619599	13215.83511	2.25984E-05	659.470044	29.82505479	14581.72473	3.97E-05	760.5749089	43.47814689	13304.9413	5.53E-06
PTP4A3	1.901607016	1.41243326	2.560198308	2.27731E-05	2.05001056	1.487101169	2.825996902	1.17E-05	2.096481842	1.578185628	2.784993119	3.24E-07
PVT1	4.223492555	2.1835071	8.16937548	1.86891E-05	4.303778259	2.178606672	8.501996962	2.65E-05	3.258455272	1.839218194	5.772850006	5.16E-05
PXDN	2.672297995	1.680074267	4.250512442	3.30894E-05	2.930180761	1.792531643	4.789850894	1.81E-05	3.047594333	1.962042947	4.733755309	7.06E-07
RAB2A	2.300124441	1.521882727	3.476333853	7.72325E-05	2.3412557	1.529283375	3.584344367	9.04E-05	2.334530362	1.587853502	3.432326725	1.62E-05
RAB31	2.718533409	1.730868643	4.269777447	1.41382E-05	2.869657496	1.760531318	4.677527778	2.35E-05	2.447895859	1.632743574	3.670015447	1.47E-05
RAB5IF	4.976883254	2.26340877	10.94339089	6.55416E-05	5.229581284	2.309080627	11.84390016	7.30E-05	4.605734589	2.331655891	9.097736586	1.10E-05
RAB7B	5.0259849	2.502555897	10.09389011	5.67084E-06	5.04433659	2.496349508	10.19301647	6.52E-06	4.153784349	2.169407753	7.953287895	1.73E-05
RBP7	0.446182461	0.301614807	0.660043154	5.35838E-05	0.392213747	0.256573811	0.599560893	1.54E-05	0.464605758	0.329221175	0.655664117	1.29E-05
RHOF	809.9834223	36.08349591	18182.08375	2.45442E-05	941.0875938	37.71606503	23481.92629	3.02E-05	2668.792665	132.7764715	53642.44292	2.56E-07

RNA5SP18	16.51559733	4.07358765	66.95939268	8.61662E-05	19.37777353	4.725468063	79.46262715	3.84E-05	12.55730943	3.902763562	40.40368257	2.20E-05
RNF208	0.213242569	0.110087647	0.413056274	4.62634E-06	0.222645459	0.114445059	0.433142336	9.68E-06	0.377361562	0.240400892	0.592351165	2.27E-05
RPS12	0.350460694	0.206752083	0.59405785	9.85569E-05	0.321200604	0.184722876	0.558511378	5.73E-05	0.309885466	0.190632526	0.503738811	2.29E-06
RRM2	4.050201835	2.06275514	7.952536191	4.84066E-05	4.119682254	2.062055515	8.230516469	6.08E-05	5.177421323	2.795436035	9.589091367	1.70E-07
RTL8B	0.205958049	0.096778287	0.438308214	4.12251E-05	0.170522756	0.076197033	0.381616049	1.68E-05	0.259687848	0.137106919	0.491862691	3.51E-05
S100A3	5.936124419	2.714134353	12.98298777	8.17232E-06	6.040801982	2.736141058	13.33677169	8.55E-06	4.758187842	2.263583609	10.00199482	3.87E-05
S1PR5	203.4655762	16.29776465	2540.117715	3.67717E-05	222.2050182	16.71084943	2954.671473	4.26E-05	115.5349115	11.40940358	1169.939838	5.80E-05
SCAP	0.124568517	0.048838602	0.317726443	1.3007E-05	0.13817797	0.053746945	0.35524161	3.99E-05	0.225429963	0.107248743	0.473839287	8.47E-05
SCLY	775.6583224	31.16423187	19305.65257	4.97039E-05	1197.92128	41.33821549	34714.01404	3.68E-05	486.3638661	29.70892113	7962.248416	1.44E-05
SCX	2.623361607	1.69079547	4.070288952	1.68197E-05	2.72458584	1.743562593	4.25758618	1.08E-05	2.51130578	1.661145855	3.796570121	1.26E-05
SEC24D	3.791054164	1.949703376	7.371424727	8.56849E-05	4.00707234	2.020486389	7.9469126	7.09E-05	4.411900139	2.2866593	8.512358108	9.58E-06
SERPINB9	0.331005878	0.203739907	0.537768437	7.99569E-06	0.299352202	0.178737534	0.50135939	4.56E-06	0.419142739	0.272265092	0.645255824	7.81E-05
SFXN3	5.245920112	2.397417122	11.478886	3.34615E-05	5.840572509	2.465374388	13.83655456	6.06E-05	4.265772155	2.202891052	8.260423077	1.69E-05
SH3PXD2B	4.577700863	2.159675949	9.703004381	7.22456E-05	4.934730863	2.258357123	10.78286886	6.26E-05	4.003867763	2.051065221	7.815917746	4.81E-05
SHC1	4.464999124	2.115509243	9.423838368	8.63627E-05	5.000913897	2.272007939	11.00750547	6.37E-05	4.934904709	2.445548538	9.958209417	8.33E-06
SIRT3	0.012583232	0.002763116	0.057304049	1.54302E-08	0.010349122	0.002124683	0.050409558	1.53E-08	0.062824277	0.018978569	0.207965618	5.86E-06
SLC1A1	2.730951008	1.688969339	4.415766014	4.1726E-05	2.893127358	1.758939316	4.758655307	2.86E-05	3.068887023	1.938498274	4.858434845	1.72E-06
SLC25A26	0.048806751	0.011549927	0.206243633	4.00947E-05	0.032319147	0.006013074	0.173709375	6.34E-05	0.079268786	0.02474629	0.253918483	1.97E-05
SLC25A38	0.29130846	0.183314335	0.462924073	1.79834E-07	0.285284128	0.176905364	0.460059729	2.68E-07	0.301269657	0.193989945	0.467876858	9.20E-08
SLC38A6	3.899209282	2.150103987	7.071208237	7.4459E-06	3.879324932	2.09965325	7.167451067	1.50E-05	3.212941837	1.921519127	5.372309388	8.58E-06
SLC41A3	0.143503363	0.056515368	0.364382574	4.4388E-05	0.134752181	0.050279847	0.361141714	6.75E-05	0.161940979	0.071891905	0.364782106	1.11E-05
SLC44A3	0.331299498	0.209866435	0.522996244	2.11005E-06	0.296433708	0.178069631	0.493475179	2.92E-06	0.477618431	0.34161111	0.667775021	1.55E-05

SLC44A3-AS1	0.085069158	0.026176656	0.276458602	4.16686E-05	0.062099041	0.016238337	0.237480662	4.89E-05	0.193046953	0.090172783	0.413285748	2.28E-05
SLC45A2	3.025625218	1.855681115	4.933179459	9.05196E-06	3.062746636	1.839274755	5.100062908	1.69E-05	2.559356994	1.615262015	4.055260486	6.28E-05
SLC46A1	18.87883639	4.963956436	71.79967592	1.62707E-05	26.07426991	6.030766	112.7332003	1.27E-05	14.86307678	4.568393028	48.35640234	7.33E-06
SLCO3A1	7.098924946	2.701656511	18.65327261	6.99954E-05	8.255649439	2.96450347	22.99061153	5.35E-05	7.217420424	2.881171156	18.07985529	2.46E-05
SLCO5A1	2.820189036	1.826117049	4.355397813	2.93009E-06	3.018015672	1.926619281	4.727669181	1.41E-06	3.452286643	2.235938089	5.330327852	2.26E-08
SMARCD3	12.07044035	4.115886442	35.39833577	5.69555E-06	14.54544184	4.610769284	45.88602582	4.94E-06	21.035194	7.350168923	60.19989354	1.36E-08
SMIM10L2A	0.102921223	0.036039839	0.293918575	2.16646E-05	0.102579481	0.03464538	0.30372159	3.93E-05	0.10451361	0.040798864	0.267730364	2.53E-06
SNRPG	9.740679958	3.744317391	25.33995817	3.06406E-06	9.442120373	3.532693365	25.23673241	7.60E-06	6.896973577	3.068189448	15.50368559	2.97E-06
SNRPGP2	6.6766565	3.01315428	14.79437754	2.90994E-06	6.839683804	3.015721892	15.51246309	4.19E-06	5.53593367	2.62128047	11.69144696	7.24E-06
SOBP	0.192932145	0.094894425	0.392254997	5.49553E-06	0.18073461	0.085672371	0.381278107	7.07E-06	0.28836322	0.161497993	0.514887802	2.62E-05
SPAG1	22.09524715	5.781393927	84.44329389	6.03845E-06	25.51155971	6.168545995	105.5094149	7.75E-06	13.37640065	4.133321316	43.28918094	1.50E-05
SPON2	2.442152235	1.619557444	3.682553874	2.03724E-05	2.573046721	1.659672905	3.989080864	2.39E-05	2.336039016	1.61740735	3.373966542	6.09E-06
SQLE	2.000467306	1.425076589	2.808178503	6.149E-05	2.152734849	1.513998185	3.060946425	1.96E-05	2.251195003	1.634728034	3.100135826	6.68E-07
STPG1	0.057294975	0.015203953	0.215911891	2.39363E-05	0.044175684	0.010524814	0.185418101	2.02E-05	0.043102048	0.011930745	0.155714205	1.60E-06
SULF2	2.277670517	1.570987523	3.302243278	1.40294E-05	2.470488856	1.651337743	3.695982371	1.08E-05	1.946437353	1.415980349	2.675615076	4.09E-05
TARS2	13.95039254	3.755344772	51.82305853	8.2804E-05	17.82360202	4.548436832	69.84394871	3.57E-05	10.04582406	3.463893716	29.13443347	2.17E-05
TATDN2	0.034882995	0.006728506	0.180845985	6.42306E-05	0.019771615	0.003088428	0.126574672	3.44E-05	0.040163876	0.009077218	0.177712707	2.27E-05
TCTN1	0.07434878	0.025460247	0.217112629	2.00073E-06	0.064844061	0.020611593	0.203999383	2.89E-06	0.165680866	0.071790704	0.382363563	2.52E-05
TFAP2A	0.275182827	0.151206799	0.500808093	2.40534E-05	0.22726996	0.112739468	0.458150424	3.44E-05	0.354375187	0.220165189	0.570397951	1.94E-05
TIMM23	10.96810453	3.617202219	33.25755921	2.32014E-05	10.37894236	3.285334056	32.78888618	6.70E-05	8.42794822	3.148762999	22.5581637	2.20E-05
TLCD1	2.870128451	1.714513834	4.804649087	6.04994E-05	3.121143679	1.796868115	5.421398367	5.34E-05	2.530394734	1.62979696	3.928647351	3.53E-05
TLL2	1247370232	61521.19661	2.5291E+13	3.48374E-05	1124509709	40727.76645	3.10482E+13	6.48E-05	11008351655	696563.4562	1.73974E+14	2.77E-06

TMEM161A	4.150048834	2.030767763	8.480982237	9.51364E-05	4.644419499	2.221654481	9.709265175	4.47E-05	3.532977317	1.892665121	6.594895517	7.39E-05
TMEM191A	23.64843564	5.42689188	103.0513451	2.52985E-05	20.85731869	4.553719343	95.53240114	9.14E-05	17.05757955	4.488341714	64.825951	3.12E-05
TMEM255A	2.364237829	1.633323549	3.42223714	5.11553E-06	2.443472978	1.660351686	3.595961172	5.85E-06	2.100148389	1.532803538	2.877487653	3.87E-06
TNFAIP8L3	18.82422413	5.684385414	62.33768266	1.5529E-06	19.80529305	5.930025393	66.14636645	1.22E-06	12.01947528	3.834841211	37.67242974	1.99E-05
TNFRSF19	2.213458164	1.554510934	3.151728905	1.04964E-05	2.397496382	1.630954606	3.524309555	8.65E-06	2.073358928	1.526729646	2.815703001	3.02E-06
TNK2	0.086270564	0.025808735	0.288375636	6.90431E-05	0.062585602	0.016367521	0.239312817	5.13E-05	0.070867369	0.020602848	0.243761645	2.68E-05
TRIB1	2.840031896	1.764626511	4.570814912	1.71492E-05	2.881701231	1.760822774	4.716091881	2.54E-05	2.859320106	1.827776527	4.473036693	4.19E-06
TRPM4	5.236185407	2.436303659	11.25378502	2.2244E-05	4.949750541	2.255552208	10.86209857	6.65E-05	4.057092144	2.096988786	7.849348919	3.19E-05
TTYH3	2.490225909	1.602587202	3.869508674	4.96686E-05	2.756463677	1.737111404	4.373980842	1.68E-05	2.400013676	1.629111292	3.535710342	9.48E-06
TUBA8	7.574354296	2.768298252	20.72422759	8.05675E-05	8.537357066	3.031380804	24.04398206	4.92E-05	8.823297885	3.224429354	24.1439886	2.24E-05
ULBP1	17.85860918	5.997861222	53.1739415	2.24342E-07	20.04511703	6.594942073	60.92649675	1.25E-07	8.91688757	3.212980292	24.74676989	2.66E-05
UQCRQ	14.45218763	4.768882408	43.79762583	2.34242E-06	12.35649113	3.951435274	38.63985173	1.55E-05	7.044367162	2.743513012	18.08743333	4.96E-05
VGF	1.57566401	1.293896283	1.918791408	6.0919E-06	1.567194634	1.278800295	1.920627505	1.49E-05	1.517618838	1.269476236	1.814265499	4.66E-06
VIM	0.285432484	0.161152583	0.505556296	1.7189E-05	0.264454178	0.145409856	0.480957852	1.31E-05	0.369890668	0.225080564	0.607867262	8.71E-05
WARS1	1.649120702	1.315183167	2.06784816	1.46958E-05	1.651609414	1.306701126	2.087557441	2.69E-05	1.492318022	1.220864058	1.824128628	9.30E-05
Z97192.2	6.193445787	2.582464252	14.85355342	4.39394E-05	6.462201091	2.691613457	15.51487374	2.97E-05	8.167067573	3.156234895	21.13308894	1.49E-05
ZC3HC1	38.79178792	7.533071529	199.7595276	1.21519E-05	41.35937758	7.530104325	227.167917	1.84E-05	34.36112973	8.685225745	135.942032	4.64E-07
ZHX1-C8orf76	7.662398899	3.037406839	19.32976384	1.60885E-05	7.425305137	2.878707692	19.1527457	3.37E-05	5.393959901	2.477396423	11.74410488	2.18E-05
ZNF580	0.095107678	0.032524195	0.278115116	1.72757E-05	0.079480134	0.025487475	0.247850826	1.28E-05	0.140217819	0.057562048	0.341562494	1.53E-05
ZNF667-AS1	0.312539377	0.183767432	0.531546101	1.76801E-05	0.295827111	0.167281464	0.523152282	2.82E-05	0.422671689	0.289025867	0.618115462	8.96E-06
ZSCAN18	0.223822875	0.12430539	0.403012929	6.08147E-07	0.217650939	0.118864763	0.398536369	7.78E-07	0.220007447	0.122861423	0.393966436	3.51E-07

Table S3. One hundred and ten genes were significantly predicting prognosis of UM patients by both Kaplan-Meier and univariate Cox regression analyses (p-value < 0.0001).

Gene	Kaplan-Meier analysis (p-value)			Univariate Cox analysis (overall survival)				Univariate Cox analysis (disease-specific survival)				Univariate Cox analysis (progression-free survival)			
	Overall survival	Disease-specific survival	Progression-free survival	HR	HR.95L	HR.95H	coxPvalue	HR	HR.95L	HR.95H	coxPvalue	HR	HR.95L	HR.95H	coxPvalue
AC016747.1	2.92813E-05	2.99366E-05	1.78204E-05	9.544636894	3.554714474	25.62796369	7.58125E-06	11.96299747	4.206174969	34.0245733	3.26142E-06	10.87188383	4.385472264	26.95213899	2.5861E-07
AC018529.1	7.99098E-07	7.10228E-07	1.03762E-06	2.102398694	1.480720952	2.985086598	3.25802E-05	2.123047469	1.474190721	3.057494863	5.22149E-05	1.893454562	1.403847671	2.553817093	2.88742E-05
AC104129.1	2.51321E-06	2.31727E-06	2.97215E-05	2.432702647	1.711007626	3.458805254	7.37731E-07	2.499191646	1.728450582	3.613617277	1.12382E-06	2.117821317	1.566893585	2.862458033	1.05331E-06
AC141557.1	1.12362E-08	8.4835E-09	4.61251E-06	0.597307929	0.481926737	0.740313276	2.53118E-06	0.569832404	0.449472075	0.722423009	3.38713E-06	0.666518957	0.559359412	0.794207643	5.72108E-06
ADAM11	3.92458E-06	4.22335E-06	2.40E-06	2.828831435	1.772500157	4.514689184	1.30171E-05	2.990540517	1.837011917	4.868412939	1.05351E-05	2.938309282	1.909997285	4.520248017	9.3678E-07
ADCK5	1.38931E-05	1.50443E-05	7.23578E-06	4.704347835	2.351146323	9.412807844	1.20993E-05	4.92389884	2.37741059	10.19797753	1.77704E-05	4.286668181	2.299196349	7.992150867	4.6628E-06
AIFM2	6.03304E-06	3.36675E-06	3.52585E-05	6.104896042	2.780109422	13.40585928	6.55369E-06	6.361446522	2.80693412	14.41715413	9.31851E-06	4.279461512	2.15375306	8.503198984	3.32485E-05
AL391422.4	1.00365E-05	1.08563E-06	9.55165E-05	0.172904546	0.072501444	0.412350156	7.56753E-05	0.147206889	0.057140807	0.379236299	7.24375E-05	0.137131483	0.052652228	0.357155701	4.7404E-05
AMN	9.20972E-05	6.28326E-05	2.94334E-05	2.886128018	1.827699871	4.557496047	5.43789E-06	2.945629612	1.834194313	4.730542316	7.83145E-06	2.385714216	1.580052175	3.602179987	3.53466E-05
ANPEP	3.46581E-05	2.08483E-05	4.48143E-05	4.620532952	2.47840601	8.614135322	1.466E-06	4.963064664	2.61522417	9.418699607	9.53752E-07	7.383191057	3.979498296	13.69808607	2.29926E-10
ANXA2P2	5.37849E-06	6.04906E-06	2.02506E-05	4.184992861	2.140278553	8.183124213	2.86354E-05	4.560062193	2.283751209	9.105268175	1.70351E-05	3.455892696	1.909752246	6.253792528	4.16856E-05
ARMC9	1.87936E-06	1.7964E-06	1.35557E-05	2.405804405	1.629651225	3.551615676	9.99386E-06	2.479827635	1.639939428	3.74986112	1.67402E-05	2.235996564	1.584180493	3.156004418	4.72786E-06
ATP8B3	5.99075E-05	9.14712E-06	5.03723E-05	12.38446188	4.430219505	34.6201573	1.60395E-06	15.15665119	5.13672052	44.72193384	8.47311E-07	12.65279839	4.969466613	32.2153904	1.02397E-07
AZGP1	1.4636E-05	1.41176E-05	1.9977E-05	0.52178064	0.380903837	0.71476055	5.09153E-05	0.49038853	0.345084771	0.696874887	7.05982E-05	0.569825832	0.433171409	0.749591205	5.81451E-05
BATF3	8.16694E-06	8.363E-06	1.48206E-06	10.56358957	3.694222511	30.20647083	1.09389E-05	9.024083513	3.019877732	26.96601998	8.1893E-05	7.172429408	2.676589426	19.21988599	8.94253E-05
CA12	1.55048E-06	8.03226E-07	2.287E-06	2.01276314	1.591150843	2.546091389	5.4496E-09	2.018250995	1.57970744	2.578538895	1.93249E-08	1.698605727	1.393223824	2.070924546	1.61007E-07
CALHM2	3.5304E-07	3.34105E-07	2.96011E-07	4.117429767	2.152813126	7.874918488	1.88955E-05	4.755004752	2.28914654	9.877074183	2.9096E-05	3.981665112	2.242158306	7.070712635	2.40801E-06
CAMK1	2.4783E-05	1.4885E-05	6.46984E-05	18.44935557	4.308533773	79.00105671	8.55709E-05	29.25362659	5.507929638	155.3713873	7.4131E-05	10.98762701	3.490152424	34.59102433	4.2005E-05

CAMSAP3	2.06594E-06	4.02545E-06	2.78571E-06	0.259407094	0.136053978	0.49459811	4.16571E-05	0.273317228	0.143625282	0.520119479	7.77552E-05	0.329846659	0.200265266	0.543273532	1.32133E-05
CARD11	5.30339E-08	4.53672E-08	6.24998E-07	2.36818971	1.611519222	3.480146204	1.13606E-05	2.409357064	1.614559764	3.595408229	1.66548E-05	2.02434702	1.472210484	2.783556361	1.42388E-05
CCDC188	6.72807E-05	2.97003E-05	2.18454E-05	5.510372264	2.431513571	12.48777833	4.34032E-05	5.663744617	2.427536651	13.21421989	6.02833E-05	8.721569745	3.90164595	19.49581786	1.31257E-07
CDCA7L	2.91938E-07	2.44845E-07	9.5946E-05	0.226652284	0.118199484	0.43461491	7.87347E-06	0.203877436	0.101153488	0.410920173	8.70821E-06	0.29642786	0.170653359	0.514900359	1.58807E-05
CDH24	1.27083E-06	1.18966E-06	6.97671E-06	4.389650126	2.177993741	8.847145821	3.52142E-05	5.315825586	2.492460292	11.33739291	1.53764E-05	5.850086669	2.919127422	11.72388495	6.34729E-07
CHAC1	5.74193E-07	4.99772E-07	7.76341E-07	1.6736408	1.325776266	2.11277996	1.4772E-05	1.684644586	1.3199957	2.150027748	2.7806E-05	1.540187285	1.2706961	1.866832576	1.07671E-05
COX6C	4.1074E-07	8.87026E-07	1.2856E-05	4.158652303	2.336451662	7.401988777	1.26721E-06	4.175167383	2.305926457	7.559661161	2.37885E-06	3.486756799	2.105321603	5.774639349	1.22115E-06
CTF1	3.47162E-05	3.74352E-05	4.66598E-05	0.293312332	0.171654034	0.50119489	7.22407E-06	0.271916874	0.151369173	0.48846661	1.31693E-05	0.389531524	0.263447872	0.575957615	2.3018E-06
CTNNBIP1	5.62886E-05	5.83725E-05	8.67676E-07	0.165208825	0.067071635	0.406937389	9.04748E-05	0.143256722	0.05440364	0.377226388	8.37192E-05	0.157216826	0.069547099	0.355401315	8.75175E-06
CYTOR	8.57349E-06	3.45601E-05	6.23247E-05	2.86156855	1.791223364	4.571498302	1.08919E-05	2.785846964	1.71929275	4.514032474	3.17363E-05	2.363132829	1.53266612	3.643583357	9.90527E-05
DLL4	5.00509E-07	4.57287E-07	5.13596E-07	2.724019653	1.751594322	4.236302308	8.67291E-06	2.756228609	1.740352311	4.365090963	1.54644E-05	2.284501673	1.590429882	3.281469969	7.77781E-06
DTYMK	6.08075E-06	2.79537E-05	1.42714E-05	14.59040461	4.090210293	52.04620094	3.61504E-05	12.98983537	3.579191102	47.14356348	9.66694E-05	10.82104349	3.741709903	31.29451112	1.10595E-05
EFS	9.45965E-08	2.05164E-07	1.24804E-05	0.320836024	0.191568038	0.537332612	1.55526E-05	0.310339901	0.179178018	0.5375149	2.97867E-05	0.467922649	0.320634193	0.682870419	8.22299E-05
ENPP2	1.3071E-06	1.18991E-06	2.51444E-06	0.40633097	0.271636135	0.607816253	1.1697E-05	0.3666603	0.230754637	0.582609203	2.17142E-05	0.523585694	0.38282532	0.716102004	5.11758E-05
FABP5	2.45994E-06	2.38009E-06	1.03265E-05	2.673537438	1.724188791	4.14560312	1.11256E-05	2.752291738	1.737723407	4.359214926	1.5953E-05	2.616302715	1.757340986	3.895111964	2.17224E-06
FKBP10	2.78285E-06	2.57396E-06	3.55938E-05	3.054512459	1.834061228	5.08709645	1.78262E-05	3.278884393	1.910350404	5.627806731	1.64447E-05	2.85594634	1.828345801	4.461097835	3.99333E-06
FKBP11	3.15723E-08	1.56312E-07	1.98036E-07	3.116112608	1.918888474	5.060303358	4.33488E-06	2.980144998	1.82212622	4.874121295	1.35929E-05	2.451636617	1.654121914	3.633663307	7.94096E-06
GEM	5.64978E-06	3.2929E-06	6.38637E-07	1.87586383	1.389308687	2.532817321	4.02145E-05	1.966458274	1.43273698	2.699000721	2.84277E-05	1.977036566	1.496828369	2.611303784	1.57795E-06
GLA	8.34545E-07	7.63463E-07	2.93763E-05	7.514212998	3.242221928	17.41503149	2.56665E-06	7.668835468	3.194100361	18.41239497	5.14741E-06	5.771688141	2.77746246	11.99381971	2.63595E-06
GREB1	3.24559E-05	3.20467E-05	3.0917E-05	2.918774613	1.740891206	4.893611511	4.85186E-05	3.166774359	1.821499016	5.505608156	4.40588E-05	3.331966745	2.018666148	5.499672347	2.51043E-06
GUSB	1.54565E-05	1.65246E-05	8.18847E-06	18.36767819	6.215019646	54.28327204	1.40628E-07	22.31865591	7.056475459	70.59082182	1.25134E-07	13.2658257	5.314336087	33.11460333	3.04398E-08

HDAC11	3.1133E-06	3.05715E-06	7.3848E-06	0.182527972	0.083645863	0.398303748	1.93596E-05	0.161368069	0.06879668	0.378501604	2.74666E-05	0.222351275	0.114395459	0.432185769	9.2516E-06
HM13	4.06989E-06	3.66898E-06	5.44794E-05	3.964653458	2.131816468	7.37327874	1.3538E-05	4.330410544	2.24914546	8.337591238	1.16003E-05	2.868018626	1.70295612	4.830148433	7.44174E-05
HMCES	1.16729E-08	2.68654E-08	6.59499E-05	0.023986894	0.00473697	0.121463946	6.5693E-06	0.016410146	0.002743987	0.098139296	6.67181E-06	0.088390242	0.027057148	0.288753086	5.90507E-05
HPSE	2.3184E-05	1.34117E-05	5.31255E-05	3.540317173	1.899875444	6.597193373	6.86572E-05	3.674569149	1.940454028	6.958401608	6.47351E-05	3.596314399	2.028708395	6.375227355	1.17749E-05
HTRA3	1.59045E-06	1.50497E-06	2.17282E-06	2.872887211	1.808824945	4.562896455	7.79173E-06	3.03051408	1.864086005	4.926819665	7.7599E-06	3.279124119	2.101553339	5.116527279	1.67906E-07
IDH2	3.94135E-05	4.08558E-05	6.12997E-05	5.348924811	2.73383751	10.46550738	9.74281E-07	5.448315346	2.711656319	10.94686665	1.91589E-06	3.91018165	2.260035415	6.765168561	1.08724E-06
IFT122	1.93891E-07	3.97769E-07	1.22603E-05	0.169308088	0.074004829	0.387342677	2.5976E-05	0.169920951	0.072340224	0.399129668	4.74178E-05	0.212191222	0.103390365	0.435486562	2.37771E-05
ISG20	3.08101E-07	2.64687E-07	1.99013E-05	3.335132325	2.108978589	5.274168114	2.58941E-07	3.131877749	1.966071993	4.988961882	1.5413E-06	2.351251904	1.600158654	3.454898364	1.33594E-05
ISM1	4.52659E-06	2.46168E-06	5.9253E-07	1.931502551	1.426377397	2.615508427	2.08145E-05	1.989694074	1.455351423	2.72022444	1.61993E-05	2.468388455	1.802290564	3.380665517	1.79279E-08
JAG2	9.07968E-06	1.14491E-06	1.17166E-05	4.209973418	2.089705945	8.481516848	5.76287E-05	4.725814245	2.215813469	10.07906152	5.85013E-05	4.284269159	2.221546821	8.262244151	1.41135E-05
JPH1	7.47602E-05	1.19121E-05	7.69843E-06	3.853672657	2.105842995	7.052184317	1.21263E-05	4.449912244	2.353082894	8.415223718	4.38458E-06	7.799475313	3.933259909	15.46600443	4.0846E-09
JUP	6.44106E-07	1.31927E-06	6.16642E-05	0.184288417	0.08332523	0.407586281	2.96527E-05	0.164110885	0.068793057	0.391498556	4.6218E-05	0.266426022	0.144127638	0.492499749	2.44997E-05
KCTD17	4.2681E-06	4.25012E-06	4.38985E-06	3.595316565	1.953629828	6.616556022	3.92393E-05	4.12071317	2.06051139	8.240807169	6.21665E-05	2.737888621	1.681379045	4.45826545	5.14963E-05
KDELR3	5.84681E-08	5.5396E-08	2.27676E-07	4.876110214	2.568753255	9.256027518	1.26639E-06	5.050643403	2.567633086	9.93483022	2.70727E-06	3.842710292	2.265965925	6.51661273	5.87106E-07
KDM7A-DT	8.73862E-06	8.23607E-06	9.96183E-06	5.902927507	2.605105397	13.37548692	2.09815E-05	6.863425936	2.835130034	16.61532805	1.95366E-05	4.474173249	2.231019941	8.972679216	2.44122E-05
LAMA1	1.96921E-05	1.17723E-05	2.89355E-05	4.106893616	2.226186536	7.576442897	6.14436E-06	4.235369847	2.269581935	7.903815878	5.76572E-06	3.800193372	2.175567695	6.638023584	2.71419E-06
LHFPL6	6.44431E-06	3.66779E-06	5.97788E-06	2.439071815	1.607967647	3.699745656	2.73722E-05	2.66660943	1.709587315	4.159369801	1.53087E-05	2.476312574	1.682959853	3.643654334	4.19134E-06
LINC00518	9.5023E-06	1.0963E-06	4.16616E-06	0.439960504	0.298074693	0.649385037	3.57569E-05	0.398240708	0.259966239	0.610062528	2.32669E-05	0.358462171	0.237489782	0.541055395	1.03885E-06
LMCD1	1.96384E-08	3.80705E-08	3.36323E-06	0.097639012	0.033671028	0.283132922	1.84419E-05	0.103977064	0.035712551	0.302729136	3.30345E-05	0.251113894	0.130253908	0.484117435	3.69174E-05
LTBR	7.87352E-08	1.80851E-07	6.79528E-06	6.738031046	2.873860262	15.79793665	1.14342E-05	7.429365184	3.041741857	18.14600635	1.07501E-05	5.595765352	2.72973614	11.47092183	2.577E-06
MALSU1	4.04806E-05	4.39392E-05	1.01792E-05	14.04696723	3.921299863	50.31935718	4.93155E-05	16.23259858	4.227249725	62.33302358	4.90955E-05	11.63003259	4.008633619	33.74158649	6.33614E-06

MANEAL	2.12535E-08	4.88443E-08	5.54137E-06	0.123166064	0.044418193	0.341524012	5.70788E-05	0.129231787	0.046106416	0.362224098	9.97939E-05	0.208660993	0.10159903	0.428541589	1.97517E-05
MAP4K3-DT	6.53445E-06	6.85397E-06	1.88346E-05	3.782114111	1.992772274	7.178134368	4.71934E-05	3.879468168	2.003662476	7.511381505	5.78181E-05	4.031488809	2.211284831	7.349981237	5.3689E-06
MAPK12	6.22319E-07	1.40976E-06	4.10909E-06	3.70200997	2.02866826	6.762222078	2.06251E-05	4.255790757	2.232640104	8.112259084	1.0813E-05	4.00923893	2.273528856	7.070065004	1.60461E-06
MIB2	4.03004E-06	3.81452E-06	1.35346E-09	0.223318848	0.107330036	0.464653792	6.06506E-05	0.208561224	0.097874907	0.444422227	4.88715E-05	0.198825825	0.102990005	0.383840244	1.48678E-06
MIRLET7BHG	8.10319E-05	4.82393E-05	1.13047E-05	76.63123502	8.852725565	663.3376509	8.13759E-05	102.9533688	10.9159986	970.9964744	5.17444E-05	89.34941324	12.63680746	631.75115	6.73819E-06
MTMR14	3.99571E-06	3.97081E-06	1.21905E-05	0.043425963	0.010267305	0.183671794	2.01593E-05	0.030535198	0.006040582	0.154355713	2.44257E-05	0.071131929	0.02218511	0.228069696	8.7305E-06
MYEOV	3.49545E-05	3.46366E-05	3.85719E-05	1.81571434	1.381862194	2.385779551	1.85556E-05	1.85945275	1.39704912	2.474905485	2.11873E-05	1.892893082	1.458729598	2.456277177	1.58449E-06
NICN1	3.24324E-07	2.89606E-07	5.42443E-06	0.131079324	0.057276181	0.299981402	1.50685E-06	0.128120608	0.054403458	0.301725124	2.57877E-06	0.177298632	0.083431345	0.376774518	6.86228E-06
NKX3-2	4.85557E-06	4.75471E-07	5.36416E-05	397886.1702	1479.01427	107039808.7	6.27243E-06	604561.0189	2192.096572	166732629.5	3.43523E-06	12781.22715	115.8501406	1410095.548	8.13834E-05
NQO1	1.21525E-05	1.21306E-05	9.57862E-06	2.073706277	1.518667571	2.831599097	4.45493E-06	2.147999724	1.542820836	2.99056294	5.95118E-06	1.76500575	1.373617181	2.267913755	8.92532E-06
ORAI2	2.29245E-06	1.30271E-06	4.99827E-05	25.55602115	6.133467885	106.4830255	8.54948E-06	28.78232336	6.34911155	130.4784349	1.32001E-05	9.217431625	3.041001184	27.93851124	8.64706E-05
ORMDL2	6.70856E-08	1.45531E-07	1.20085E-05	8.259971084	3.361937726	20.29398754	4.14991E-06	7.796422908	3.101008425	19.60143341	1.26587E-05	5.385156304	2.494630284	11.6249324	1.80031E-05
P2RX4	6.44115E-08	6.24478E-07	1.3324E-07	7.271488636	3.102655592	17.04170682	4.98183E-06	6.769054923	2.816990258	16.26562408	1.90845E-05	11.62411172	4.511753659	29.94843768	3.76886E-07
PACSIN3	6.36936E-05	9.11656E-06	6.52444E-05	0.168248118	0.068743313	0.411784476	9.50719E-05	0.140772348	0.053987415	0.367064322	6.08339E-05	0.192919893	0.091380118	0.407288654	1.58917E-05
PLCD1	4.94688E-06	4.9641E-06	1.10003E-05	0.146407392	0.063242618	0.338934807	7.24972E-06	0.135718292	0.055430173	0.332300154	1.23461E-05	0.201649038	0.101571768	0.400331072	4.7298E-06
PLEKHG4B	8.78296E-06	5.56309E-06	1.35151E-07	3.202669842	2.026722152	5.060927621	6.16776E-07	3.542060327	2.180462849	5.753912003	3.23718E-07	2.614894279	1.788428177	3.82328582	7.07549E-07
PLXNB1	1.22158E-06	1.10923E-06	3.40621E-06	0.256626343	0.136786166	0.48146009	2.26712E-05	0.216162562	0.105050798	0.444796747	3.17622E-05	0.295011229	0.172657471	0.504071007	7.95864E-06
POMGNT2	9.84951E-07	2.11412E-06	1.29919E-05	0.112116581	0.043802333	0.286973933	5.03565E-06	0.11811045	0.045838836	0.304328812	9.71356E-06	0.225801197	0.114982291	0.443426378	1.54823E-05
POMP	1.37201E-06	1.31936E-06	8.70432E-05	8.417334195	3.35777829	21.10071269	5.53966E-06	7.870803632	3.065611737	20.20789165	1.79852E-05	5.513117272	2.371736346	12.8152786	7.28851E-05
PPM1K	9.01414E-05	5.90266E-05	2.58623E-05	2.49755248	1.625994631	3.836278591	2.91639E-05	2.649150934	1.66841245	4.206394333	3.63106E-05	2.061360998	1.43885339	2.953191197	8.02959E-05
PRKCD	1.2651E-06	1.15009E-06	2.37191E-05	0.265343444	0.13933301	0.505315597	5.41831E-05	0.240898009	0.120468108	0.481719617	5.68017E-05	0.309185127	0.1762561	0.542366719	4.24651E-05

RAB31	3.63771E-05	4.04724E-05	9.32682E-05	2.718533409	1.730868643	4.269777447	1.41382E-05	2.869657496	1.760531318	4.677527778	2.34759E-05	2.447895859	1.632743574	3.670015447	1.47266E-05
RBP7	5.98991E-05	3.69388E-05	7.75629E-06	0.446182461	0.301614807	0.660043154	5.35838E-05	0.392213747	0.256573811	0.599560893	1.54277E-05	0.464605758	0.329221175	0.655664117	1.29033E-05
RHOF	1.35505E-05	8.07849E-06	2.45529E-07	809.9834223	36.08349591	18182.08375	2.45442E-05	941.0875938	37.71606503	23481.92629	3.02421E-05	2668.792665	132.7764715	53642.44292	2.56251E-07
RNF208	2.48609E-06	5.05941E-06	2.31401E-05	0.213242569	0.110087647	0.413056274	4.62634E-06	0.222645459	0.114445059	0.433142336	9.68232E-06	0.377361562	0.240400892	0.592351165	2.27333E-05
RRM2	3.79462E-06	2.93847E-05	4.64713E-06	4.050201835	2.06275514	7.952536191	4.84066E-05	4.119682254	2.062055515	8.230516469	6.08458E-05	5.177421323	2.795436035	9.589091367	1.70354E-07
SFXN3	5.0624E-06	5.50825E-06	2.11683E-06	5.245920112	2.397417122	11.478886	3.34615E-05	5.840572509	2.465374388	13.83655456	6.0589E-05	4.265772155	2.202891052	8.260423077	1.69054E-05
SIRT3	1.92613E-06	3.77071E-07	3.56333E-05	0.012583232	0.002763116	0.057304049	1.54302E-08	0.010349122	0.002124683	0.050409558	1.52857E-08	0.062824277	0.018978569	0.207965618	5.86395E-06
SLC1A1	8.0637E-06	2.60093E-05	1.47548E-05	2.730951008	1.688969339	4.415766014	4.1726E-05	2.893127358	1.758939316	4.758655307	2.8624E-05	3.068887023	1.938498274	4.858434845	1.71914E-06
SLC25A26	4.86394E-06	4.8727E-06	1.18861E-05	0.048806751	0.011549927	0.206243633	4.00947E-05	0.032319147	0.006013074	0.173709375	6.33603E-05	0.079268786	0.02474629	0.253918483	1.97491E-05
SLC25A38	5.32199E-07	4.87755E-07	1.20319E-06	0.29130846	0.183314335	0.462924073	1.79834E-07	0.285284128	0.176905364	0.460059729	2.68481E-07	0.301269657	0.193989945	0.467876858	9.20181E-08
SLC41A3	7.41212E-07	1.59408E-06	8.60188E-06	0.143503363	0.056515368	0.364382574	4.4388E-05	0.134752181	0.050279847	0.361141714	6.75245E-05	0.161940979	0.071891905	0.364782106	1.11327E-05
SLC44A3	6.342E-07	5.53252E-07	5.96738E-05	0.331299498	0.209866435	0.522996244	2.11005E-06	0.296433708	0.178069631	0.493475179	2.92346E-06	0.477618431	0.34161111	0.667775021	1.54976E-05
SLC44A3-AS1	1.50032E-06	7.61193E-07	4.93288E-07	0.085069158	0.026176656	0.276458602	4.16686E-05	0.062099041	0.016238337	0.237480662	4.89343E-05	0.193046953	0.090172783	0.413285748	2.28438E-05
SLC45A2	7.08406E-07	1.71591E-06	9.75193E-05	3.025625218	1.855681115	4.933179459	9.05196E-06	3.062746636	1.839274755	5.100062908	1.69194E-05	2.559356994	1.615262015	4.055260486	6.28485E-05
SLC46A1	2.92672E-07	2.82805E-07	6.64835E-06	18.87883639	4.963956436	71.79967592	1.62707E-05	26.07426991	6.030766	112.7332003	1.26863E-05	14.86307678	4.568393028	48.35640234	7.33004E-06
SLCO3A1	6.75834E-07	6.46153E-07	3.16903E-06	7.098924946	2.701656511	18.65327261	6.99954E-05	8.255649439	2.96450347	22.99061153	5.35485E-05	7.217420424	2.881171156	18.07985529	2.45918E-05
SMARCD3	4.81531E-06	4.81766E-06	5.80594E-07	12.07044035	4.115886442	35.39833577	5.69555E-06	14.54544184	4.610769284	45.88602582	4.93894E-06	21.035194	7.350168923	60.19989354	1.36151E-08
SMIM10L2A	5.37578E-07	1.10248E-06	3.26355E-06	0.102921223	0.036039839	0.293918575	2.16646E-05	0.102579481	0.03464538	0.30372159	3.92872E-05	0.10451361	0.040798864	0.267730364	2.53024E-06
SNRPG	4.50672E-06	4.50223E-06	3.42973E-06	9.740679958	3.744317391	25.33995817	3.06406E-06	9.442120373	3.532693365	25.23673241	7.60444E-06	6.896973577	3.068189448	15.50368559	2.97286E-06
SOBP	1.62672E-09	3.28154E-09	1.77893E-05	0.192932145	0.094894425	0.392254997	5.49553E-06	0.18073461	0.085672371	0.381278107	7.06972E-06	0.28836322	0.161497993	0.514887802	2.62028E-05
STPG1	1.79625E-05	4.45702E-06	7.91245E-06	0.057294975	0.015203953	0.215911891	2.39363E-05	0.044175684	0.010524814	0.185418101	2.02171E-05	0.043102048	0.011930745	0.155714205	1.60451E-06

SULF2	9.53762E-08	7.93705E-08	1.1886E-05	2.277670517	1.570987523	3.302243278	1.40294E-05	2.470488856	1.651337743	3.695982371	1.08041E-05	1.946437353	1.415980349	2.675615076	4.08656E-05
TATDN2	1.31794E-06	1.16928E-07	3.20894E-06	0.034882995	0.006728506	0.180845985	6.42306E-05	0.019771615	0.003088428	0.126574672	3.44324E-05	0.040163876	0.009077218	0.177712707	2.26798E-05
TCTN1	3.25875E-10	6.924E-10	2.06358E-06	0.07434878	0.025460247	0.217112629	2.00073E-06	0.064844061	0.020611593	0.203999383	2.89186E-06	0.165680866	0.071790704	0.382363563	2.51943E-05
TFAP2A	3.86901E-05	4.19081E-05	4.69462E-05	0.275182827	0.151206799	0.500808093	2.40534E-05	0.22726996	0.112739468	0.458150424	3.44014E-05	0.354375187	0.220165189	0.570397951	1.93935E-05
TIMM23	3.43729E-05	3.70598E-05	9.23171E-05	10.96810453	3.617202219	33.25755921	2.32014E-05	10.37894236	3.285334056	32.78888618	6.70136E-05	8.42794822	3.148762999	22.5581637	2.20203E-05
TRPM4	2.21431E-06	1.04606E-05	1.92902E-05	5.236185407	2.436303659	11.25378502	2.2244E-05	4.949750541	2.255552208	10.86209857	6.65237E-05	4.057092144	2.096988786	7.849348919	3.19489E-05
ULBP1	6.33443E-06	6.61799E-06	2.89419E-05	17.85860918	5.997861222	53.1739415	2.24342E-07	20.04511703	6.594942073	60.92649675	1.25272E-07	8.91688757	3.212980292	24.74676989	2.65584E-05
ZNF667-AS1	1.45113E-07	1.23606E-07	4.85031E-06	0.312539377	0.183767432	0.531546101	1.76801E-05	0.295827111	0.167281464	0.523152282	2.82231E-05	0.422671689	0.289025867	0.618115462	8.96421E-06

Table S4. The status of chromosome copy number aberrations of TCGA-UVM cases.

Patient ID	3 CN (ABSOLUTE)	8q CN (ABSOLUTE)	1p CN (ABSOLUTE)	6q CN (ABSOLUTE)	6p CN (ABSOLUTE)
TCGA-V3-A9ZY	2	2	2	3	3
TCGA-V4-A9EC	2	2	2	2	2
TCGA-V4-A9EH	2	2	2	2	2
TCGA-V4-A9EY	2	2	2	2	3 (less than half)
TCGA-V4-A9F7	2	2	2	2	3
TCGA-VD-A8KE	2	2	2	3	3
TCGA-VD-A8KO	2	2	2	2	3
TCGA-VD-AA8M	2	2	2	2	2
TCGA-VD-AA8Q	2	2	2	2	2
TCGA-VD-AA8R	2	2	2	2	2
TCGA-WC-A87T	2	2	2	2	3 (less than half)
TCGA-WC-A87U	2	2	2	2	3
TCGA-WC-A880	2	2	2	2	3
TCGA-WC-A884	2	2	2	2	3
TCGA-YZ-A983	2	2	2	2	3
TCGA-V4-A9E5	2	3	2	3	4
TCGA-V4-A9E9	2	3	2	1	3
TCGA-V4-A9EA	2	3	2	2	3
TCGA-V4-A9EJ	2	3	2	2	4
TCGA-V4-A9EK	2	3	2	2	4
TCGA-V4-A9EM	2	4	2	3	3
TCGA-V4-A9ET	2	4	2	1	4
TCGA-V4-A9EW	2	2	2	1	4
TCGA-V4-A9EZ	2	3	1	1	3
TCGA-V4-A9F2	2	3	2	3	3
TCGA-V4-A9F4	2	3	2	1	4
TCGA-VD-A8K7	2	2	2	2	4
TCGA-VD-A8K9	2	3	2	1	5
TCGA-VD-A8KA	2	3	1	2	4
TCGA-VD-A8KB	2	4	2	1	4
TCGA-VD-A8KG	2	4	2	2	4
TCGA-VD-A8KJ	2	4	2	chromothripsis	3 and 4 (roughly half)
TCGA-VD-AA8S	2	3	2	2	4
TCGA-WC-A87W	2	2	1	2	3
TCGA-WC-A881	2	5	1 (partial, telomeric, ~72%)	1	3
TCGA-WC-A885	2	3	2	2	4
TCGA-WC-AA9E	2	3	1	1	5
TCGA-YZ-A982	3	4	2 (no LOH)	3	5
TCGA-V4-A9E8	1	3	2	1	3
TCGA-V4-A9ED	1	2	2	2	2
TCGA-V4-A9EF	1	4	2	2	2
TCGA-V4-A9EO	2, LOH	5	2 (no LOH)	4	4
TCGA-V4-A9EQ	1	3	1	2	2
TCGA-V4-A9ES	1	2	1	2	2
TCGA-V4-A9F0	1	3	2	1	2
TCGA-V4-A9F8	1	4	1	2	2
TCGA-VD-A8KD	1	3	2 (focal del)	2	2
TCGA-VD-A8KF	1	3	2	2	2

TCGA-VD-A8KH	1	3	1 (partial, telomeric, ~80%)	2	2
TCGA-VD-A8KK	1	3	2	2	2
TCGA-VD-A8KL	2, LOH	5	3	3	4
TCGA-VD-AA8O	2, LOH	4	2 (LOH)	3	3
TCGA-VD-AA8P	1	3	1	2	2
TCGA-VD-AA8T	1	3	1 (partial, telomeric, ~56%)	2	2
TCGA-WC-A882	1	3	1	2	2
TCGA-WC-A888	2, Subclonal LOH	3	2	2	2
TCGA-WC-AA9A	1	3	1	2	2
TCGA-YZ-A980	1	3	2	2	2
TCGA-YZ-A984	2, LOH	4	3	3	3
TCGA-YZ-A985	1	2	2	2	2
TCGA-RZ-AB0B	1	7	1	2	2
TCGA-V3-A9ZX	1	7	2	1	3
TCGA-V4-A9E7	1	5	1	1	2
TCGA-V4-A9EE	1	5	1	2	2
TCGA-V4-A9EI	1	8	2	1	3
TCGA-V4-A9EL	1	7	2	1	2
TCGA-V4-A9EU	1	6	2	1	2
TCGA-V4-A9EV	1	5	2	2	2
TCGA-V4-A9EX	1	6	2 (focal del)	1	3
TCGA-V4-A9F1	1	5	2	2	2
TCGA-V4-A9F3	1	5	2	1	3
TCGA-V4-A9F5	1	4	1	1	2
TCGA-VD-A8K8	1	5	2	2	2
TCGA-VD-A8KI	1	5	2	1	3
TCGA-VD-A8KM	1	3	2	2	3
TCGA-VD-A8KN	1	4	2	2	2
TCGA-VD-AA8N	1	4	2	1	2
TCGA-WC-A87Y	1	4	2	2	2
TCGA-WC-A883	1	6	1	2	2
TCGA-WC-A88A	1	4	2	1	3

Table S5. Enriched gene sets in HALLMARK collection (| NES |> 1, NOM p-val <0.05, and FDR q-val <0.25).

NAME	SIZE	ES	NES	NOM p-val	FDR q-val
HALLMARK_IL6_JAK_STAT3_SIGNALING	87	0.754572	1.672641	0.001996	0.097509
HALLMARK_REACTIVE_OXYGEN_SPECIES_PATHWAY	49	0.605997	1.58458	0.011561	0.182919
HALLMARK_ALLOGRAFT_REJECTION	200	0.714545	1.580144	0.029297	0.129023
HALLMARK_GLYCOLYSIS	200	0.526496	1.512575	0.034483	0.221163
HALLMARK_INFLAMMATORY_RESPONSE	200	0.63757	1.505531	0.046185	0.195025
HALLMARK_NOTCH_SIGNALING	32	0.603985	1.478482	0.038911	0.216515

Table S6. Enriched gene sets in KEGG collection (| NES |> 1, NOM p-val <0.05, and FDR q-val <0.25).

NAME	SIZE	ES	NES	NOM p-val	FDR q-val
KEGG_ALLOGRAFT_REJECTION	35	0.835471	1.471749	0.030593	0.212558
KEGG_AUTOIMMUNE_THYROID_DISEASE	50	0.775029	1.496166	0.041905	0.200204
KEGG_LEISHMANIA_INFECTION	69	0.695745	1.500718	0.040462	0.203374
KEGG_PROTEASOME	46	0.655372	1.509681	0.016227	0.232846
KEGG_NATURAL_KILLER_CELL_MEDIATED_CYTOTOXICITY	132	0.648655	1.524769	0.038536	0.23371
KEGG_CYTOSOLIC_DNA_SENSING_PATHWAY	54	0.618769	1.506395	0.048638	0.221757
KEGG_P53_SIGNALING_PATHWAY	68	0.545765	1.45541	0.039448	0.235732
KEGG_GLYCEROLIPID_METABOLISM	49	0.542718	1.529276	0.018868	0.245163

Table S7. Correlations of risk score with 20 kinds of TICs.

TICs	R	P-value
Monocytes	-0.52088	0.001126
Mast cells resting	-0.49575	0.002379
T cells CD4 memory activated	0.447794	0.00617
Eosinophils	-0.39353	0.017569
Macrophages M2	-0.31248	0.063955
B cells naive	-0.29494	0.080766
T cells CD8	0.287773	0.088975
Mast cells activated	0.284747	0.092329
Plasma cells	-0.2834	0.094108
Macrophages M1	0.22471	0.187006
Macrophages M0	0.223025	0.191054
Dendritic cells resting	0.211249	0.21617
T cells follicular helper	0.174823	0.307824
T cells gamma delta	-0.11278	0.51252
T cells regulatory (Tregs)	-0.08711	0.613421
Dendritic cells activated	0.024407	0.887638
B cells memory	-0.02049	0.905592
NK cells resting	0.016265	0.924989
T cells CD4 memory resting	0.012358	0.942971
NK cells activated	-0.0103	0.952817
