# Supplementary material for: Prognostic Implications of Novel Ten-Gene Signature in Uveal Melanoma
Source: Front Oncol. 2020 Oct 30;10:567512. doi: 10.3389/fonc.2020.567512 (PMC7661968; doi:10.3389/fonc.2020.567512)
Supplement: Supplementary file 2 [file Data_Sheet_2.PDF]

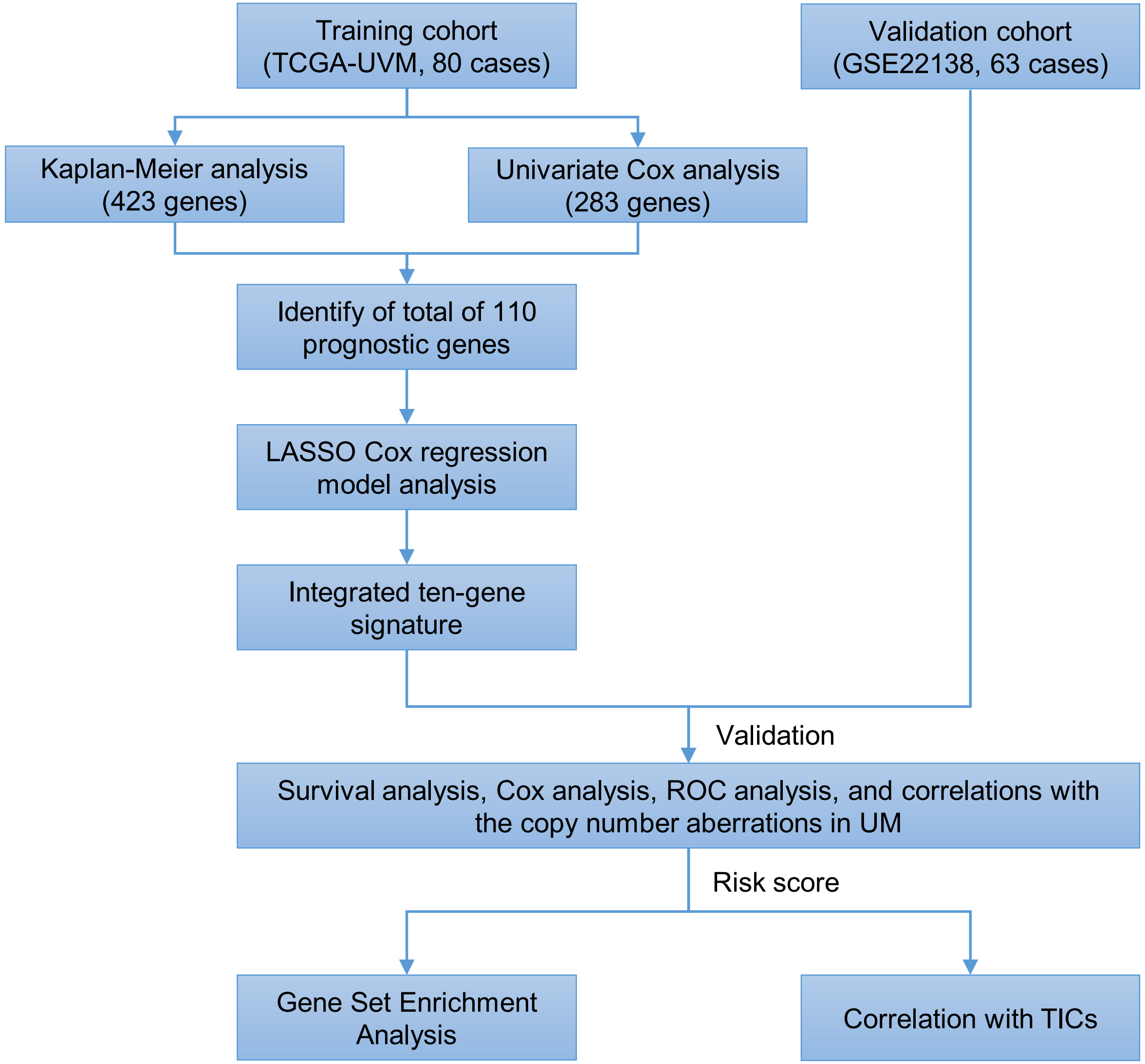
Training cohort
(TCGA-UVM, 80 cases)
Validation cohort
(GSE22138, 63 cases)
Kaplan-Meier analysis
(423 genes)
Univariate Cox analysis
(283 genes)
Identify of total of 110
prognostic genes
LASSO Cox regression
model analysis
Integrated ten-gene
signature
Validation
Survival analysis, Cox analysis, ROC analysis, and correlations with
the copy number aberrations in UM
Risk score
Gene Set Enrichment
Analysis
Correlation with TICs

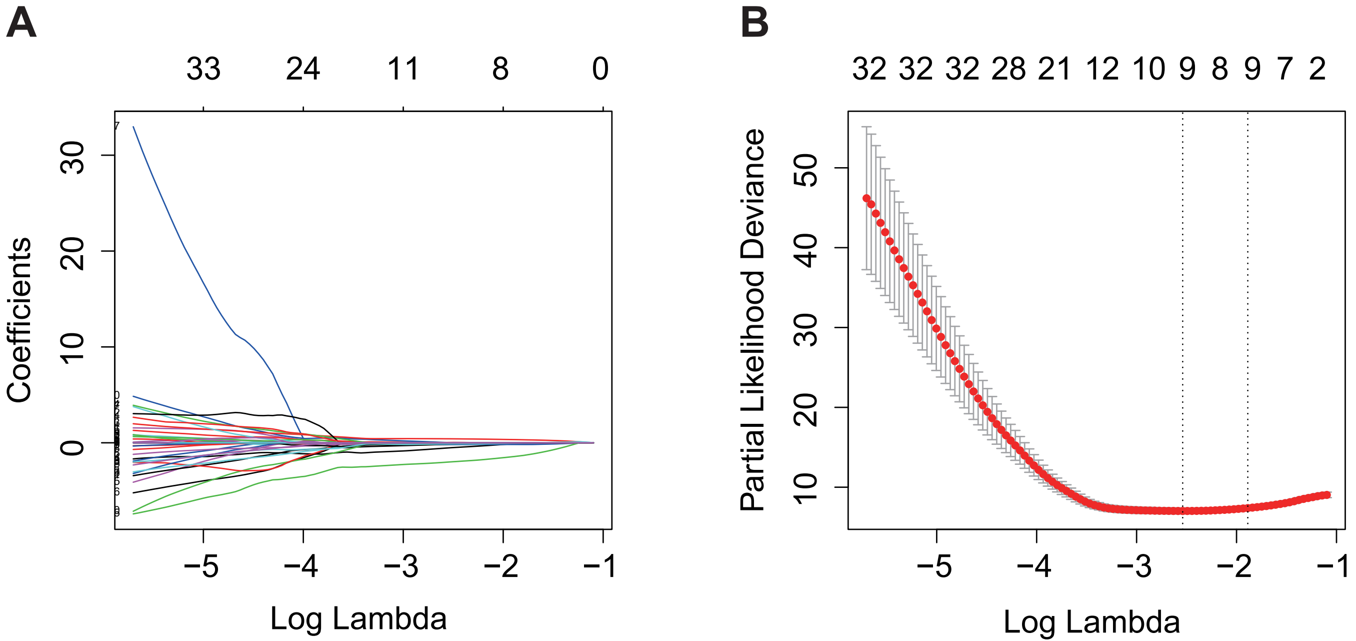
A
33 24 11 8 0
Coefficients
Log Lambda
B
32 32 32 28 21 12 10 9 8 9 7 2
Partial Likelihood Deviance
Log Lambda

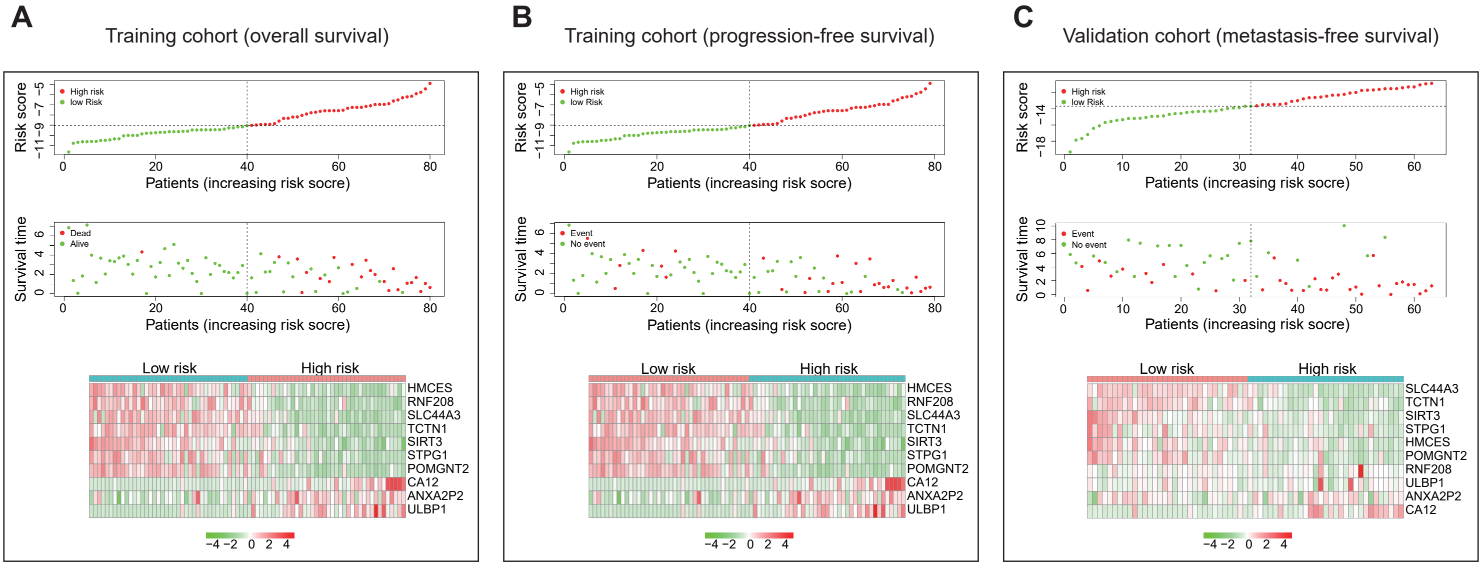

A
Training cohort (overall survival)
B
Training cohort (progression-free survival)
C
Validation cohort (metastasis-free survival)
Risk score
High risk
low Risk
Patients (increasing risk socre)
Survival time
Dead
Alive
Event
No event
Low risk
High risk
HMCES
RNF208
SLC44A3
TCTN1
SIRT3
STPG1
POMGNT2
CA12
ANXA2P2
ULBP1

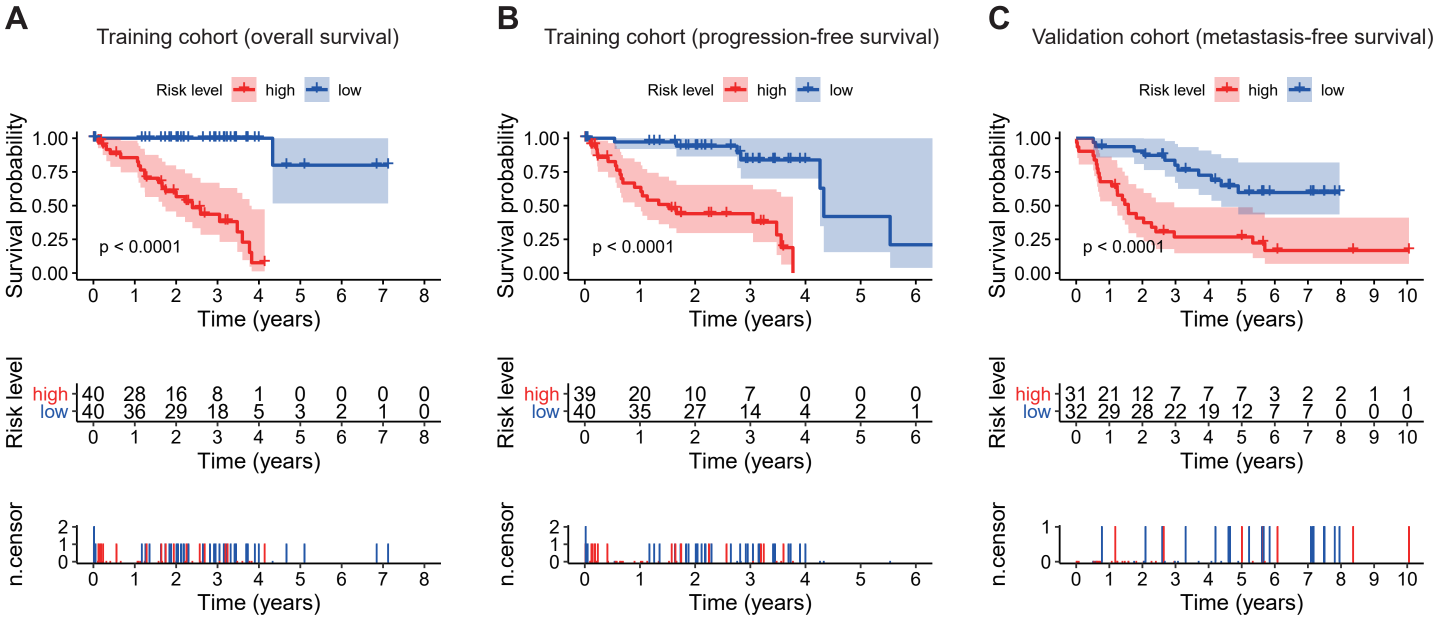

A
Training cohort (overall survival)
B
Training cohort (progression-free survival)
C
Validation cohort (metastasis-free survival)
Risk level
high
low
Survival probability
1.00
0.75
0.50
0.25
0.00
p < 0.0001
Time (years)
Risk level
high 40 28 16 8 1 0 0 0 0
low 40 36 29 18 5 3 2 1 0
high 39 20 10 7 0 0 0
low 40 35 27 14 4 2 1
high 31 21 12 7 7 7 3 2 2 1 1
low 32 29 28 22 19 12 7 7 0 0 0
n.censor

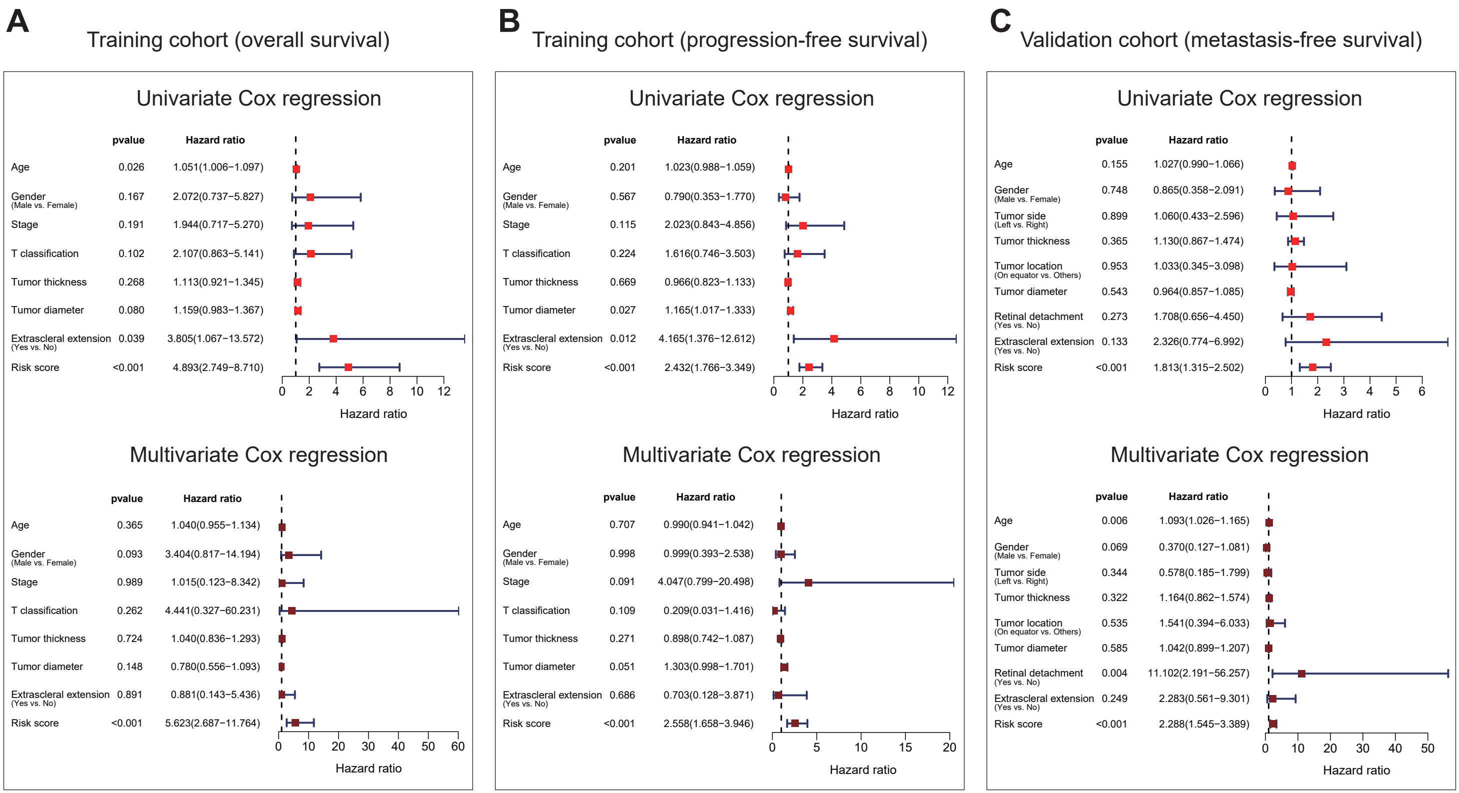
A
Training cohort (overall survival)
Univariate Cox regression
pvalue
Hazard ratio
Age 0.026 1.051(1.006−1.097)
Gender (Male vs. Female) 0.167 2.072(0.737−5.827)
Stage 0.191 1.944(0.717−5.270)
T classification 0.102 2.107(0.863−5.141)
Tumor thickness 0.268 1.113(0.921−1.345)
Tumor diameter 0.080 1.159(0.983−1.367)
Extrascleral extension (Yes vs. No) 0.039 3.805(1.067−13.572)
Risk score <0.001 4.893(2.749−8.710)
Hazard ratio
Multivariate Cox regression
pvalue
Hazard ratio
Age 0.365 1.040(0.955−1.134)
Gender (Male vs. Female) 0.093 3.404(0.817−14.194)
Stage 0.989 1.015(0.123−8.342)
T classification 0.262 4.441(0.327−60.231)
Tumor thickness 0.724 1.040(0.836−1.293)
Tumor diameter 0.148 0.780(0.556−1.093)
Extrascleral extension (Yes vs. No) 0.891 0.881(0.143−5.436)
Risk score <0.001 5.623(2.687−11.764)
Hazard ratio
B
Training cohort (progression-free survival)
Univariate Cox regression
pvalue
Hazard ratio
Age 0.201 1.023(0.988−1.059)
Gender (Male vs. Female) 0.567 0.790(0.353−1.770)
Stage 0.115 2.023(0.843−4.856)
T classification 0.224 1.616(0.746−3.503)
Tumor thickness 0.669 0.966(0.823−1.133)
Tumor diameter 0.027 1.165(1.017−1.333)
Extrascleral extension (Yes vs. No) 0.012 4.165(1.376−12.612)
Risk score <0.001 2.432(1.766−3.349)
Hazard ratio
Multivariate Cox regression
pvalue
Hazard ratio
Age 0.707 0.990(0.941−1.042)
Gender (Male vs. Female) 0.998 0.999(0.393−2.538)
Stage 0.091 4.047(0.799−20.498)
T classification 0.109 0.209(0.031−1.416)
Tumor thickness 0.271 0.898(0.742−1.087)
Tumor diameter 0.051 1.303(0.998−1.701)
Extrascleral extension (Yes vs. No) 0.686 0.703(0.128−3.871)
Risk score <0.001 2.558(1.658−3.946)
Hazard ratio
C
Validation cohort (metastasis-free survival)
Univariate Cox regression
pvalue
Hazard ratio
Age 0.155 1.027(0.990−1.066)
Gender (Male vs. Female) 0.748 0.865(0.358−2.091)
Tumor side (Left vs. Right) 0.899 1.060(0.433−2.596)
Tumor thickness 0.365 1.130(0.867−1.474)
Tumor location (On equator vs. Others) 0.953 1.033(0.345−3.098)
Tumor diameter 0.543 0.964(0.857−1.085)
Retinal detachment (Yes vs. No) 0.273 1.708(0.656−4.450)
Extrascleral extension (Yes vs. No) 0.133 2.326(0.774−6.992)
Risk score <0.001 1.813(1.315−2.502)
Hazard ratio
Multivariate Cox regression
pvalue
Hazard ratio
Age 0.006 1.093(1.026−1.165)
Gender (Male vs. Female) 0.069 0.370(0.127−1.081)
Tumor side (Left vs. Right) 0.344 0.578(0.185−1.799)
Tumor thickness 0.322 1.164(0.862−1.574)
Tumor location (On equator vs. Others) 0.535 1.541(0.394−6.033)
Tumor diameter 0.585 1.042(0.899−1.207)
Retinal detachment (Yes vs. No) 0.004 11.102(2.191−56.257)
Extrascleral extension (Yes vs. No) 0.249 2.283(0.561−9.301)
Risk score <0.001 2.288(1.545−3.389)
Hazard ratio

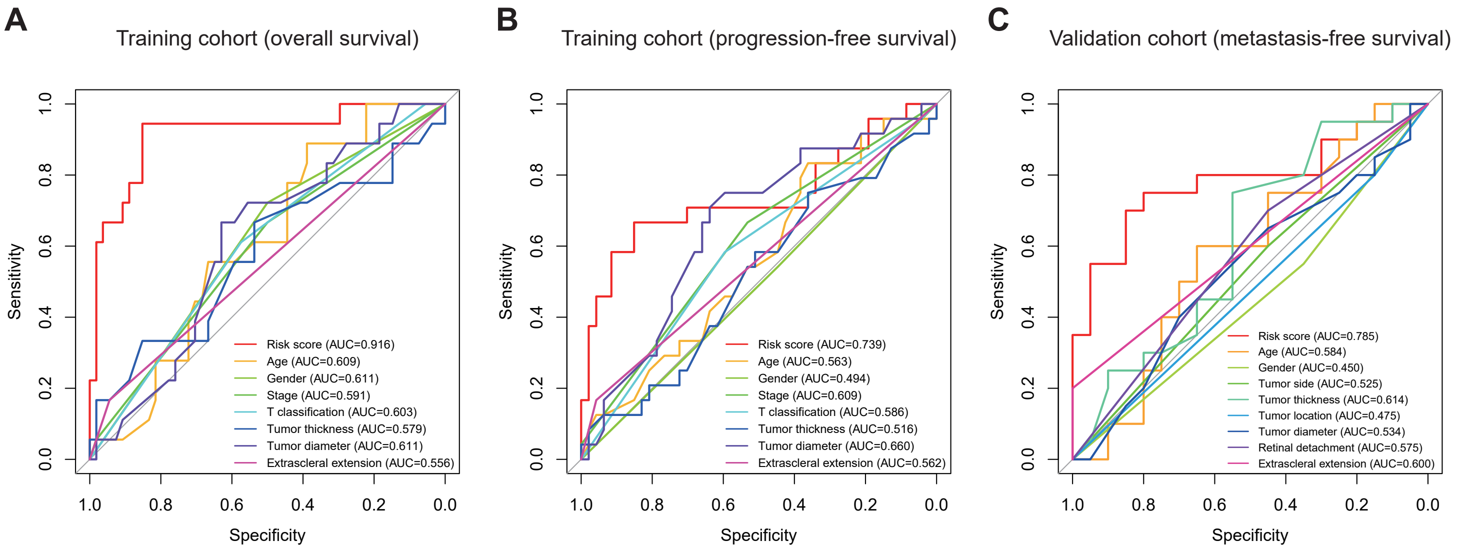
A
Training cohort (overall survival)
Sensitivity
Specificity
1.0
0.8
0.6
0.4
0.2
0.0
Risk score (AUC=0.916)
Age (AUC=0.609)
Gender (AUC=0.611)
Stage (AUC=0.591)
T classification (AUC=0.603)
Tumor thickness (AUC=0.579)
Tumor diameter (AUC=0.611)
Extrascleral extension (AUC=0.556)
B
Training cohort (progression-free survival)
Risk score (AUC=0.739)
Age (AUC=0.563)
Gender (AUC=0.494)
Stage (AUC=0.609)
T classification (AUC=0.586)
Tumor thickness (AUC=0.516)
Tumor diameter (AUC=0.660)
Extrascleral extension (AUC=0.562)
C
Validation cohort (metastasis-free survival)
Risk score (AUC=0.785)
Age (AUC=0.584)
Gender (AUC=0.450)
Tumor side (AUC=0.525)
Tumor thickness (AUC=0.614)
Tumor location (AUC=0.475)
Tumor diameter (AUC=0.534)
Retinal detachment (AUC=0.575)
Extrascleral extension (AUC=0.600)

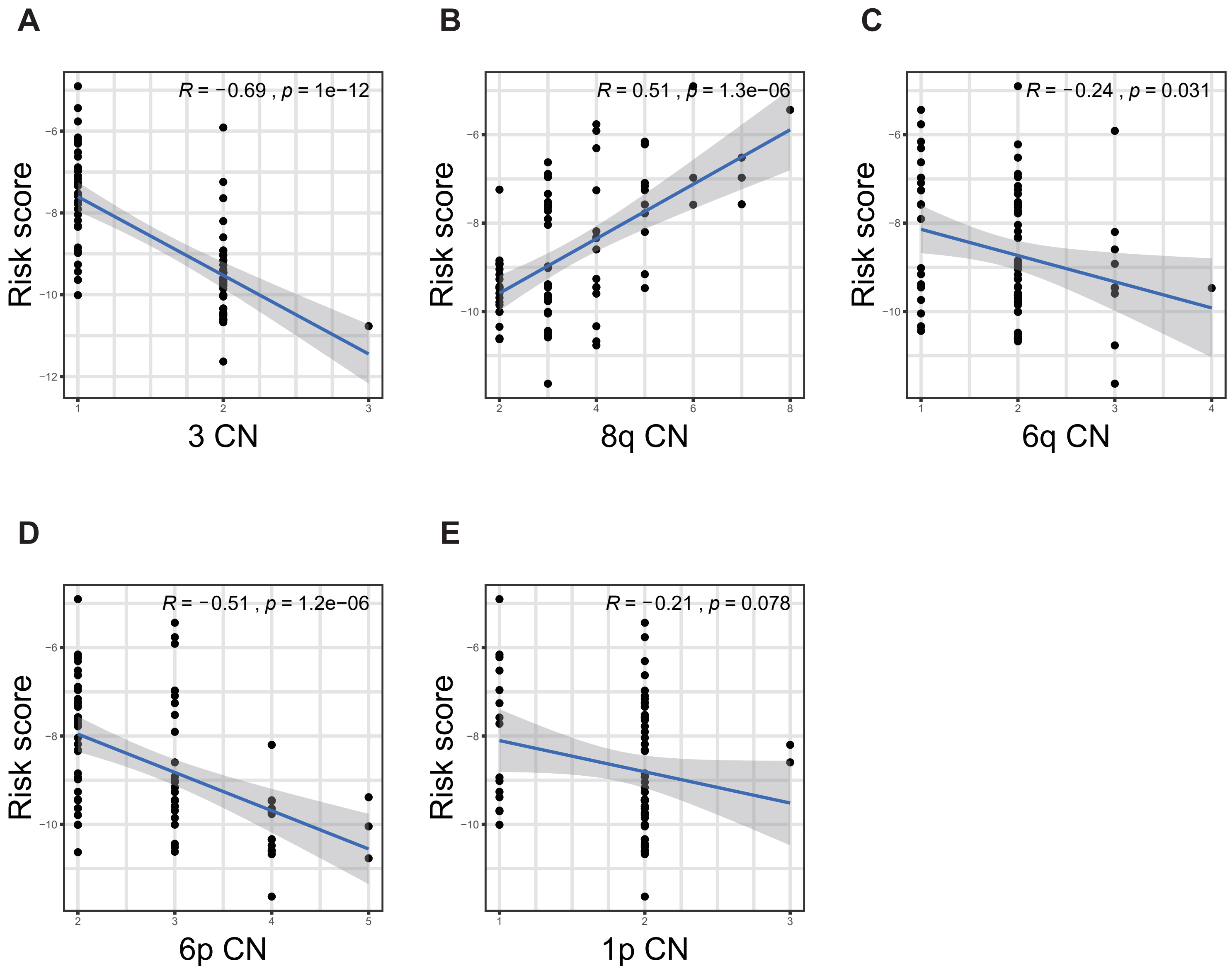
A
R = −0.69 , p = 1e−12
Risk score
3 CN
B
R = 0.51 , p = 1.3e−06
Risk score
8q CN
C
R = −0.24 , p = 0.031
Risk score
6q CN
D
R = −0.51 , p = 1.2e−06
Risk score
6p CN
E
R = −0.21 , p = 0.078
Risk score
1p CN

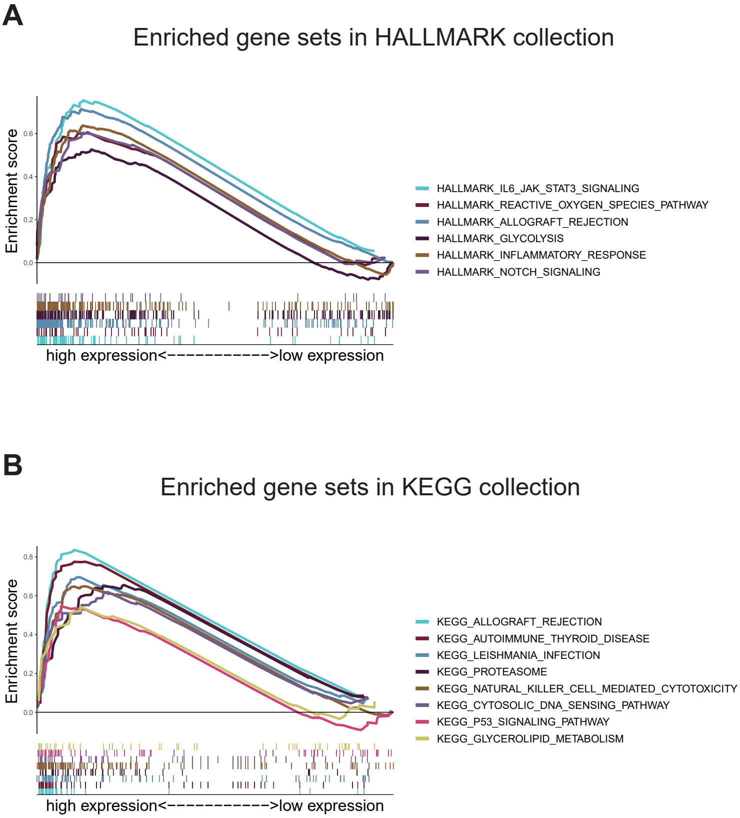
A
Enriched gene sets in HALLMARK collection
Enrichment score
0.6
0.4
0.2
0.0
HALLMARK_IL6_JAK_STAT3_SIGNALING
HALLMARK_REACTIVE_OXYGEN_SPECIES_PATHWAY
HALLMARK_ALLOGRAFT_REJECTION
HALLMARK_GLYCOLYSIS
HALLMARK_INFLAMMATORY_RESPONSE
HALLMARK_NOTCH_SIGNALING
high expression<------------>low expression
B
Enriched gene sets in KEGG collection
Enrichment score
0.8
0.6
0.4
0.2
0.0
KEGG_ALLOGRAFT_REJECTION
KEGG_AUTOIMMUNE_THYROID_DISEASE
KEGG_LEISHMANIA_INFECTION
KEGG_PROTEASOME
KEGG_NATURAL_KILLER_CELL_MEDIATED_CYTOTOXICITY
KEGG_CYTOSOLIC_DNA_SENSING_PATHWAY
KEGG_P53_SIGNALING_PATHWAY
KEGG_GLYCEROLIPID_METABOLISM
high expression<------------>low expression

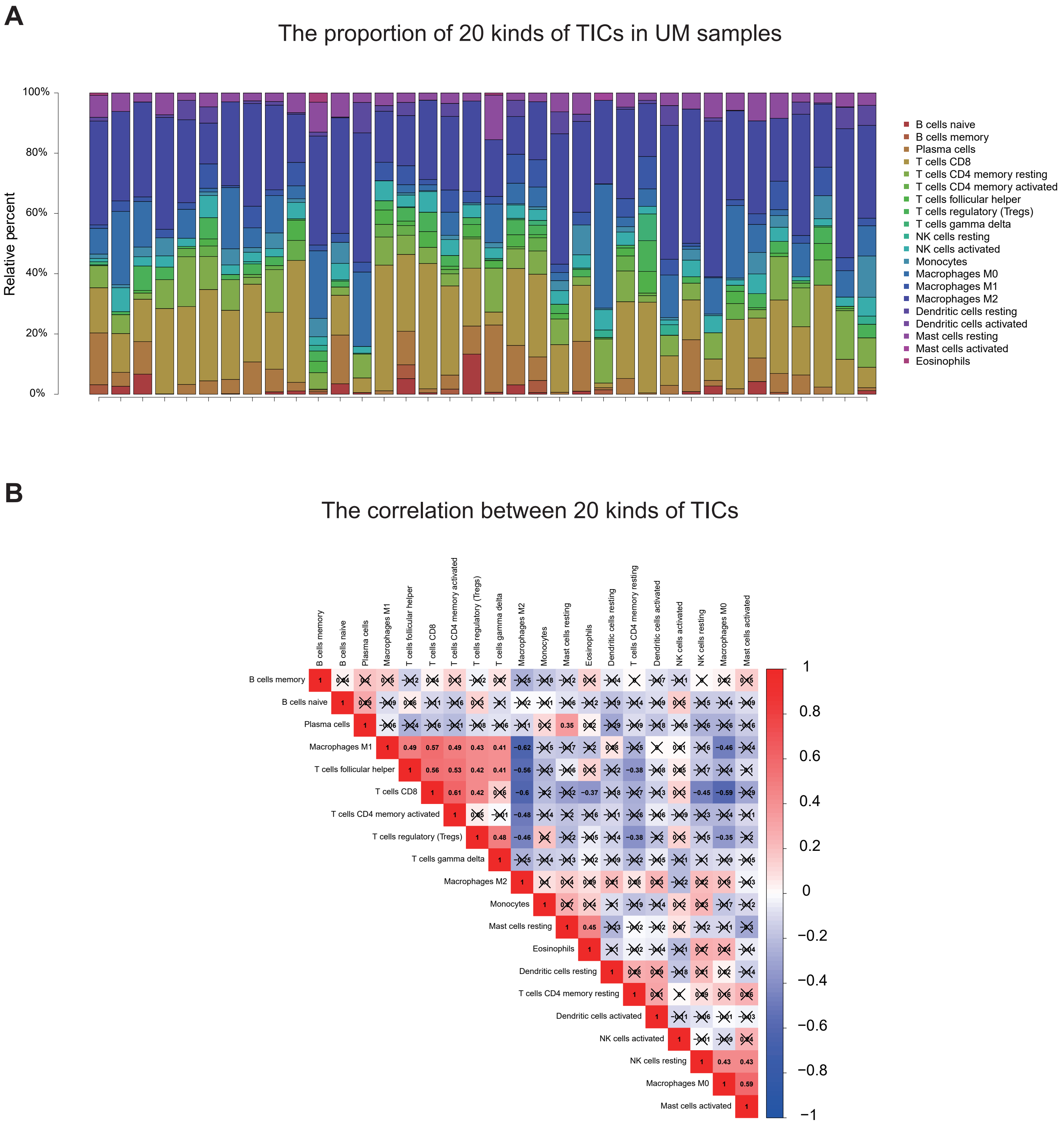
A
The proportion of 20 kinds of TICs in UM samples
Relative percent
100%
80%
60%
40%
20%
0%
B cells naive
B cells memory
Plasma cells
T cells CD8
T cells CD4 memory resting
T cells CD4 memory activated
T cells follicular helper
T cells regulatory (Tregs)
T cells gamma delta
NK cells resting
NK cells activated
Monocytes
Macrophages M0
Macrophages M1
Macrophages M2
Dendritic cells resting
Dendritic cells activated
Mast cells resting
Mast cells activated
Eosinophils
B
The correlation between 20 kinds of TICs
B cells memory
B cells naive
Plasma cells
Macrophages M1
T cells follicular helper
T cells CD8
T cells CD4 memory activated
T cells regulatory (Tregs)
T cells gamma delta
Macrophages M2
Monocytes
Mast cells resting
Eosinophils
Dendritic cells resting
T cells CD4 memory resting
Dendritic cells activated
NK cells activated
NK cells resting
Macrophages M0
Mast cells activated
1
0.8
0.6
0.4
0.2
0
−0.2
−0.4
−0.6
−0.8
−1

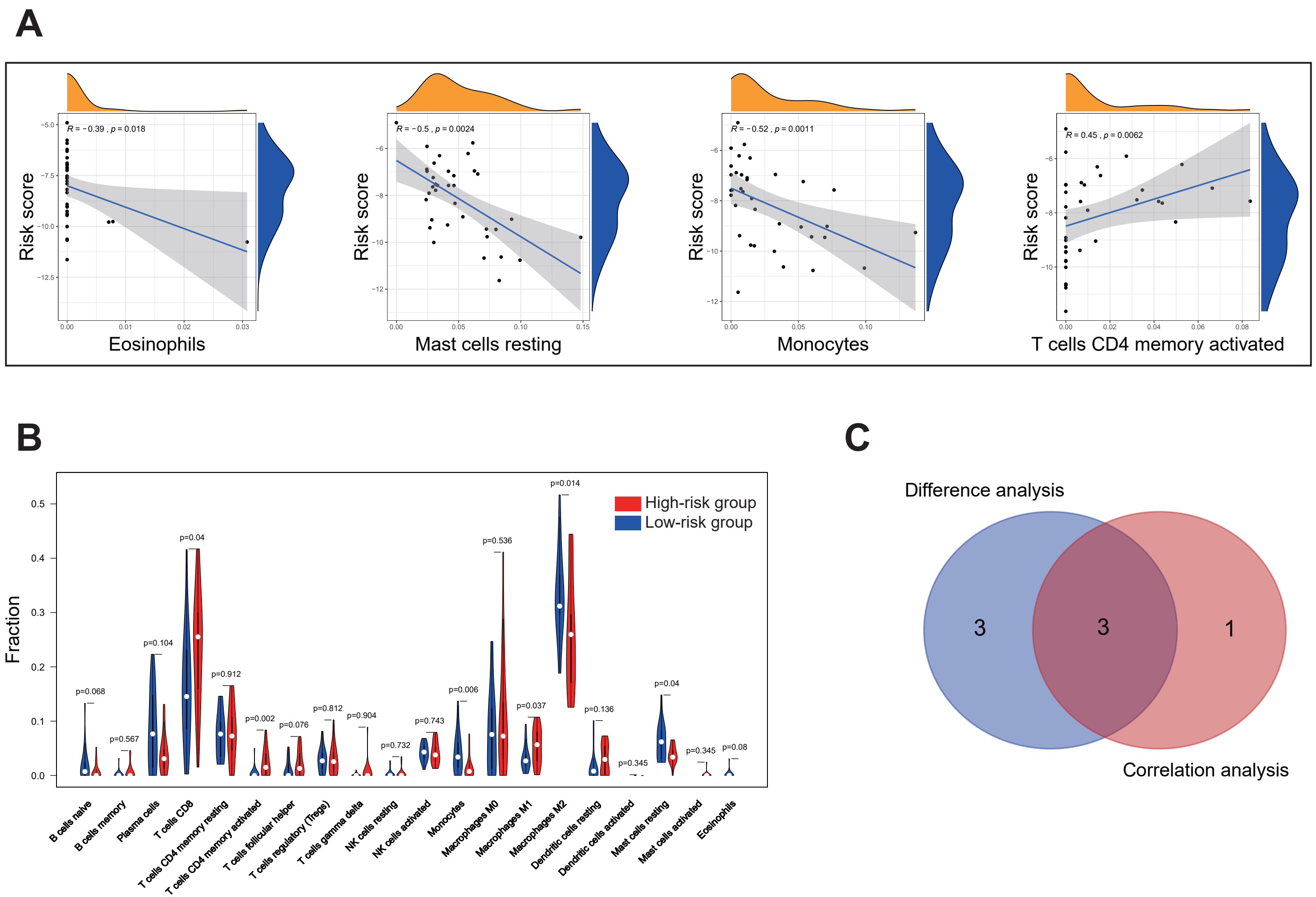
A
R = −0.39 , p = 0.018
Risk score
Eosinophils
R = −0.5 , p = 0.0024
Mast cells resting
R = −0.52 , p = 0.0011
Monocytes
R = 0.45 , p = 0.0062
T cells CD4 memory activated
B
Fraction
High-risk group
Low-risk group
p=0.068
p=0.567
p=0.104
p=0.04
p=0.912
p=0.002
p=0.076
p=0.812
p=0.904
p=0.732
p=0.743
p=0.006
p=0.536
p=0.037
p=0.014
p=0.136
p=0.345
p=0.04
p=0.345
p=0.08
B cells naive
B cells memory
Plasma cells
T cells CD8
T cells CD4 memory resting
T cells CD4 memory activated
T cells follicular helper
T cells regulatory (Tregs)
T cells gamma delta
NK cells resting
NK cells activated
Monocytes
Macrophages M0
Macrophages M1
Macrophages M2
Dendritic cells resting
Dendritic cells activated
Mast cells resting
Mast cells activated
Eosinophils
C
Difference analysis
3
3
1
Correlation analysis
